# Analysis of two domains with novel RNA-processing activities throws light on the complex evolution of ribosomal RNA biogenesis

**DOI:** 10.3389/fgene.2014.00424

**Published:** 2014-12-23

**Authors:** A. Maxwell Burroughs, L. Aravind

**Affiliations:** National Center for Biotechnology Information, National Library of Medicine, National Institutes of HealthBethesda, MD, USA

**Keywords:** rRNA, TSR4, TSR3, 20S, 18S rRNA, tRNA, DTW domain, endosymbiosis

## Abstract

Ribosomal biogenesis has been extensively investigated, especially to identify the elusive nucleases and cofactors involved in the complex rRNA processing events in eukaryotes. Large-scale screens in yeast identified two biochemically uncharacterized proteins, TSR3 and TSR4, as being key players required for rRNA maturation. Using multiple computational approaches we identify the conserved domains comprising these proteins and establish sequence and structural features providing novel insights regarding their roles. TSR3 is unified with the DTW domain into a novel superfamily of predicted enzymatic domains, with the balance of the available evidence pointing toward an RNase role with the archaeo-eukaryotic TSR3 proteins processing rRNA and the bacterial versions potentially processing tRNA. TSR4, its other eukaryotic homologs PDCD2/rp-8, PDCD2L, Zfrp8, and trus, the predominantly bacterial DUF1963 proteins, and other uncharacterized proteins are unified into a new domain superfamily, which arose from an ancient duplication event of a strand-swapped, dimer-forming all-beta unit. We identify conserved features mediating protein-protein interactions (PPIs) and propose a potential chaperone-like function. While contextual evidence supports a conserved role in ribosome biogenesis for the eukaryotic TSR4-related proteins, there is no evidence for such a role for the bacterial versions. Whereas TSR3-related proteins can be traced to the last universal common ancestor (LUCA) with a well-supported archaeo-eukaryotic branch, TSR4-related proteins of eukaryotes are derived from within the bacterial radiation of this superfamily, with archaea entirely lacking them. This provides evidence for “systems admixture,” which followed the early endosymbiotic event, playing a key role in the emergence of the uniquely eukaryotic ribosome biogenesis process.

## Introduction

Ribosomal RNAs (rRNAs) combine with structural proteins in assembly of the ribosome, the ribonucleoprotein protein synthesis complex conserved across the three superkingdoms of cellular life. While there are notable differences between bacteria, archaea, and eukaryotes, the general steps in rRNA production are shared. These broadly entail transcription of a polycistronic precursor, which is then subject to a complex series of processing events involving the interplay between distinct endo- and exo-nucleases (Deutscher, 2009; Mullineux and Lafontaine, 2012; Yip et al., 2013). In eukaryotes, the polycistronic precursor is processed into the mature 18S rRNA transcript, which is assembled into the small ribosomal subunit and the mature 5.8S and 25S/28S rRNA transcripts which are assembled into the large ribosomal subunit. The 5S rRNA transcript in eukaryotes, also incorporated into the large ribosomal subunit, is transcribed independently. In bacteria and archaea, the polycistronic precursor is processed into the mature 16S transcript, which is incorporated into the small subunit and the 5S and 23S rRNA transcripts, which are assembled into the large subunit.

Processing of rRNA precursors in eukaryotes is one of the most complicated RNA-processing events across life, recent counts indicate the number of eukaryotic ribosomal processing factors exceeds 200 (Kressler et al., 2010; Panse and Johnson, 2010). While there has been much progress in the past decade in characterizing rRNA processing and ribosome biogenesis, the intricacies of these processes continue to hamper identification and/or the assignment of precise roles for several of the participating factors (Lafontaine and Tollervey, 2001; Fromont-Racine et al., 2003; Deutscher, 2009; Mullineux and Lafontaine, 2012). Efforts to identify proteins contributing to rRNA maturation pathways have recently turned to large-scale genetic and computational screens (Li et al., 2009; Bellemer et al., 2010). Two proteins identified in such a screen in the yeast *Saccharomyces cerevisiae*, TSR3 and TSR4, were specifically linked to the processing of the 20S rRNA intermediate transcript which gives rise to the mature 18S transcript (Li et al., 2009). In yeast, 20S to 18S maturation is currently known to require the activity of endo- and exo-nucleases including the PIN-domain-containing Nob1, the 5′ → 3′ nuclease domain-containing proteins Xrn1 and Xrn2, and RNase MRP at the A_2_ and D processing sites. Additionally, non-enzymatic factors including Bystin/Enp1 and Nip7, and diverse non-nuclease regulatory enzymes including the methylase Dim1 and various P-loop NTPases (e.g., Fap7) are also involved (Stevens et al., 1991; Lafontaine et al., 1995; Gelperin et al., 2001; Lamanna and Karbstein, 2009; Lindahl et al., 2009; Carron et al., 2011; Morello et al., 2011; Wang and Pestov, 2011; Mullineux and Lafontaine, 2012; Widmann et al., 2012; Loc'h et al., 2014; Zemp et al., 2014). TSR3 has a nearly universal presence in extant organisms and strong sequence conservation across both the eukaryotes and archaea (Armengaud et al., 2005); however, it has rarely been the subject of experimental study. In contrast, orthologs of yeast TSR4, known as the PDCD2/rp-8 and PDCD2L proteins in vertebrates and the Zfrp8 and trus proteins in *Drosophila*, have been frequently studied in the context of a wide range of pathways including apoptosis (Owens et al., 1991; Baron et al., 2010; Ni Nyoman and Luder, 2013), tumorigenesis (Baron et al., 2007; Barboza et al., 2013), cell cycle progression (Minakhina et al., 2007; Chen et al., 2008a; Kokorina et al., 2012), stem cell and other progenitor cell maintenance (Minakhina et al., 2007; Mu et al., 2010; Kokorina et al., 2012; Kramer et al., 2013), piRNA-mediated transposable element silencing (Minakhina et al., 2014), and the inflammation response (Chen et al., 2008b), in addition to being linked to disease progression in Parkinson's (Fukae et al., 2009) and chronic fatigue syndrome/myalgic encephalomyelitis (Kaushik et al., 2005; Zhang et al., 2010). However, the underlying role of TSR4-like proteins in these disparate processes remains unclear, as does the evolutionary provenance and the specific molecular roles played in ribosomal subunit biogenesis by both TSR3 and TSR4.

In an effort to glean further functional insights regarding these proteins, we applied state-of-the-art comparative genome sequence and structure analytical techniques. Our analyses predict an enzymatic role for TSR3, potentially as a novel nuclease, with a role in production of the mature 18S rRNA. We also predict a chaperone-like role for TSR4 in regulating contacts between proteins and potentially rRNA during ribosomal subunit assembly, possibly accounting for the diverse phenotypes linked to TSR4 perturbation.

## Results

### Discovery of bacterial and additional eukaryotic homologs of TSR3

To collect all TSR3 homologs and identify more distant protein relationships, PSI-BLAST searches were run using the entire length of known TSR3 proteins as search seeds. The previously-identified archaeal and eukaryotic TSR3 homologs (Armengaud et al., 2005) were recovered within the first two iterations. In addition to these known homologs, we recovered a set of bacterial sequences with no previous domain annotation and also recovered bacterial and eukaryotic homologs of the DTWD1 and DTWD2 proteins, both of which are annotated in Pfam as containing the functionally uncharacterized DTW domain. For example, a search initiated with the archaeal TSR3 homolog from *Sulfulobus islandicus* (gi: 229585114) recovered uncharacterized bacterial proteins from *Planctomyces brasiliensis* (gi: 325108807, *e*-value: 1e-5, iteration: 2) and *Parachlamydia acanthamoebae* (gi: 338175900, *e*-value: 7e-5, iteration: 3), a DTWD1-like homolog from the ciliate *Tetrahymena thermophila* (gi: 118401887, *e*-value: 0.005, iteration: 5), and a DTWD2-like homolog from the predatory mite *Metaseiulus occidentalis* (gi: 391333458, *e*-value: 0.002, iteration: 7). The above-detected relationships between these previously unlinked sets of proteins were confirmed by reciprocal PSI-BLAST searches and independently using profile-profile comparisons using the HHpred program with hidden Markov models (HMMs) constructed from multiple sequence alignments of the above sets of proteins. For example, a HHpred search initiated with a *Vibrio Cholerae* DTWD2 sequence (gi: 487840886) recovers the pfam DTW HMM profile (*e*-value: 5.9E-55) and the Pfam DUF367 HMM profile (*e*-value: 1.9E-07), which contains several TSR3 homologs. Given these relationships, we named this superfamily the TDD (*T*SR3, *D*TWD1, and *D*TWD2) domain.

Similarity-based clustering of all recovered sequences revealed the presence of five distinct TDD domain families (Supplementary Material) (1) the TSR3-like family universally present in eukaryotes and well-represented across archaea, (2) the previously unrecognized bacterial family named pc1599 after the protein found in *Protochlamydia amoebophila*, predominantly observed in the planctomycetes-verrucomicrobia-chlamydiae superphylum (Wagner and Horn, 2006), (3) the DTWD2-like family present across most eukaryotic lineages including the basal eukaryote *Giardia*, but missing in plants, most fungi, and apicomplexa, (4) the DTWD1-like family broadly present in several bacterial clades including planctomycetes, verrucomicrobia, spirochetes, and proteobacteria and also many eukaryotes including animals, plants, the amoebozoan *Entamoeba* lineage, and scattered presence in apicomplexa and stramenopiles, and (5) the AT1G03687 family, typified by the eponymous *Arabidopsis thaliana* protein, with a patchy representation in eukaryotes including land plants and several other lineages.

### Elucidation of the core TDD domain structure and its distinguishing characteristics

In the Pfam database (Punta et al., 2012), the TSR3 protein is annotated as having a N-terminal RLI (RNase L Inhibitor) metal-binding domain and a C-terminal DUF367 (Domain of Unknown Function 367) domain. The RLI domain was first identified as a potential metal-binding domain with four conserved cysteine residues N-terminal to the RNase L inhibitor (Bisbal et al., 1995), a member of the ABC family of P-loop NTPases. However, since the initial characterization of this region, two crystal structures of these proteins have been experimentally determined (Karcher et al., 2008; Becker et al., 2012). Mapping Pfam RLI domain boundaries onto these crystal structures reveals the RLI domain is part of a larger independently-folding unit which contains a total of eight conserved cysteine residues belonging to the 4Fe-4S dicluster ferredoxin fold which displays two clusters of 4 cysteine residues. The order of secondary elements conserved across this fold is as follows: a single β-strand leading to an α-helix turn followed by a β-hairpin which leads back into a second conserved α-helix and the terminal β-strand which stacks alongside the initial strand. The Pfam RLI “domain,” approximately 35 residues long, encompasses only the initial β-strand and α-helix of the 4Fe-4S ferredoxin domain (Supplementary Material). Further, while the thus-defined RLI domain encompasses the first four conserved cysteine residues of the 4Fe-4S ferredoxin domain, of which only the first two are conserved in just a subset of eukaryotic TSR3-like proteins, the first three cysteine residues combine with the final conserved cysteine of the second cluster to form a single Fe-S cluster. Thus, the RLI domain as currently defined in Pfam represents neither an independently-folding unit nor is capable of coordinating a metal ion by itself, suggesting the RLI domain as presently-defined represents an artificial construct which does not exist as a standalone nor matches the currently available structural information.

**Figure 1:**
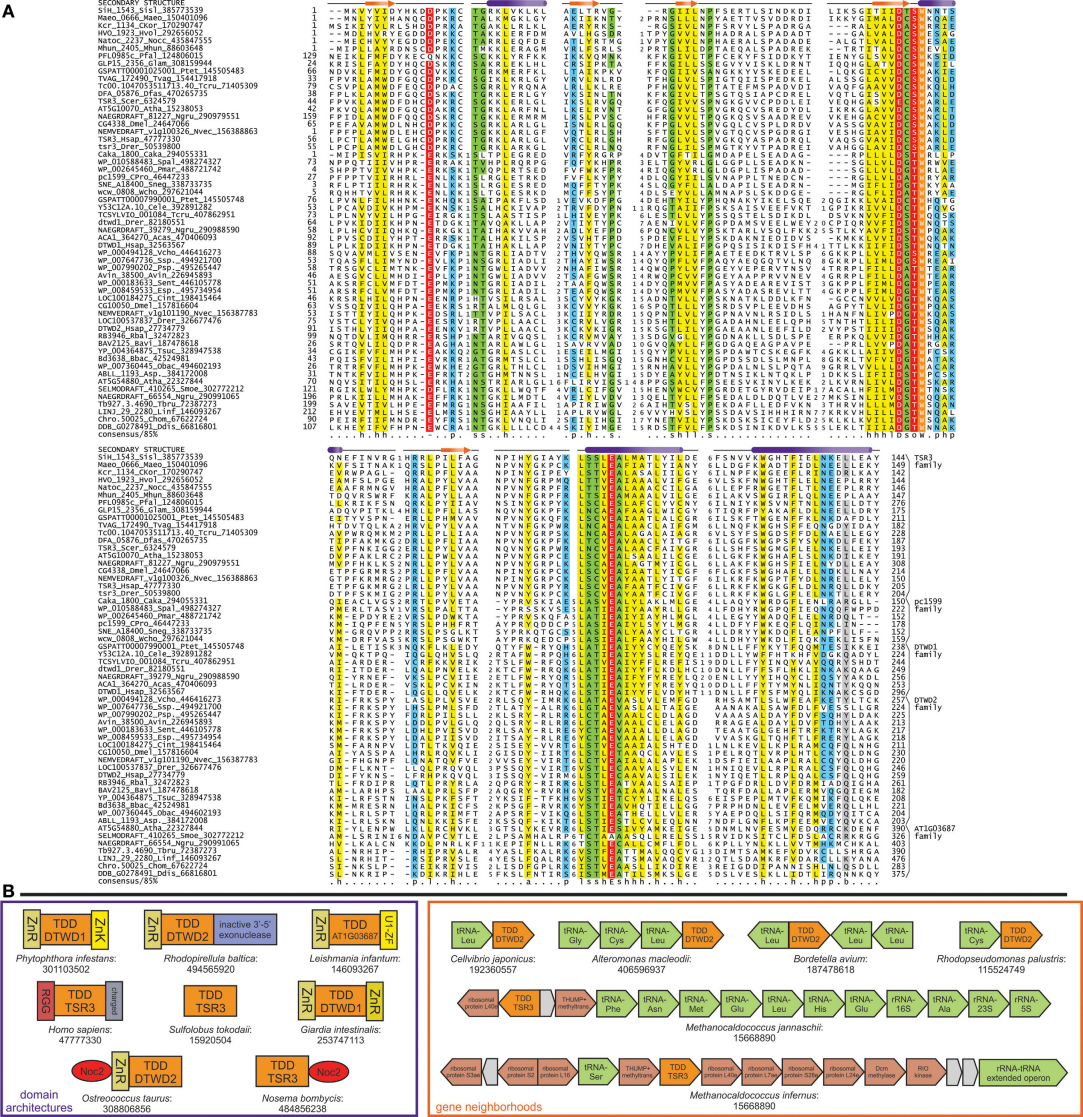
**TDD domain alignment and genome contextual information. (A)** Multiple sequence alignment of the TDD domain, with predicted secondary structure provided at the top of the alignment. Sequences are labeled with gene name, organism abbreviation, and ncbi gene identifier (gi) number; families are annotated to the right of the alignment. Numbers bookending sequences represent positions of the domain within the sequence. Numbers within alignment represent the number of amino acids excised in regions of poor conservation. The alignment is colored based on following consensus: h, hydrophobic shaded in yellow; p, polar in blue; s, small in green; l, aliphatic in yellow; o, hydroxylic; a, aromatic in yellow; b, big in gray. Conserved residues with predicted roles in catalysis are shaded in red and colored in white. The absolutely-conserved tryptophan residue is shaded in orange and colored in white. Organism abbreviations are expanded in Supplementary Material. **(B)** Contextual information for TDD domain. Examples of conserved domain architectures and gene neighborhoods identified for TDD families are boxed in purple and orange, respectively, with the TDD domain always colored in orange. Protein-encoding genes are colored in red and non-coding RNA genes are colored in green. Non-conserved genes within a neighborhood are colored in gray. Domain abbreviations: ZnR, zinc ribbon; ZnK, zinc knuckle; U1-ZF, U1-ribonucleoprotein type C2H2 zinc finger; RGG, arginine/glycine/glycine-rich repeat region.

To further clarify this issue, we built multiple sequence alignments for each of the individual families as well as a superalignment containing representatives from all families defined above (Figure 1A, Supplementary Material). The above steps led us to two salient observations: (1) the predicted secondary structural elements at the N-terminus of the TDD domain are not at all congruent with the secondary structure of the RLI as defined in Pfam and the 4Fe-4S ferredoxin domains based on crystal structures. (2) Consistent with profile-profile comparisons, the region of the TSR3-like proteins mapping to the RLI construct comprises the N-terminal region of the core TDD domain (Supplementary Material). To investigate still further, HMM profiles were constructed from multiple sequence alignments of only the purported RLI region for each of the five families. Of these families, only the region from the TSR3 protein family detected similarity to so-called RLI domain in profile-profile comparisons using HHpred. However, tellingly, in none of the cases comparable searches with the full-length alignment (including that of the TSR3 family) recovered such a match. Thus, one of two scenarios are possible: (1) a “subdomain” of the N-terminal region of the RNase L inhibitor consisting of a strand and helix, which do not directly contact each other, was somehow acquired as an N-terminal fusion and incorporated into the core of the emerging TDD domain and has subsequently diverged beyond recognition in the remaining families or (2) the hit to the RLI domain as presently defined in Pfam represents a spurious match from localized similarity. The former scenario is unlikely given the phyletic patterns (this hit is recovered only by eukaryotic members) and the secondary structure congruence. Hence, the above observations make the RLI annotation in the TSR3 family proteins highly questionable.

Comparison of family-specific alignments and the alignment constructed with representatives from all TDD families reveals a minimal core consisting of five β-strands and four α-helices in an unusual βαβββαβαα order (Figure 1A). The apparent combination of β−α units and a probable three-stranded β-meander is suggestive of a core β-sheet interspersed by 2-3 α-helices. In some families, including TSR3, the C-terminus is predicted to be extended by 1-2 additional helices which are absent in the rest (Supplementary Material), suggesting the C-terminal region could contribute to family-specific functional roles. Several near-universally conserved residues are observed in the TDD core: (1) an aspartate/glutamate residue in the loop between the first β-strand the first α-helix; (2) a DsoW motif at the junction between the third strand and second helix (where “s” indicates a small residue and “o” represents a serine or threonine); (3) a glutamate residue found as part of a larger conserved motif in the N-terminal region of the penultimate helix (Figure 1A). Based on the predicted secondary structure, the multiple strictly-conserved, charged residues have the potential to form a spatially proximal cluster, suggesting the TDD domain functions as an enzymatic domain. Alternatively, these residues could form an active site through dimerization or participate *in trans* during an enzymatic reaction with another RNA-processing enzyme.

### Contextual information suggests a catalytic role for TDD in RNA processing

The gene-neighborhood context within which a gene is embedded is an effective tool for predicting the roles of genes lacking prior characterization by the principle of “guilt by association” (Aravind, 2000; Huynen et al., 2000). We observed that several archaeal orthologs of TSR3 are found in close proximity to various components of the ribosomal super-operon, a collection of protein and rRNA genes with structural and assembly roles relating to the ribosome (Wolf et al., 2001) (Figure 1B). Additionally, in eukaryotic TSR3-like proteins, the TDD domain is almost always fused to a long, N-terminal stretch of arginine/glycine/glycine (RGG) repeats and a highly-negatively charged C-terminal region consisting predominantly of aspartate and glutamate residues (Figure 1B). RGG repeats have a well-established propensity to mediate non-specific RNA interactions in several distinct ribonucleoproteins (Godin and Varani, 2007; Rajyaguru and Parker, 2012). Finally, we identified a striking gene fusion with the ribosomal assembly Noc2 factor in the microsporidian fungus *Nosema bombycis* TSR3 protein, echoed by a similar fusion in the *Ostreococcus taurus* DTWD2 family member (Figure 1B); Noc2 has been implicated in ribosomal RNA maturation processes through co-transcriptional formation of a complex with Noc1, Rrp5, and nascent 35S rRNA precursors and protects pre-ribosomal rRNA from aberrant processing and degradation (Edskes et al., 1998; Milkereit et al., 2001; Nissan et al., 2002; Hierlmeier et al., 2013).

Frequent associations were observed across the multiple TDD domain families (Figure 1B), with various zinc (Zn)-chelating domains (Figure 1B) including the C-terminally fused RNA-binding U1-ribonucleoprotein-type C2H2 Zn-finger (Du and Rosbash, 2002) in kinetoplastid versions of the AT1G03687 family and the Zn-knuckle in stramenopile representatives of the DTWD1 family. Additionally, several planctomycetes and δ-proteobacteria members of the DTWD2 family are fused to a catalytically inactive version of the 3′ → 5′ exonuclease domain of the RNase H fold (Figure 1B). Inactive versions of enzymatic domains often acquire a secondary binding function (del Sol et al., 2006), suggesting these domains could function as RNA-binding domains. Finally, in bacteria, we also observed gene-neighborhood associations of the DTWD2 family with tRNA genes in several phylogenetically distant species pointing to a possible role in tRNA-processing in these organisms (Figure 1B, Supplementary Material).

Thus, the sum of the evidence presented above from contextual associations with (1) rRNA/ribosomal genes in archaea and tRNA genes in bacteria, (2) RNA-binding or rRNA maturation-related domains, along with the previously reported gene-deletion and high-throughput data analysis on yeast TSR3 (Li et al., 2009) implicate TDD domain proteins in directly interacting with different RNAs. The potential functional displacement of the 3′ → 5′ exonuclease in certain bacteria along with the character of the strictly-conserved, predicted active site residues noted in the previous section (Figure 1A) specifically point toward a potential RNase function for the TDD domain. This in turn suggests that the TSR3 family of TDD domains might function as RNases contributing to the processing of mature 18S rRNA in archaea and eukaryotes. However, given the presence of several distinct enzymes in the ribosome maturation system, we cannot entirely rule out other potential activities (Anantharaman et al., 2002).

### Redefinition of the TSR4 domain structure and discovery of its bacterial homologs

The yeast TSR4 protein is annotated as having the PDCD2_C domain at its C-terminus in the Pfam database (Punta et al., 2012). We detected a region of low complexity in the center of the TSR4 protein bounded by the C-terminal PDCD_2 domain and an additional, uncharacterized N-terminal globular region. Searches initiated with this N-terminal globular region recovered bacterial homologs lacking any domain annotation. For example, a search initiated with the yeast TSR4 N-terminal region recovered proteins in *Acinetobacter* sp. (gi: 497271131, *e*-value: 6 × 10^−3^, iteration: 2), *Campylobacter showae* (gi: 489043535, *e*-value: 7 × 10^−3^, iteration: 2), and *Streptomyces camus* (gi: 518968996, *e*-value: 10^−3^, iteration: 3). Reverse searches initiated with these proteins recovered a range of bacterial homologs as well as the TSR4 protein in eukaryotes. Further searches with the bacterial proteins also recovered a new set of bacterial homologs with the detected region of similarity overlapping with the Pfam model annotated as DUF1963. For example, a search initiated with the same region from the above *Campylobacter showae* sequence recovered TSR4 homologs in mouse (gi: 120407033, *e*-value: 9 × 10^−7^, iteration: 2) and DUF1963-containing homologs in *Haliscomenobacter hydrossis* (gi: 332665028, *e*-value: 5 × 10^−3^, iteration: 6). Continuing these searches we recovered the YwqG protein in *E. coli* and its homolog from *Bacillus subtilis* for which a crystal structure was solved by the Protein Structural Initiative (Montelione, 2012) (Protein Data Bank identifier: 1PV5). All sequences recovered in the above searches were unified by two well-conserved features: (1) a motif typically taking the form of GGxP (“x” being any residue) and (2) a highly-conserved Q residue. A subset of the sequences additionally contained a pair of CxxC motifs that are likely to constitute a metal-binding site.

**Figure 2:**
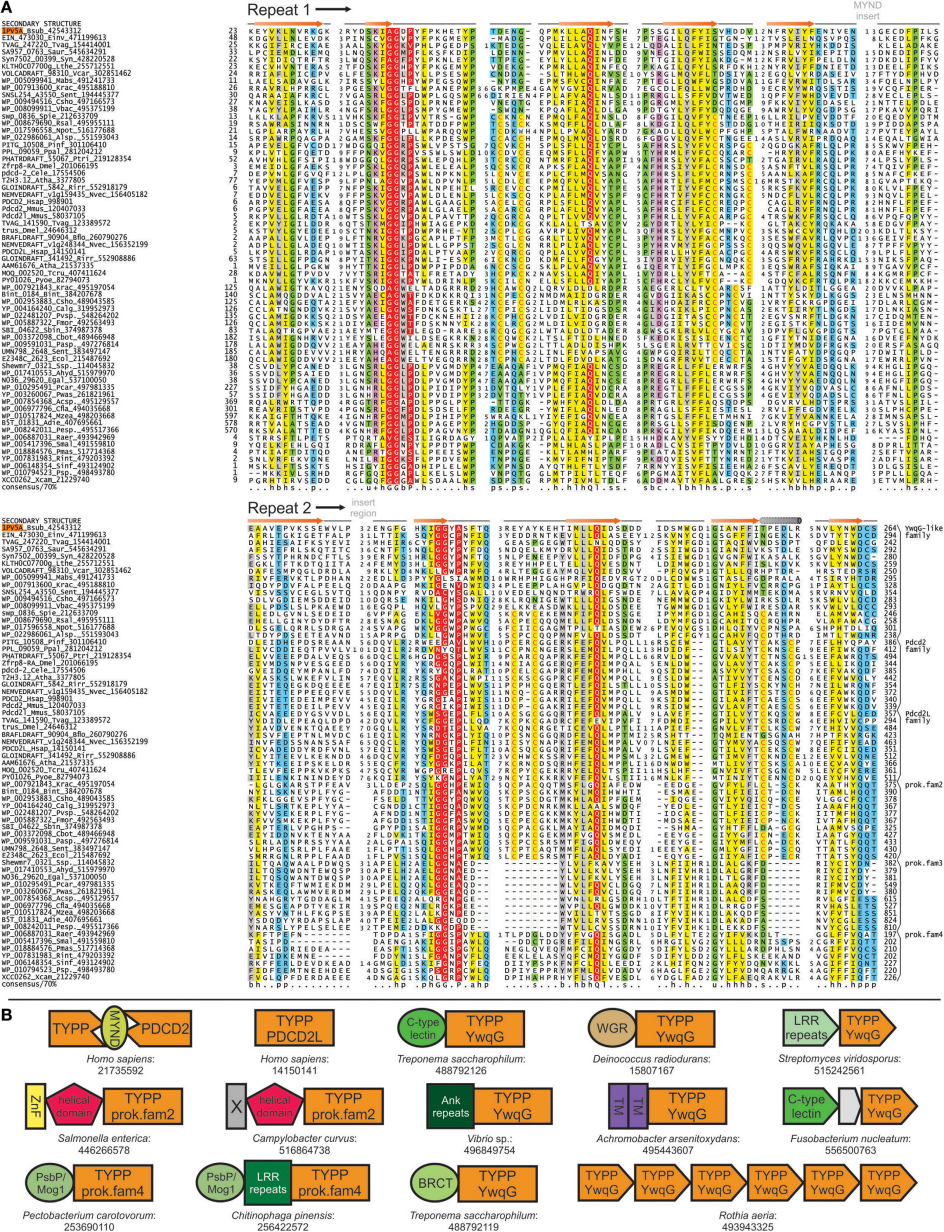
**TYPP domain alignment and genome contextual information. (A)** Multiple sequence alignment of the TYPP domain; formatting and coloring scheme same as Figure 1A. Additionally: u, tiny shaded in green; +, positively-charged in purple; c, charged in purple. Sporadically-conserved CxxC motifs are highlighted. Insert sites and starting points of the two repeats are labeled above secondary structure in gray and black, respectively. Secondary structure is based on the crystal structure of 1PV5. Organism abbreviations are expanded in Supplementary Material. **(B)** Genome contextual information for TYPP domain. Conserved domain architectures and gene neighborhoods formatted as in Figure 1B. Additional abbreviations: Ank, Ankyrin; TM, transmembrane helix.

Profile-profile comparison searches performed with HHPred and initiated with the yeast TSR4 N-terminal region confirmed a relationship with the DUF1963 domain (*p*-value: 3.2 × 10^−10^) but also detected a significant relationship with the PDCD2_C domain (*p*-value: 3.8 × 10^−9^), suggesting that TSR4 proteins and their homologs contain two copies of a single domain that underwent an ancestral duplication. This was supported by the recovery of the GGxP-like motif, the Q, and the pair of CxxC motifs in alignments of both the N- and C-terminal regions. In addition, both PSI-BLAST and HMM searches run with multiple full-length bacterial versions as seeds retrieved matches to eukaryotic sequences extending along the entire length of the TSR4-like protein barring the low-complexity insert. Finally, profile-profile comparisons initiated with the full-length bacterial sequence from *Salmonella enterica* as a seed (gi: 555248518) recovers the DUF1963 and PDCD2_C profiles at both the N- and C-terminal repeats (DUF1963 full-length match, *p*-value: 1.1E-14; DUF1963 C-terminal match, *p*-value: 2.4E-09; PDCDC_2 N-terminal match, *p*-value: 6E-06; PDCDC_2 C-terminal match, *p*-value: 3.2E-06). The duplication was further confirmed via examination of the structure of the bacterial version (1PV5) (see below). Thus, Pfam PDCD2_C and DUF1963 are models partially covering the same superfamily of proteins with the former only covering part of the C-terminal repeat. We named the unified and correctly defined superfamily of domains encompassing both repeats as TYPP (after the *T*SR4, *Y*wqG, *P*DCD2L, and *P*DCD2 proteins; Figure 2A).

**Figure 3:**
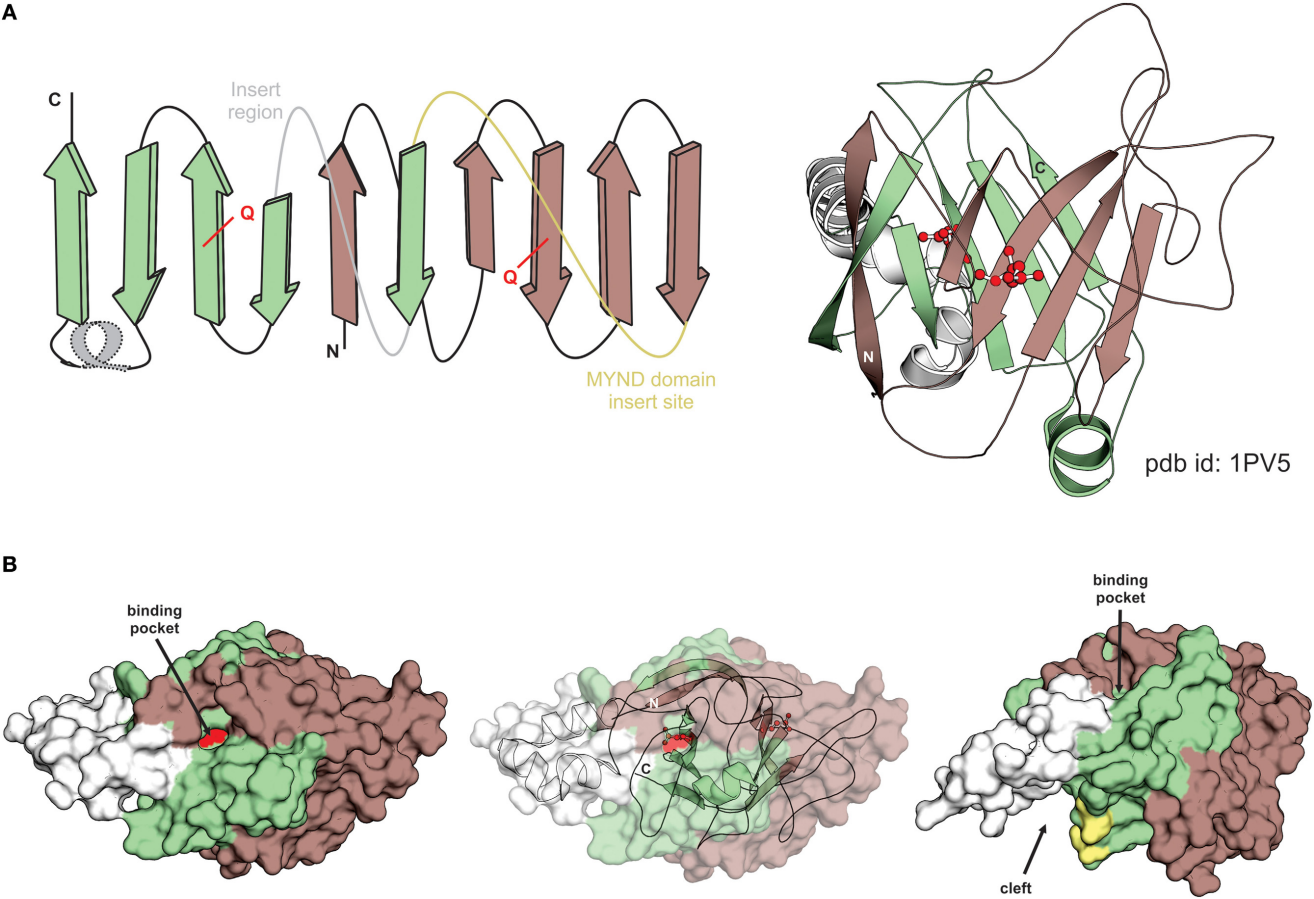
**Structural overview and features of TYPP domain. (A)** Topology diagram of TYPP domain provided to the left. Strands from the N- and C-terminal repeats are respectively colored in dark red and green. The loop corresponding to the standard insert region and the MYND domain insert are colored in gray and yellow, respectively. The well-conserved Q residues in each repeat is marked in red. The poorly-conserved helical segment is shown as a dotted line and colored in gray. Cartoon rendering of the TYPP domain (pdb id: 1PV5) is given to the right. Coloring the same as topology diagram. **(B)** Molecular surface renderings of TYPP domain. Surfaces are colored by repeat congruent to **(A)**. Predicted binding pocket view shown on left. Middle rendering overlays same view on the cartoon depiction. Right view shows the contribution of the insert region to the cleft with polar residues lining the cleft colored yellow.

Examination of the structure of the *B. subtilis* version revealed that the five stranded β-sheets formed by the two repeats stack against each other at a roughly 60 degree orientation (Figure 3A). A multiple sequence alignment of all detected members (Figure 2A) indicated that the loop region following the first strand of the second repeat is the preferred site for inserts in the superfamily ranging from minimal elaborations observed in certain bacterial members to the large region of low complexity in eukaryotic TSR4 proteins (Figure 2A). The two sheets are made up of strands from the same repeat barring the first strand which is swapped with the other repeat. The surface of the TYPP domain revealed two distinctive features (Figure 3B): (1) a deep pocket with the nearly absolutely-conserved Q residue from the second repeat at its base and (2) a cleft formed between the insert and the second repeat lined by conserved polar residues (Figure 3B). Sequence similarity-based clustering identified six distinct families of TYPP domains: (1) the YwqG family (named for the *E. coli* protein) widely distributed across bacteria, including the solved crystal structure from *B. subtilis*, and also found in a small group of eukaryotes; (2) the PDCD2L family found across all eukaryotes and including the yeast TSR4 protein; (3) the PDCD2 family found across plants, animals, fungi, slime molds and certain stramenopiles; (4) three additional, relatively narrowly-distributed bacterial families numerically labeled 2–4 (see Supplementary Material for complete lists of members in the families).

### Contextual associations and functional inference for TYPP domains

To further understand the functions of TYPP domains we queried currently available interaction networks from different organisms. Members of both the PDCD2 and PDCD2L families recovered strong associations with ribosomal subunit assembly pathway components in human, mouse, and *Drosophila*, similar to the associations reported earlier for the TSR4 protein in yeast (Li et al., 2009) and consistent with their expression across most tissue types (Ramalho-Santos et al., 2002). All PDCD2 family TYPP domains contain an insertion of the MYND domain, a Zn-chelating, bi-nuclear treble clef fold domain (Owens et al., 1991; Scarr and Sharp, 2002) (Figure 2B), just downstream of the final strand of the first repeat (Figures 2A, 3A). The MYND domain, like many treble clef fold-containing domains (Burroughs et al., 2011), is a protein-protein interaction (PPI) domain functioning in diverse contexts (Liu et al., 2007; Matthews et al., 2009). Most family two members are fused to an uncharacterized N-terminal α-helical domain, with several additionally N-terminally linked to the C4-type Zn finger of the dksA/traR family (Figure 2B). This family of Zn fingers directly interacts with RNA polymerase (Paul et al., 2004; Perederina et al., 2004; Blankschien et al., 2009; Tehranchi et al., 2010; Satory et al., 2013); thus, similar to the MYND domain, dksA/traR might represent a PPI domain.

Across phyletically-diverse bacteria, family four TYPP domains are fused to N-terminal PsbP/Mog1 domains, LRR repeats, or both (Figure 2B, Supplementary Material). LRR repeats form concave surfaces mediating PPIs in several contexts (Kobe and Kajava, 2001; Kedzierski et al., 2004), while a version of the PsbP/Mog1 domain has recently been implicated as an adaptor mediating PPIs between secreted toxin systems and the type-VI secretion apparatus (Zhang et al., 2012). The YwqG family shows several N-terminal domain fusions sporadically present across phyletically-diverse bacteria (Figure 2B). Among these, several have been linked to peptide/protein binding including a domain of the C-type lectin fold (Zelensky and Gready, 2005; Carlson et al., 2008; Iyer et al., 2009), ankyrin repeats (Mosavi et al., 2004), and the BRCT domain (Leung and Glover, 2011; Gerloff et al., 2012). YwqG family members are also fused to transmembrane (TM) helical regions and the predicted nucleic acid-binding WGR domain, also found in polyADP ribose polymerases (Hassa et al., 2006; Citarelli et al., 2010). Additionally, multiple copies of the YwqG-like TSR4 proteins are observed clustering together in a single operon in several organisms (Figure 2B, Supplementary Material), suggesting TYPP could generally function as a multimer.

These contextual connections suggest a general role for the TYPP domain in mediating PPIs with other domains, perhaps as part of a multi-component protein complex. This is consistent with the striking channel observed in the TYPP domain structure: it could function in binding poorly-structured regions or extended peptides. Several of the linked domains above, including the PsbP/Mog1 and C-type lectin domains, show domain fusion-associations with enzymatic domains related to peptide modification and processing. Based on this contextual analogy a more radical interpretation would be a catalytic role for the TYPP domain. Under this interpretation structural features of the TYPP domain, such as the cleft and the predicted binding pocket harboring the conserved glutamine might not just serve as a PPI interface but as a catalytic active site. One conceivable enzymatic role could entail peptide modification; however, such a reaction with only the well-conserved Q residue currently has little enzymological precedent. Hence, a more plausible explanation is that the TYPP domain performs a chaperone-like function in facilitating specific PPIs during assembly of protein complexes. In the case of the eukaryotic version involved in ribosomal biogenesis, such interactions could augment or modify activity of RNA-processing enzymes (e.g., the predicted TDD domain nuclease) via a chaperone-like action. A precedent for this is offered by the archease domain with an analogous two-repeat structure (Anantharaman and Aravind, 2004), which enhances the specificity of different RNA-modifying enzymes, such as tRNA cytosine methylases and RtcB-like tRNA ligases, via chaperone-like action (Auxilien et al., 2007; Desai et al., 2014).

## Discussion

### Evolutionary and functional implications of the TDD and TYPP domains for ribosomal biogenesis

The above characterization of the TDD and TYPP domains has several implications for the early evolution of the eukaryotic ribosomal biogenesis system. First, discovery of a distinctly bacterial clade of TDD domains suggests that a single copy of this domain can now confidently be assigned to the Last Universal Common Ancestor (LUCA) of Life. Given the presence of a strongly-supported archaeo-eukaryotic clade of TDD domains, it is likely that the ancestral version of this clade acquired rRNase function. In contrast, associations of the dominant bacterial family of TDD domains, DTWD2, suggests acquisition of a tRNA-processing role. The Last Eukaryotic Common Ancestor (LECA) can be inferred as possessing two distinct versions of the TDD domain: a cognate of TSR3 closest to the archaeal cognates and a DTWD1 family representative, which is closest to the bacterial DTWD2 family. The two copies were therefore likely respectively acquired from the archaeal and bacterial progenitors participating in the primary endosymbiotic event leading to eukaryogenesis. Beyond these, additional eukaryotic versions were likely transferred later from bacteria and recruited for as-yet-uncharacterized RNA-processing events.

The prediction of nuclease function for TSR3 suggests interesting possibilities for the highly-coordinated endo-/exo-nucleolytic rRNA maturation events in which it is implicated (Mullineux and Lafontaine, 2012). Experimental evidence linking TSR3 to 20S intermediate generation (Li et al., 2009) is consistent with the archaeo-eukaryotic history of TSR3: 20S is derived via processing at the internal transcribed spacer 1 (ITS1) site which is conserved across eukaryotes and archaea. In yeast, cleavage at the “D” site yields 20S intermediates. Although the PIN domain nuclease Nob1 has been implicated in D site cleavage in yeast (Lamanna and Karbstein, 2009), the persistent, albeit low-level, presence of the 20S and 18S intermediates in Nob1 negative mutant strains suggests that Nob1 may not be the sole nuclease involved in this cleavage (Fatica et al., 2004). Hence, a possible role for the TSR3 family would be nuclease action at this step. While convergent evolution of site-specific endonucleases is less likely to emerge than exonucleases, the fundamental importance of rRNA processing to the cell could favor functional backup in this instance. Examples of known (exo)nuclease backup include recruitment of the same nuclease for multiple cleavage steps [e.g., RNase MRP (Schmitt and Clayton, 1993; Lindahl et al., 2009) or Rrp17 (Oeffinger et al., 2009)] and multiple nucleases recruited for cleavage at the same site [e.g., Rat1-Rai1 (Henry et al., 1994) and Rrp17 (Oeffinger et al., 2009) in B1S site trimming in yeast]. Thus, entire alternative pathways generating the same or similar intermediates appear to have been favored in evolution. Given this, TSR3-like proteins could play a role in D site or another site during rRNA maturation. It is also worth noting that several non-nuclease enzymes have also been implicated in 18S maturation, often through modification of other key players in the pathway including various NTPase, methylases, and kinases; perturbation of these can influence 18S and 20S levels in the cell (Lafontaine et al., 1995; Gelperin et al., 2001; Widmann et al., 2012; Loc'h et al., 2014; Zemp et al., 2014). Hence, we cannot entirely rule out a more ancillary enzymatic role for TSR3 in 18S maturation.

The current analysis also shows that the TYPP domain has an evolutionary history distinct from the TDD domain. The broad bacterial distribution of the TYPP domain, along with its absence in archaea, indicates a provenance in bacteria followed by lateral transfer to basal eukaryotes. This ancestral eukaryotic version gave rise to the PDCD2L family (containing TSR4) which, upon duplication and insertion of the MYND domain, gave rise to the paralogous PDCD2 family prior to the divergence of animals, fungi, and plants from their common ancestor. Our findings suggest both of these eukaryotic paralogs are involved in ribosomal biogenesis, an avenue of research which has been largely neglected in studies on Drosophila and mammalian orthologs and a functional assignment which could account for the diverse consequences observed following its perturbation. Additional sporadic transfers of the TYPP domain from bacteria to terminal eukaryotic lineages have also been observed (Rolland et al., 2009). The distinct TDD/TYPP evolutionary histories suggests these two key players in eukaryotic rRNA processing and ribosomal biogenesis with similar mutant phenotypes were acquired respectively from the archaeal and bacterial progenitors of the eukaryote, most probably during the primary endosymbiosis. Importantly, this indicates the complex eukaryotic-specific elements of ribosomal RNA processing and ribosome biogenesis are a product of the coming together of bacterial and archaeal heritages in the same cell.

It has been previously proposed that as the endosymbiotic event proceeded, mis-interactions between bacterial and archaeal ribosomal proteins could have been triggered in the cytoplasm. The emergence of the nucleus and the nucleolar center for ribosome biogenesis is likely evolutionarily correlated with this problem (Jekely, 2008). Additionally, the distinct, tightly-regulated rRNA processing and ribosome assembly pathways likely contributed to admixture prevention between the two ancestral ribosome types (Johnson et al., 2002; Panse and Johnson, 2010). In this context, the proposed chaperone-like activity of the TYPP domain might have been recruited for eukaryote-specific rRNA processing events. It is possible TYPP may have acquired chaperone-like functions outside of rRNA processing in eukaryotes as suggested by its interactions with Maelstrom of the piRNA pathway (Minakhina et al., 2014) and involvement in chromatin associated complexes via binding of the host cell factor-1 (HCF-1) and potentially the N-CoR/Sin3A transcriptional coactivator complex (Scarr and Sharp, 2002).

### General conclusions

The above results extend our understanding of RNA processing in both functional and evolutionary terms. First, we provide the testable hypothesis that the TDD domain (including TSR3) is a nuclease required for rRNA processing in archaea and eukaryotes and possibly tRNA processing in bacteria. We also present the hypothesis that TSR4 might play a role in augmenting PPIs, foremost in ribosome biogenesis, and potentially in additional contexts. In evolutionary terms, we detect the first bacterial homologs of these conserved proteins. As a result, we obtain clear evidence that the provenance of the unique and complex ribosome biogenesis system of eukaryotes necessarily required the coming together of bacterial and archaeal components. This offers further support to the growing evidence that the consequences of “systems admixture” following the primary endosymbiotic event strongly contributed to the emergence of quintessential eukaryotic features.

## Materials and methods

Iterative sequence-profile and HMM searches were performed using the PSI-BLAST (Altschul et al., 1997) and JACKHMMER web utilities (http://hmmer.janelia.org/search/jackhmmer), respectively. Queries were run against the non-redundant (nr) protein database of the National Center for Biotechnology Information (NCBI). For most sequence-based homology searches, which underlie the relationships presented in this work, a cut-off *e*-value of 0.01 was used to assess significance. In each iteration, newly-detected sequences included within the cut-off were evaluated via initiation of a new search with the sequence in question as the query to guard against inclusion of false positives; searches were continued with the same *e*-value threshold only if the profile remained uncorrupted without false positives. Postulated relationships recovered using iterative searches were further confirmed with other aids such as concordance of predicted or known secondary structural elements. Profile-profile comparisons were also used as an additional means of confirming distant relationships, these were performed using the HHpred program (Soding et al., 2005).

Sequence-based homology clustering of TDD and TYPP proteins and associating proteins in gene neighborhoods was performed with the BLASTCLUST program (http://ftp.ncbi.nih.gov/blast/documents/blastclust.html), using empirically-determined length and score threshold cut-off values. Multiple sequence alignments of resulting clusters were constructed using the MUSCLE alignment program (Edgar, 2004) followed by manual adjustment informed by sequence-based homology search results and experimentally-determined structures. Secondary structure predictions of resulting alignments were performed with the JPred program (Cuff et al., 1998). Structure similarity searches were performed using the DaliLite program (Holm et al., 2008). Visualization and manipulation of protein structure was accomplished using the PyMol program (http://www.pymol.org). Automatic aspects of large-scale analysis of sequences, structures, and genome context were performed with the in-house TASS package, which comprises a collection of Perl scripts.

For each gene of interest recovered in homology searching, the gene neighborhood was comprehensively interrogated using custom Perl scripts from the TASS package. These scripts utilize PTT files (retrieved through the NCBI ftp site) when the gene is from an assembled genome or Genbank files when the gene is from a collection of whole genome shotgun sequences. After locating the gene, a default value of the five nearest neighbors in both directions are extracted, this value is altered on rare occasions when the gene in question is part of a long, extended neighborhood. Protein sequences of all neighbors are clustered using the BLASTCLUST program (ftp://ftp.ncbi.nih.gov/blast/documents/blastclust.html) to identify related sequences in gene neighborhoods. Each resulting gene cluster is then assigned annotation based on the shared domain architecture or single domain in the encoded protein. This allows an initial annotation of gene neigborhoods which is further refined by including only genes which are unidirectional on the same strand of DNA and share a putative common promoter, identified by assigning a maximum distance between adjacent genes, with the default assigned as 150 nucleotides. “Head-to-head” gene arrangements on opposite strands are also included when potential bidirectional promoter sharing patterns are detected.

### Conflict of interest statement

The authors declare that the research was conducted in the absence of any commercial or financial relationships that could be construed as a potential conflict of interest.
