# Supplementary material for: Analysis of two domains with novel RNA-processing activities throws light on the complex evolution of ribosomal RNA biogenesis
Source: Front Genet. 2014 Dec 23;5:424. doi: 10.3389/fgene.2014.00424 (PMC4275035; doi:10.3389/fgene.2014.00424)
Supplement: Supplementary file 1 [file DataSheet1.ZIP › Supplemental_Figure.pdf]

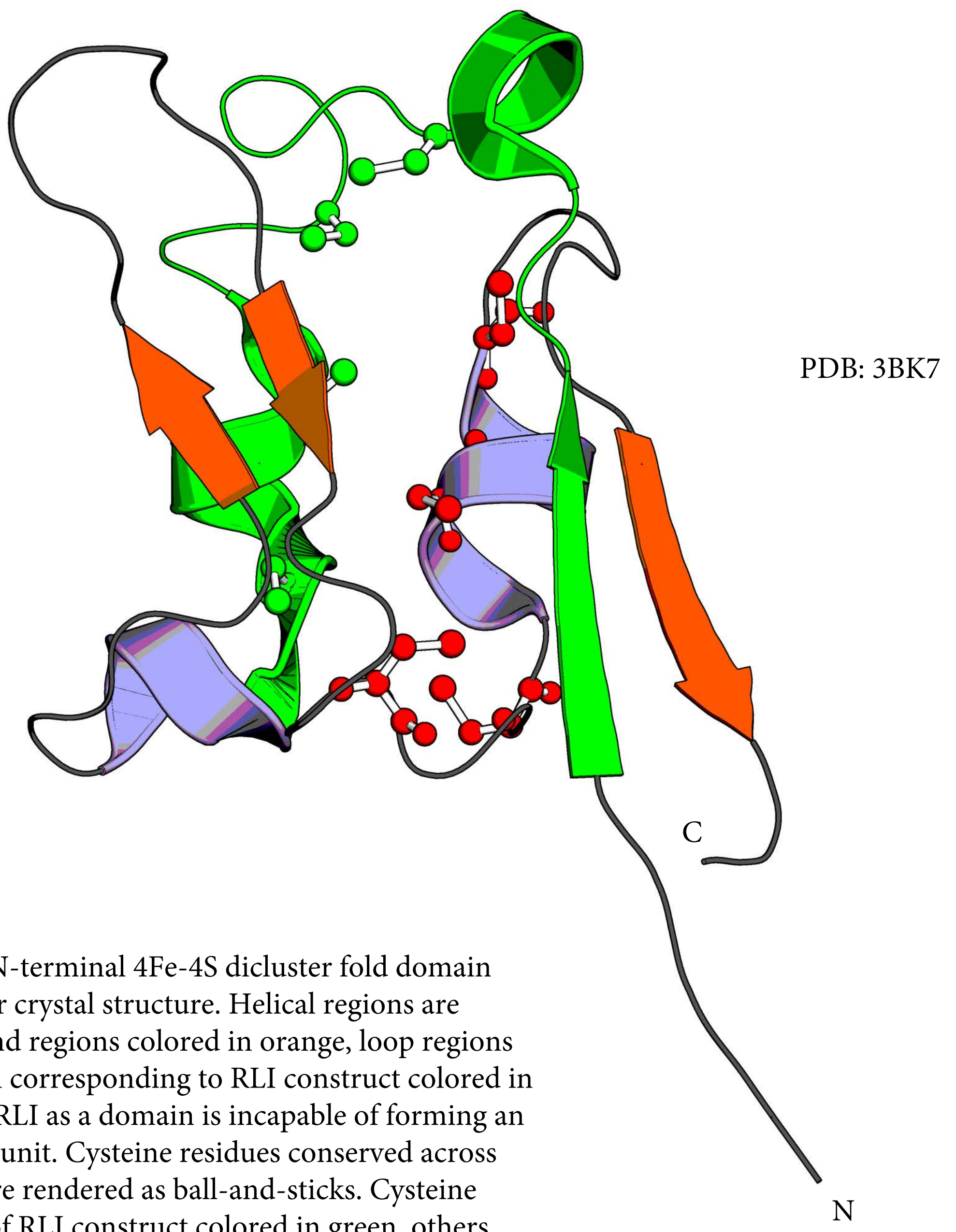

Supplemental Figure
Cartoon rendering of N-terminal 4Fe-4S dicluster fold domain from RNase L Inhibitor crystal structure. Helical regions are colored in purple, strand regions colored in orange, loop regions colored in gray. Region corresponding to RLI construct colored in green. As can be seen, RLI as a domain is incapable of forming an independently-folding unit. Cysteine residues conserved across 4Fe-4S dicluster fold are rendered as ball-and-sticks. Cysteine residues found inside of RLI construct colored in green, others colored in red. The first three cysteines form a single metal-binding site with the final cysteine residue. The other four form the second metal-binding site.
