# Supplementary material for: Analysis of two domains with novel RNA-processing activities throws light on the complex evolution of ribosomal RNA biogenesis
Source: Front Genet. 2014 Dec 23;5:424. doi: 10.3389/fgene.2014.00424 (PMC4275035; doi:10.3389/fgene.2014.00424)
Supplement: Supplementary file 1 [file DataSheet1.ZIP › Supplemental_Material.html]

 

```
Supplementary Material

Analysis of two domains with novel RNA-processing activities throws light on the complex evolution of ribosomal RNA biogenesis
A. Max Burroughs and L Aravind
National Center for Biotechnology Information, National Library of Medicine, National Institutes of Health


Abstract:

Ribosomal biogenesis has been extensively investigated, especially to identify the elusive nucleases and cofactors involved in
the complex rRNA processing events in eukaryotes. Large-scale screens in yeast identified two biochemically uncharacterized
proteins, TSR3 and TSR4, as being key players required for rRNA maturation. Using multiple computational approaches we
identify the conserved domains comprising these proteins and establish sequence and structural features providing novel
insights regarding their roles. TSR3 is unified with the DTW domain into a novel superfamily of predicted RNase domains, with
the archaeo-eukaryotic TSR3 proteins processing the 20S intermediate to yield the 18S rRNA and the bacterial versions
potentially processing tRNA. TSR4, its other eukaryotic homologs PDCD2/rp-8, PDCD2L, Zfrp8, and trus, the predominantly
bacterial DUF1963 proteins, and other uncharacterized proteins are unified into a new domain superfamily, which arose from an
ancient duplication event of a strand-swapped, dimer-forming all-beta unit. We identify conserved features mediating protein-
protein interactions and propose a potential chaperone-like function for it. While contextual evidence supports a conserved
role in ribosome biogenesis for the eukaryotic TSR4-related proteins, there is no evidence for such a role for the bacterial
versions. Whereas TSR3-related proteins can be traced to the last universal common ancestor with a well-supported archaeo-
eukaryotic branch, TSR4-related proteins of eukaryotes are derived from within the bacterial radiation of this superfamily, with
archaea entirely lacking them. This provides evidence for "systems admixture", which followed the early endosymbiotic event,
playing a key role in the emergence of the uniquely eukaryotic ribosome biogenesis process.

Table of Contents:

I. Sequences, domain architectures, and phyletic distributions for domain families
        A. TDD superfamily
                1. TSR3 family
                2. pc1599 family
                3. DTWD2 family
                4. DTWD1 family
                5. AT1G03687 family
        B. TYPP superfamily
                1. YwqG family
                2. PDCD2L family
                3. PDCD2 family
                4. prok.family 2
                5. prok.family 3
                6. prok.family 4
II. Supplementary alignments
        A. Inactive 3�->5� exonuclease domain of the RNase H fold
		B. Individual TDD family alignments
				1. TSR3 family
				2. pc1599 family
				3. DTWD2 family
				4. DTWD1 family
				5. AT1G03687 family
III. Conserved gene neighborhoods
        A. TDD superfamily
                1. TSR3 family ribosomal superoperon associations
                2. DTWD2 family tRNA-associating neighborhoods
        B. TYPP superfamily
                1. YwqG family multiple copy neighborhoods
IV. Alignment figure abbreviations
		A. TDD superfamily
		B. TYPP superfamily

V. Supplemental Figure of RLI construct mapped on 4Fe-4S dicluster domain

Contents:

I. Sequences, domain architectures, and phyletic distributions for domain families

Gene names, gi numbers, phyletic distributions, and domain architectures are provided in a list ordered by following columns:
        gi number, gene name, length of protein, taxonomic lineage, organism name, and refseq definition line for protein.
        Proteins are grouped into shared domain architectures provided at the top of each group following the "#;".

A. TDD superfamily

1. TSR3 family

#;RGG repeats+TDD+negatively-charged regions
303271805       MICPUCDRAFT_50839               367     eukaryota>viridiplantae>chlorophyta                     Micromonas pusilla CCMP1545                                                     predicted protein [Micromonas pusilla CCMP1545].
302836075       VOLCADRAFT_32707                163     eukaryota>viridiplantae>chlorophyta                     Volvox carteri f. nagariensis                                                   hypothetical protein VOLCADRAFT_32707, partial [Volvox carteri f. nagariensis].
308810282       Ot12g03040                      324     eukaryota>viridiplantae>chlorophyta                     Ostreococcus tauri                                                              RNase L inhibitor protein-related (ISS) [Ostreococcus tauri].
159466868       CHLREDRAFT_100576               165     eukaryota>viridiplantae>chlorophyta                     Chlamydomonas reinhardtii                                                       predicted protein, partial [Chlamydomonas reinhardtii].
145352768       OSTLU_7755                      183     eukaryota>viridiplantae>chlorophyta                     Ostreococcus lucimarinus CCE9901                                                predicted protein, partial [Ostreococcus lucimarinus CCE9901].
384253417       COCSUDRAFT_11883                202     eukaryota>viridiplantae>chlorophyta                     Coccomyxa subellipsoidea C-169                                                  DUF367-domain-containing protein, partial [Coccomyxa subellipsoidea C-169].
424513195       Bathy09g04650                   274     eukaryota>viridiplantae>chlorophyta                     Bathycoccus prasinos                                                            predicted protein [Bathycoccus prasinos].
307108165       CHLNCDRAFT_35154                339     eukaryota>viridiplantae>chlorophyta                     Chlorella variabilis                                                            hypothetical protein CHLNCDRAFT_35154 [Chlorella variabilis].
255071489       MICPUN_72212                    221     eukaryota>viridiplantae>chlorophyta                     Micromonas sp. RCC299                                                           predicted protein, partial [Micromonas sp. RCC299].
224081192       POPTRDRAFT_715952               213     eukaryota>viridiplantae                                 Populus trichocarpa                                                             predicted protein [Populus trichocarpa].
302793951       SELMODRAFT_35308                209     eukaryota>viridiplantae                                 Selaginella moellendorffii                                                      hypothetical protein SELMODRAFT_35308, partial [Selaginella moellendorffii].
388493408       -                               233     eukaryota>viridiplantae                                 Lotus japonicus                                                                 unknown [Lotus japonicus].
474069756       TRIUR3_02670                    235     eukaryota>viridiplantae                                 Triticum urartu                                                                 hypothetical protein TRIUR3_02670 [Triticum urartu].
470138185       LOC101290975                    237     eukaryota>viridiplantae                                 Fragaria vesca subsp. vesca                                                     PREDICTED: ribosome biogenesis protein TSR3-like [Fragaria vesca subsp. vesca].
108706648       -                               243     eukaryota>viridiplantae                                 Oryza sativa Japonica Group                                                     Possible metal-binding domain in RNase L inhibitor, RLI family protein, expressed [Oryza sativa Japonica Group].
413956715       ZEAMMB73_487834                 142     eukaryota>viridiplantae                                 Zea mays                                                                        hypothetical protein ZEAMMB73_487834 [Zea mays].
15238053        AT5G10070                       264     eukaryota>viridiplantae                                 Arabidopsis thaliana                                                            RNase L inhibitor protein-like protein [Arabidopsis thaliana].
449509094       LOC101227284                    265     eukaryota>viridiplantae                                 Cucumis sativus                                                                 PREDICTED: ribosome biogenesis protein TSR3 homolog [Cucumis sativus].
449462828       LOC101207685                    265     eukaryota>viridiplantae                                 Cucumis sativus                                                                 PREDICTED: ribosome biogenesis protein TSR3 homolog [Cucumis sativus].
42573329        AT5G10070                       266     eukaryota>viridiplantae                                 Arabidopsis thaliana                                                            RNase L inhibitor protein-like protein [Arabidopsis thaliana].
217426810       -                               267     eukaryota>viridiplantae                                 Arabidopsis arenosa                                                             AT5G10070-like protein [Arabidopsis arenosa].
460388121       LOC101268185                    268     eukaryota>viridiplantae                                 Solanum lycopersicum                                                            PREDICTED: ribosome biogenesis protein TSR3 homolog [Solanum lycopersicum].
482557161       CARUB_v10001716mg               269     eukaryota>viridiplantae                                 Capsella rubella                                                                hypothetical protein CARUB_v10001716mg [Capsella rubella].
168043425       PHYPADRAFT_17562                208     eukaryota>viridiplantae                                 Physcomitrella patens subsp. patens                                             predicted protein, partial [Physcomitrella patens subsp. patens].
297811105       ARALYDRAFT_487833               270     eukaryota>viridiplantae                                 Arabidopsis lyrata subsp. lyrata                                                hypothetical protein ARALYDRAFT_487833 [Arabidopsis lyrata subsp. lyrata].
255645341       -                               270     eukaryota>viridiplantae                                 Glycine max                                                                     unknown [Glycine max].
413956714       ZEAMMB73_487834                 271     eukaryota>viridiplantae                                 Zea mays                                                                        hypothetical protein ZEAMMB73_487834 [Zea mays].
357113618       LOC100839443                    271     eukaryota>viridiplantae                                 Brachypodium distachyon                                                         PREDICTED: probable ribosome biogenesis protein C16orf42 homolog [Brachypodium distachyon].
356512726       LOC100801900                    271     eukaryota>viridiplantae                                 Glycine max                                                                     PREDICTED: probable ribosome biogenesis protein C16orf42 homolog [Glycine max].
226532630       LOC100278811                    271     eukaryota>viridiplantae                                 Zea mays                                                                        uncharacterized protein LOC100278811 [Zea mays].
195612820       -                               271     eukaryota>viridiplantae                                 Zea mays                                                                        hypothetical protein [Zea mays].
194704974       -                               271     eukaryota>viridiplantae                                 Zea mays                                                                        unknown [Zea mays].
502140064       LOC101496229                    272     eukaryota>viridiplantae                                 Cicer arietinum                                                                 PREDICTED: ribosome biogenesis protein TSR3 homolog [Cicer arietinum].
223947055       -                               272     eukaryota>viridiplantae                                 Zea mays                                                                        unknown [Zea mays].
115451311       Os03g0195200                    272     eukaryota>viridiplantae                                 Oryza sativa Japonica Group                                                     Os03g0195200 [Oryza sativa Japonica Group].
357519583       MTR_8g091510                    273     eukaryota>viridiplantae                                 Medicago truncatula                                                             Putative metal-binding domain in RNase L inhibitor RLI family protein [Medicago truncatula].
302787463       SELMODRAFT_36531                205     eukaryota>viridiplantae                                 Selaginella moellendorffii                                                      hypothetical protein SELMODRAFT_36531, partial [Selaginella moellendorffii].
242036621       SORBIDRAFT_01g044120            274     eukaryota>viridiplantae                                 Sorghum bicolor                                                                 hypothetical protein SORBIDRAFT_01g044120 [Sorghum bicolor].
294463865       -                               283     eukaryota>viridiplantae                                 Picea sitchensis                                                                unknown [Picea sitchensis].
255576192       RCOM_0985520                    285     eukaryota>viridiplantae                                 Ricinus communis                                                                conserved hypothetical protein [Ricinus communis].
359480842       LOC100247485                    302     eukaryota>viridiplantae                                 Vitis vinifera                                                                  PREDICTED: probable ribosome biogenesis protein C16orf42 homolog [Vitis vinifera].
475542117       F775_30268                      313     eukaryota>viridiplantae                                 Aegilops tauschii                                                               hypothetical protein F775_30268 [Aegilops tauschii].
72384473        80A08_4                         449     eukaryota>viridiplantae                                 Brassica rapa subsp. pekinensis                                                 80A08_4 [Brassica rapa subsp. pekinensis].
296082467       VIT_00022189001                 326     eukaryota>viridiplantae                                 Vitis vinifera                                                                  unnamed protein product, partial [Vitis vinifera].
24414268        OSJNBa0064E16.8                 327     eukaryota>viridiplantae                                 Oryza sativa Japonica Group                                                     Unknown protein [Oryza sativa Japonica Group].
356525511       LOC100777863                    270     eukaryota>viridiplantae                                 Glycine max                                                                     PREDICTED: probable ribosome biogenesis protein C16orf42 homolog [Glycine max].
462401376       PRUPE_ppa010103mg               264     eukaryota>viridiplantae                                 Prunus persica                                                                  hypothetical protein PRUPE_ppa010103mg [Prunus persica].
348664932       PHYSODRAFT_362612               356     eukaryota>stramenopiles                                 Phytophthora sojae                                                              hypothetical protein PHYSODRAFT_362612 [Phytophthora sojae].
301090267       PITG_20274                      340     eukaryota>stramenopiles                                 Phytophthora infestans T30-4                                                    conserved hypothetical protein [Phytophthora infestans T30-4].
298712806       Esi_0047_0071                   334     eukaryota>stramenopiles                                 Ectocarpus siliculosus                                                          conserved unknown protein [Ectocarpus siliculosus].
219112685       PHATRDRAFT_9706                 169     eukaryota>stramenopiles                                 Phaeodactylum tricornutum CCAP 1055/1                                           predicted protein, partial [Phaeodactylum tricornutum CCAP 1055/1].
223995503       THAPSDRAFT_32295                170     eukaryota>stramenopiles                                 Thalassiosira pseudonana CCMP1335                                               predicted protein, partial [Thalassiosira pseudonana CCMP1335].
323452020       AURANDRAFT_7323                 176     eukaryota>stramenopiles                                 Aureococcus anophagefferens                                                     hypothetical protein AURANDRAFT_7323, partial [Aureococcus anophagefferens].
325190886       ALNC14_115150                   369     eukaryota>stramenopiles                                 Albugo laibachii Nc14                                                           conserved hypothetical protein [Albugo laibachii Nc14].
397648293       THAOC_00042                     284     eukaryota>stramenopiles                                 Thalassiosira oceanica                                                          hypothetical protein THAOC_00042 [Thalassiosira oceanica].
300122558       GSBLH_T00003052001              238     eukaryota>stramenopiles                                 Blastocystis hominis                                                            unnamed protein product [Blastocystis hominis].
452825092       Gasu_08340                      275     eukaryota>rhodophyta                                    Galdieria sulphuraria                                                           hypothetical protein Gasu_08340 [Galdieria sulphuraria].
449015709       CYME_CMD016C                    222     eukaryota>rhodophyta                                    Cyanidioschyzon merolae strain 10D                                              hypothetical protein, conserved [Cyanidioschyzon merolae strain 10D].
154417918       TVAG_172490                     290     eukaryota>parabasalia                                   Trichomonas vaginalis G3                                                        Possible metal-binding domain in RNase L inhibitor, RLI family protein [Trichomonas vaginalis G3].
196016474       TRIADDRAFT_62139                284     eukaryota>metazoa>placozoa                              Trichoplax adhaerens                                                            hypothetical protein TRIADDRAFT_62139 [Trichoplax adhaerens].
402587265       WUBG_07889                      201     eukaryota>metazoa>nematoda                              Wuchereria bancrofti                                                            hypothetical protein WUBG_07889 [Wuchereria bancrofti].
341892712       CAEBREN_17378                   260     eukaryota>metazoa>nematoda                              Caenorhabditis brenneri                                                         hypothetical protein CAEBREN_17378 [Caenorhabditis brenneri].
170587911       Bm1_36350                       234     eukaryota>metazoa>nematoda                              Brugia malayi                                                                   Possible metal-binding domain in RNase L inhibitor, RLI family protein [Brugia malayi].
312070418       LOAG_02552                      233     eukaryota>metazoa>nematoda                              Loa loa                                                                         hypothetical protein LOAG_02552 [Loa loa].
25148466        CELE_F52C12.2                   261     eukaryota>metazoa>nematoda                              Caenorhabditis elegans                                                          Protein F52C12.2 [Caenorhabditis elegans].
308462609       CRE_02617                       310     eukaryota>metazoa>nematoda                              Caenorhabditis remanei                                                          hypothetical protein CRE_02617 [Caenorhabditis remanei].
195157644       Dper_GL12540                    263     eukaryota>metazoa>hexapoda                              Drosophila persimilis                                                           GL12540 [Drosophila persimilis].
383864286       LOC100880883                    264     eukaryota>metazoa>hexapoda                              Megachile rotundata                                                             PREDICTED: probable ribosome biogenesis protein C16orf42 homolog [Megachile rotundata].
195110261       Dmoj_GI22926                    264     eukaryota>metazoa>hexapoda                              Drosophila mojavensis                                                           GI22926 [Drosophila mojavensis].
322802279       SINV_06642                      265     eukaryota>metazoa>hexapoda                              Solenopsis invicta                                                              hypothetical protein SINV_06642, partial [Solenopsis invicta].
307172448       EAG_14988                       265     eukaryota>metazoa>hexapoda                              Camponotus floridanus                                                           UPF0293 protein C16orf42 [Camponotus floridanus].
195390079       Dvir_GJ23209                    265     eukaryota>metazoa>hexapoda                              Drosophila virilis                                                              GJ23209 [Drosophila virilis].
125778282       Dpse_GA18120                    266     eukaryota>metazoa>hexapoda                              Drosophila pseudoobscura pseudoobscura                                          GA18120 [Drosophila pseudoobscura pseudoobscura].
328788025       LOC552789                       267     eukaryota>metazoa>hexapoda                              Apis mellifera                                                                  PREDICTED: UPF0293 protein C16orf42-like [Apis mellifera].
239791543       ACYPI000703                     247     eukaryota>metazoa>hexapoda                              Acyrthosiphon pisum                                                             ACYPI000703 [Acyrthosiphon pisum].
307206157       EAI_10025                       268     eukaryota>metazoa>hexapoda                              Harpegnathos saltator                                                           UPF0293 protein C16orf42-like protein [Harpegnathos saltator].
389609717       -                               263     eukaryota>metazoa>hexapoda                              Papilio xuthus                                                                  similar to CG4338 [Papilio xuthus].
157125951       AaeL_AAEL010333                 269     eukaryota>metazoa>hexapoda                              Aedes aegypti                                                                   hypothetical protein AaeL_AAEL010333 [Aedes aegypti].
332031297       G5I_00383                       270     eukaryota>metazoa>hexapoda                              Acromyrmex echinatior                                                           UPF0293 protein C16orf42-like protein [Acromyrmex echinatior].
498934027       LOC101448381                    271     eukaryota>metazoa>hexapoda                              Ceratitis capitata                                                              PREDICTED: ribosome biogenesis protein TSR3 homolog [Ceratitis capitata].
195328791       Dsec_GM24214                    272     eukaryota>metazoa>hexapoda                              Drosophila sechellia                                                            GM24214 [Drosophila sechellia].
195501371       Dyak_GE24288                    273     eukaryota>metazoa>hexapoda                              Drosophila yakuba                                                               GE24288 [Drosophila yakuba].
194900966       Dere_GG16907                    274     eukaryota>metazoa>hexapoda                              Drosophila erecta                                                               GG16907 [Drosophila erecta].
24647066        Dmel_CG4338                     274     eukaryota>metazoa>hexapoda                              Drosophila melanogaster                                                         CG4338 [Drosophila melanogaster].
158296670       AgaP_AGAP008424                 207     eukaryota>metazoa>hexapoda                              Anopheles gambiae str. PEST                                                     AGAP008424-PA, partial [Anopheles gambiae str. PEST].
340717903       LOC100649262                    277     eukaryota>metazoa>hexapoda                              Bombus terrestris                                                               PREDICTED: probable ribosome biogenesis protein C16orf42-like [Bombus terrestris].
350400300       LOC100748715                    278     eukaryota>metazoa>hexapoda                              Bombus impatiens                                                                PREDICTED: probable ribosome biogenesis protein C16orf42-like [Bombus impatiens].
312374465       AND_15885                       235     eukaryota>metazoa>hexapoda                              Anopheles darlingi                                                              hypothetical protein AND_15885 [Anopheles darlingi].
332028977       G5I_02299                       235     eukaryota>metazoa>hexapoda                              Acromyrmex echinatior                                                           UPF0293 protein C16orf42 [Acromyrmex echinatior].
170041678       CpipJ_CPIJ007052                260     eukaryota>metazoa>hexapoda                              Culex quinquefasciatus                                                          conserved hypothetical protein [Culex quinquefasciatus].
242015816       Phum_PHUM390100                 387     eukaryota>metazoa>hexapoda                              Pediculus humanus corporis                                                      conserved hypothetical protein [Pediculus humanus corporis].
389614886       -                               250     eukaryota>metazoa>hexapoda                              Papilio polytes                                                                 simila to CG4338, partial [Papilio polytes].
193608333       LOC100159320                    247     eukaryota>metazoa>hexapoda                              Acyrthosiphon pisum                                                             PREDICTED: UPF0293 protein C16orf42 homolog [Acyrthosiphon pisum].
345491132       LOC100679095                    175     eukaryota>metazoa>hexapoda                              Nasonia vitripennis                                                             PREDICTED: probable ribosome biogenesis protein C16orf42-like, partial [Nasonia vitripennis].
194767675       Dana_GF11442                    268     eukaryota>metazoa>hexapoda                              Drosophila ananassae                                                            GF11442 [Drosophila ananassae].
357617627       KGM_13042                       249     eukaryota>metazoa>hexapoda                              Danaus plexippus                                                                hypothetical protein KGM_13042 [Danaus plexippus].
195451177       Dwil_GK13794                    228     eukaryota>metazoa>hexapoda                              Drosophila willistoni                                                           GK13794 [Drosophila willistoni].
270003838       TcasGA2_TC003119                214     eukaryota>metazoa>hexapoda                              Tribolium castaneum                                                             hypothetical protein TcasGA2_TC003119 [Tribolium castaneum].
322786607       SINV_09910                      167     eukaryota>metazoa>hexapoda                              Solenopsis invicta                                                              hypothetical protein SINV_09910, partial [Solenopsis invicta].
478252712       YQE_10248                       226     eukaryota>metazoa>hexapoda                              Dendroctonus ponderosae                                                         hypothetical protein YQE_10248, partial [Dendroctonus ponderosae].
195036156       Dgri_GH18855                    267     eukaryota>metazoa>hexapoda                              Drosophila grimshawi                                                            GH18855 [Drosophila grimshawi].
390348262       LOC586848                       400     eukaryota>metazoa>echinodermata                         Strongylocentrotus purpuratus                                                   PREDICTED: probable ribosome biogenesis protein C16orf42 homolog [Strongylocentrotus purpuratus].
390367731       LOC590107                       247     eukaryota>metazoa>echinodermata                         Strongylocentrotus purpuratus                                                   PREDICTED: probable ribosome biogenesis protein C16orf42 homolog, partial [Strongylocentrotus purpuratus].
321465713       DAPPUDRAFT_306096               269     eukaryota>metazoa>crustacea                             Daphnia pulex                                                                   hypothetical protein DAPPUDRAFT_306096 [Daphnia pulex].
321452067       DAPPUDRAFT_67042                181     eukaryota>metazoa>crustacea                             Daphnia pulex                                                                   hypothetical protein DAPPUDRAFT_67042, partial [Daphnia pulex].
156388863       NEMVEDRAFT_v1g100326            176     eukaryota>metazoa>cnidaria                              Nematostella vectensis                                                          predicted protein, partial [Nematostella vectensis].
449686566       LOC100199319                    281     eukaryota>metazoa>cnidaria                              Hydra magnipapillata                                                            PREDICTED: ribosome biogenesis protein TSR3 homolog, partial [Hydra magnipapillata].
499023507       LOC101464436                    296     eukaryota>metazoa>chordata>vertebrata>actinopterygii    Maylandia zebra                                                                 PREDICTED: ribosome biogenesis protein TSR3 homolog [Maylandia zebra].
432924338       LOC101166503                    287     eukaryota>metazoa>chordata>vertebrata>actinopterygii    Oryzias latipes                                                                 PREDICTED: ribosome biogenesis protein TSR3 homolog [Oryzias latipes].
47225997        GSTEN:00024074:G:001            247     eukaryota>metazoa>chordata>vertebrata>actinopterygii    Tetraodon nigroviridis                                                          unnamed protein product, partial [Tetraodon nigroviridis].
348502529       LOC100696584                    295     eukaryota>metazoa>chordata>vertebrata>actinopterygii    Oreochromis niloticus                                                           PREDICTED: ribosome biogenesis protein TSR3 homolog [Oreochromis niloticus].
410896027       LOC101072092                    279     eukaryota>metazoa>chordata>vertebrata>actinopterygii    Takifugu rubripes                                                               PREDICTED: ribosome biogenesis protein TSR3 homolog [Takifugu rubripes].
317419259       DLA_Ib01380                     307     eukaryota>metazoa>chordata>vertebrata>actinopterygii    Dicentrarchus labrax                                                            Uncharacterized protein [Dicentrarchus labrax].
50539800        tsr3                            297     eukaryota>metazoa>chordata>vertebrata>actinopterygii    Danio rerio                                                                     TSR3, 20S rRNA accumulation, homolog [Danio rerio].
225706146       YO006                           283     eukaryota>metazoa>chordata>vertebrata>actinopterygii    Osmerus mordax                                                                  UPF0293 protein YOR006C [Osmerus mordax].
488549974       TSR3                            191     eukaryota>metazoa>chordata>vertebrata                   Dasypus novemcinctus                                                            PREDICTED: ribosome biogenesis protein TSR3 homolog isoform 2 [Dasypus novemcinctus].
344248302       I79_007920                      265     eukaryota>metazoa>chordata>vertebrata                   Cricetulus griseus                                                              UPF0293 protein C16orf42-like [Cricetulus griseus].
441659678       TSR3                            199     eukaryota>metazoa>chordata>vertebrata                   Nomascus leucogenys                                                             PREDICTED: ribosome biogenesis protein TSR3 homolog [Nomascus leucogenys].
301769659       LOC100465587                    274     eukaryota>metazoa>chordata>vertebrata                   Ailuropoda melanoleuca                                                          PREDICTED: UPF0293 protein C16orf42-like [Ailuropoda melanoleuca].
449278972       A306_04792                      260     eukaryota>metazoa>chordata>vertebrata                   Columba livia                                                                   UPF0293 protein C16orf42 like protein, partial [Columba livia].
24416579        0610007P22Rik                   201     eukaryota>metazoa>chordata>vertebrata                   Mus musculus                                                                    0610007P22Rik protein [Mus musculus].
432102559       MDA_GLEAN10007790               254     eukaryota>metazoa>chordata>vertebrata                   Myotis davidii                                                                  hypothetical protein MDA_GLEAN10007790 [Myotis davidii].
403273226       TSR3                            338     eukaryota>metazoa>chordata>vertebrata                   Saimiri boliviensis boliviensis                                                 PREDICTED: probable ribosome biogenesis protein C16orf42 homolog [Saimiri boliviensis boliviensis].
332844988       TSR3                            437     eukaryota>metazoa>chordata>vertebrata                   Pan troglodytes                                                                 PREDICTED: ribosome biogenesis protein TSR3 homolog [Pan troglodytes].
148669248       mCG_17635                       165     eukaryota>metazoa>chordata>vertebrata                   Mus musculus                                                                    RIKEN cDNA 0610007P22, isoform CRA_b, partial [Mus musculus].
148669247       mCG_17635                       350     eukaryota>metazoa>chordata>vertebrata                   Mus musculus                                                                    RIKEN cDNA 0610007P22, isoform CRA_a, partial [Mus musculus].
444727284       TREES_T100011753                239     eukaryota>metazoa>chordata>vertebrata                   Tupaia chinensis                                                                hypothetical protein TREES_T100011753 [Tupaia chinensis].
224070656       TSR3                            291     eukaryota>metazoa>chordata>vertebrata                   Taeniopygia guttata                                                             PREDICTED: ribosome biogenesis protein TSR3 homolog [Taeniopygia guttata].
327287192       LOC100561137                    294     eukaryota>metazoa>chordata>vertebrata                   Anolis carolinensis                                                             PREDICTED: UPF0293 protein C16orf42-like [Anolis carolinensis].
426254981       TSR3                            235     eukaryota>metazoa>chordata>vertebrata                   Ovis aries                                                                      PREDICTED: LOW QUALITY PROTEIN: ribosome biogenesis protein TSR3 homolog, partial [Ovis aries].
334333567       LOC100010388                    331     eukaryota>metazoa>chordata>vertebrata                   Monodelphis domestica                                                           PREDICTED: UPF0293 protein C16orf42 homolog [Monodelphis domestica].
395515774       TSR3                            331     eukaryota>metazoa>chordata>vertebrata                   Sarcophilus harrisii                                                            PREDICTED: probable ribosome biogenesis protein C16orf42 homolog [Sarcophilus harrisii].
148669249       mCG_17635                       233     eukaryota>metazoa>chordata>vertebrata                   Mus musculus                                                                    RIKEN cDNA 0610007P22, isoform CRA_c, partial [Mus musculus].
410985573       TSR3                            231     eukaryota>metazoa>chordata>vertebrata                   Felis catus                                                                     PREDICTED: ribosome biogenesis protein TSR3 homolog, partial [Felis catus].
345315354       LOC100087005                    228     eukaryota>metazoa>chordata>vertebrata                   Ornithorhynchus anatinus                                                        PREDICTED: probable ribosome biogenesis protein C16orf42-like [Ornithorhynchus anatinus].
351711175       GW7_12950                       298     eukaryota>metazoa>chordata>vertebrata                   Heterocephalus glaber                                                           hypothetical protein GW7_12950 [Heterocephalus glaber].
488549972       TSR3                            301     eukaryota>metazoa>chordata>vertebrata                   Dasypus novemcinctus                                                            PREDICTED: ribosome biogenesis protein TSR3 homolog isoform 1 [Dasypus novemcinctus].
355709826       EGK_12336                       275     eukaryota>metazoa>chordata>vertebrata                   Macaca mulatta                                                                  hypothetical protein EGK_12336, partial [Macaca mulatta].
470595283       TSR3                            309     eukaryota>metazoa>chordata>vertebrata                   Tursiops truncatus                                                              PREDICTED: ribosome biogenesis protein TSR3 homolog [Tursiops truncatus].
149052099       rCG_32779                       324     eukaryota>metazoa>chordata>vertebrata                   Rattus norvegicus                                                               similar to RIKEN cDNA 0610007P22 (predicted), isoform CRA_b [Rattus norvegicus].
194219397       TSR3                            324     eukaryota>metazoa>chordata>vertebrata                   Equus caballus                                                                  PREDICTED: LOW QUALITY PROTEIN: ribosome biogenesis protein TSR3 homolog [Equus caballus].
466011711       TSR3                            309     eukaryota>metazoa>chordata>vertebrata                   Orcinus orca                                                                    PREDICTED: ribosome biogenesis protein TSR3 homolog [Orcinus orca].
464402212       C14H16ORF42                     311     eukaryota>metazoa>chordata>vertebrata                   Gallus gallus                                                                   ribosome biogenesis protein TSR3 homolog [Gallus gallus].
12832196        -                               323     eukaryota>metazoa>chordata>vertebrata                   Mus musculus                                                                    unnamed protein product [Mus musculus].
478528167       TSR3                            312     eukaryota>metazoa>chordata>vertebrata                   Ceratotherium simum simum                                                       PREDICTED: ribosome biogenesis protein TSR3 homolog [Ceratotherium simum simum].
354478841       LOC100751647                    323     eukaryota>metazoa>chordata>vertebrata                   Cricetulus griseus                                                              PREDICTED: probable ribosome biogenesis protein C16orf42 homolog isoform 1 [Cricetulus griseus].
402907207       TSR3                            312     eukaryota>metazoa>chordata>vertebrata                   Papio anubis                                                                    PREDICTED: probable ribosome biogenesis protein C16orf42 homolog [Papio anubis].
395835711       TSR3                            312     eukaryota>metazoa>chordata>vertebrata                   Otolemur garnettii                                                              PREDICTED: probable ribosome biogenesis protein C16orf42 homolog [Otolemur garnettii].
302564075       C20H16orf42                     312     eukaryota>metazoa>chordata>vertebrata                   Macaca mulatta                                                                  probable ribosome biogenesis protein C16orf42 [Macaca mulatta].
297697724       TSR3                            312     eukaryota>metazoa>chordata>vertebrata                   Pongo abelii                                                                    PREDICTED: probable ribosome biogenesis protein C16orf42 homolog [Pongo abelii].
296219257       C12H16orf42                     312     eukaryota>metazoa>chordata>vertebrata                   Callithrix jacchus                                                              PREDICTED: probable ribosome biogenesis protein C16orf42 [Callithrix jacchus].
426380680       TSR3                            320     eukaryota>metazoa>chordata>vertebrata                   Gorilla gorilla gorilla                                                         PREDICTED: ribosome biogenesis protein TSR3 homolog [Gorilla gorilla gorilla].
348584770       LOC100727620                    319     eukaryota>metazoa>chordata>vertebrata                   Cavia porcellus                                                                 PREDICTED: LOW QUALITY PROTEIN: probable ribosome biogenesis protein C16orf42-like [Cavia porcellus].
57088115        TSR3                            312     eukaryota>metazoa>chordata>vertebrata                   Canis lupus familiaris                                                          PREDICTED: ribosome biogenesis protein TSR3 homolog [Canis lupus familiaris].
431906708       PAL_GLEAN10011745               317     eukaryota>metazoa>chordata>vertebrata                   Pteropus alecto                                                                 hypothetical protein PAL_GLEAN10011745 [Pteropus alecto].
471365328       TSR3                            317     eukaryota>metazoa>chordata>vertebrata                   Trichechus manatus latirostris                                                  PREDICTED: ribosome biogenesis protein TSR3 homolog [Trichechus manatus latirostris].
255003700       Tsr3                            316     eukaryota>metazoa>chordata>vertebrata                   Mus musculus                                                                    ribosome biogenesis protein TSR3 homolog isoform 2 [Mus musculus].
335284824       TSR3                            316     eukaryota>metazoa>chordata>vertebrata                   Sus scrofa                                                                      PREDICTED: ribosome biogenesis protein TSR3 homolog isoform X1 [Sus scrofa].
354478843       LOC100751647                    316     eukaryota>metazoa>chordata>vertebrata                   Cricetulus griseus                                                              PREDICTED: probable ribosome biogenesis protein C16orf42 homolog isoform 2 [Cricetulus griseus].
472367284       TSR3                            316     eukaryota>metazoa>chordata>vertebrata                   Odobenus rosmarus divergens                                                     PREDICTED: ribosome biogenesis protein TSR3 homolog [Odobenus rosmarus divergens].
149642877       TSR3                            315     eukaryota>metazoa>chordata>vertebrata                   Bos taurus                                                                      probable ribosome biogenesis protein C16orf42 homolog [Bos taurus].
47777330        TSR3                            312     eukaryota>metazoa>chordata>vertebrata                   Homo sapiens                                                                    ribosome biogenesis protein TSR3 homolog [Homo sapiens].
344292048       LOC100669412                    314     eukaryota>metazoa>chordata>vertebrata                   Loxodonta africana                                                              PREDICTED: probable ribosome biogenesis protein C16orf42-like [Loxodonta africana].
189441588       LOC549889                       313     eukaryota>metazoa>chordata>vertebrata                   Xenopus (Silurana) tropicalis                                                   LOC549889 protein [Xenopus (Silurana) tropicalis].
157422971       LOC549889                       313     eukaryota>metazoa>chordata>vertebrata                   Xenopus (Silurana) tropicalis                                                   hypothetical protein LOC549889 [Xenopus (Silurana) tropicalis].
62858567        tsr3                            313     eukaryota>metazoa>chordata>vertebrata                   Xenopus (Silurana) tropicalis                                                   uncharacterized protein LOC549889 [Xenopus (Silurana) tropicalis].
260824251       BRAFLDRAFT_68128                282     eukaryota>metazoa>chordata                              Branchiostoma floridae                                                          hypothetical protein BRAFLDRAFT_68128 [Branchiostoma floridae].
313226566       GSOID_T00009487001              236     eukaryota>metazoa>chordata                              Oikopleura dioica                                                               unnamed protein product [Oikopleura dioica].
198421434       LOC100176322                    265     eukaryota>metazoa>chordata                              Ciona intestinalis                                                              PREDICTED: ribosome biogenesis protein TSR3 homolog [Ciona intestinalis].
313240652       GSOID_T00020839001              236     eukaryota>metazoa>chordata                              Oikopleura dioica                                                               unnamed protein product [Oikopleura dioica].
443719185       CAPTEDRAFT_115709               184     eukaryota>metazoa>annelida                              Capitella teleta                                                                hypothetical protein CAPTEDRAFT_115709, partial [Capitella teleta].
256078661       Smp_042650                      372     eukaryota>metazoa                                       Schistosoma mansoni                                                             hypothetical protein [Schistosoma mansoni].
340371999       LOC100637117                    247     eukaryota>metazoa                                       Amphimedon queenslandica                                                        PREDICTED: probable ribosome biogenesis protein C16orf42 homolog [Amphimedon queenslandica].
391340873       LOC100903897                    273     eukaryota>metazoa                                       Metaseiulus occidentalis                                                        PREDICTED: probable ribosome biogenesis protein C16orf42 homolog [Metaseiulus occidentalis].
290979551       NAEGRDRAFT_81227                388     eukaryota>heterolobosea                                 Naegleria gruberi strain NEG-M                                                  DUF367 domain-containing protein [Naegleria gruberi].
485648863       EMIHUDRAFT_56186                167     eukaryota>haptophyceae                                  Emiliania huxleyi CCMP1516                                                      hypothetical protein EMIHUDRAFT_56186, partial [Emiliania huxleyi CCMP1516].
387593489       NEQG_01203                      173     eukaryota>fungi>microsporidia                           Nematocida parisii ERTm3                                                        hypothetical protein NEQG_01203 [Nematocida parisii ERTm3].
85014419        ECU09_1520                      179     eukaryota>fungi>microsporidia                           Encephalitozoon cuniculi GB-M1                                                  hypothetical protein ECU09_1520 [Encephalitozoon cuniculi GB-M1].
303390751       Eint_091170                     172     eukaryota>fungi>microsporidia                           Encephalitozoon intestinalis ATCC 50506                                         hypothetical protein Eint_091170 [Encephalitozoon intestinalis ATCC 50506].
440493274       THOM_1297                       174     eukaryota>fungi>microsporidia                           Trachipleistophora hominis                                                      hypothetical protein THOM_1297 [Trachipleistophora hominis].
47157019        -                               180     eukaryota>fungi>microsporidia                           Antonospora locustae                                                            hypothetical protein [Antonospora locustae].
429964761       VCUG_01785                      180     eukaryota>fungi>microsporidia                           Vavraia culicis subsp. floridensis                                              hypothetical protein VCUG_01785 [Vavraia culicis subsp. floridensis].
300702317       NCER_102048                     175     eukaryota>fungi>microsporidia                           Nosema ceranae BRL01                                                            hypothetical protein NCER_102048 [Nosema ceranae BRL01].
401828553       EHEL_091140                     172     eukaryota>fungi>microsporidia                           Encephalitozoon hellem ATCC 50504                                               hypothetical protein EHEL_091140 [Encephalitozoon hellem ATCC 50504].
378755074       NERG_01547                      173     eukaryota>fungi>microsporidia                           Nematocida sp. 1 ERTm2                                                          hypothetical protein NERG_01547 [Nematocida sp. 1 ERTm2].
429961755       VICG_01673                      174     eukaryota>fungi>microsporidia                           Vittaforma corneae ATCC 50505                                                   hypothetical protein VICG_01673 [Vittaforma corneae ATCC 50505].
402465805       EDEG_00451                      197     eukaryota>fungi>microsporidia                           Edhazardia aedis USNM 41457                                                     hypothetical protein EDEG_00451 [Edhazardia aedis USNM 41457].
396082096       EROM_090920                     172     eukaryota>fungi>microsporidia                           Encephalitozoon romaleae SJ-2008                                                hypothetical protein EROM_090920 [Encephalitozoon romaleae SJ-2008].
269861445       EBI_27323                       174     eukaryota>fungi>microsporidia                           Enterocytozoon bieneusi H348                                                    RNase L inhibitor protein [Enterocytozoon bieneusi H348].
328772902       BATDEDRAFT_21183                270     eukaryota>fungi>chytridiomycota                         Batrachochytrium dendrobatidis JAM81                                            hypothetical protein BATDEDRAFT_21183 [Batrachochytrium dendrobatidis JAM81].
328772323       BATDEDRAFT_86599                290     eukaryota>fungi>chytridiomycota                         Batrachochytrium dendrobatidis JAM81                                            hypothetical protein BATDEDRAFT_86599 [Batrachochytrium dendrobatidis JAM81].
472580836       RHTO_05861                      306     eukaryota>fungi>basidiomycota                           Rhodosporidium toruloides NP11                                                  DUF367 and RNase L inhibitor RLI, possible metal-binding domain protein [Rhodosporidium toruloides NP11].
170087962       LACBIDRAFT_244871               200     eukaryota>fungi>basidiomycota                           Laccaria bicolor S238N-H82                                                      predicted protein, partial [Laccaria bicolor S238N-H82].
392573420       TREMEDRAFT_34864                201     eukaryota>fungi>basidiomycota                           Tremella mesenterica DSM 1558                                                   hypothetical protein TREMEDRAFT_34864, partial [Tremella mesenterica DSM 1558].
336388378       SERLADRAFT_359520               270     eukaryota>fungi>basidiomycota                           Serpula lacrymans var. lacrymans S7.9                                           hypothetical protein SERLADRAFT_359520 [Serpula lacrymans var. lacrymans S7.9].
164656515       MGL_3420                        306     eukaryota>fungi>basidiomycota                           Malassezia globosa CBS 7966                                                     hypothetical protein MGL_3420 [Malassezia globosa CBS 7966].
390599664       PUNSTDRAFT_134235               261     eukaryota>fungi>basidiomycota                           Punctularia strigosozonata HHB-11173 SS5                                        DUF367-domain-containing protein [Punctularia strigosozonata HHB-11173 SS5].
449544273       CERSUDRAFT_138933               220     eukaryota>fungi>basidiomycota                           Ceriporiopsis subvermispora B                                                   hypothetical protein CERSUDRAFT_138933 [Ceriporiopsis subvermispora B].
402219499       DACRYDRAFT_81957                251     eukaryota>fungi>basidiomycota                           Dacryopinax sp. DJM-731 SS1                                                     DUF367-domain-containing protein [Dacryopinax sp. DJM-731 SS1].
403160068       PGTG_02648                      300     eukaryota>fungi>basidiomycota                           Puccinia graminis f. sp. tritici CRL 75-36-700-3                                hypothetical protein PGTG_02648 [Puccinia graminis f. sp. tritici CRL 75-36-700-3].
409048462       PHACADRAFT_90952                203     eukaryota>fungi>basidiomycota                           Phanerochaete carnosa HHB-10118-sp                                              hypothetical protein PHACADRAFT_90952 [Phanerochaete carnosa HHB-10118-sp].
392589416       CONPUDRAFT_145844               362     eukaryota>fungi>basidiomycota                           Coniophora puteana RWD-64-598 SS2                                               DUF367-domain-containing protein [Coniophora puteana RWD-64-598 SS2].
328851533       MELLADRAFT_39545                209     eukaryota>fungi>basidiomycota                           Melampsora larici-populina 98AG31                                               hypothetical protein MELLADRAFT_39545 [Melampsora larici-populina 98AG31].
443898348       PANT_18c00014                   356     eukaryota>fungi>basidiomycota                           Pseudozyma antarctica T-34                                                      uncharacterized conserved protein [Pseudozyma antarctica T-34].
388855614       UHOR_06426                      338     eukaryota>fungi>basidiomycota                           Ustilago hordei                                                                 uncharacterized protein UHOR_06426 [Ustilago hordei].
388582400       WALSEDRAFT_12058                177     eukaryota>fungi>basidiomycota                           Wallemia sebi CBS 633.66                                                        DUF367-domain-containing protein, partial [Wallemia sebi CBS 633.66].
299742664       CC1G_08596                      296     eukaryota>fungi>basidiomycota                           Coprinopsis cinerea okayama7#130                                                DUF367 family protein [Coprinopsis cinerea okayama7#130].
302678003       SCHCODRAFT_59936                233     eukaryota>fungi>basidiomycota                           Schizophyllum commune H4-8                                                      hypothetical protein SCHCODRAFT_59936 [Schizophyllum commune H4-8].
353235505       PIIN_01346                      281     eukaryota>fungi>basidiomycota                           Piriformospora indica DSM 11827                                                 hypothetical protein PIIN_01346 [Piriformospora indica DSM 11827].
401883890       A1Q1_02990                      333     eukaryota>fungi>basidiomycota                           Trichosporon asahii var. asahii CBS 2479                                        hypothetical protein A1Q1_02990 [Trichosporon asahii var. asahii CBS 2479].
392559528       TRAVEDRAFT_24152                213     eukaryota>fungi>basidiomycota                           Trametes versicolor FP-101664 SS1                                               DUF367-domain-containing protein [Trametes versicolor FP-101664 SS1].
405122990       CNAG_01550                      293     eukaryota>fungi>basidiomycota                           Cryptococcus neoformans var. grubii H99                                         hypothetical protein CNAG_01550 [Cryptococcus neoformans var. grubii H99].
501311084       PHSY_007413                     362     eukaryota>fungi>basidiomycota                           Pseudozyma hubeiensis SY62                                                      hypothetical protein PHSY_007413 [Pseudozyma hubeiensis SY62].
426195128       AGABI2DRAFT_194102              282     eukaryota>fungi>basidiomycota                           Agaricus bisporus var. bisporus H97                                             hypothetical protein AGABI2DRAFT_194102 [Agaricus bisporus var. bisporus H97].
321252973       CGB_C1180W                      291     eukaryota>fungi>basidiomycota                           Cryptococcus gattii WM276                                                       hypothetical protein CGB_C1180W [Cryptococcus gattii WM276].
58265470        CNC06340                        290     eukaryota>fungi>basidiomycota                           Cryptococcus neoformans var. neoformans JEC21                                   hypothetical protein [Cryptococcus neoformans var. neoformans JEC21].
71020401        UM04284.1                       368     eukaryota>fungi>basidiomycota                           Ustilago maydis 521                                                             hypothetical protein UM04284.1 [Ustilago maydis 521].
393220593       FOMMEDRAFT_119592               217     eukaryota>fungi>basidiomycota                           Fomitiporia mediterranea MF3/22                                                 DUF367-domain-containing protein [Fomitiporia mediterranea MF3/22].
242209067       POSPLDRAFT_41450                197     eukaryota>fungi>basidiomycota                           Postia placenta Mad-698-R                                                       predicted protein, partial [Postia placenta Mad-698-R].
465794015       MSY001_1622                     289     eukaryota>fungi>basidiomycota                           Malassezia sympodialis ATCC 42132                                               unnamed protein product [Malassezia sympodialis ATCC 42132].
409076307       AGABI1DRAFT_115592              282     eukaryota>fungi>basidiomycota                           Agaricus bisporus var. burnettii JB137-S8                                       hypothetical protein AGABI1DRAFT_115592 [Agaricus bisporus var. burnettii JB137-S8].
343425323       sr15176                         375     eukaryota>fungi>basidiomycota                           Sporisorium reilianum SRZ2                                                      conserved hypothetical protein [Sporisorium reilianum SRZ2].
393242446       AURDEDRAFT_143587               284     eukaryota>fungi>basidiomycota                           Auricularia delicata TFB-10046 SS5                                              DUF367-domain-containing protein [Auricularia delicata TFB-10046 SS5].
389747506       STEHIDRAFT_93621                286     eukaryota>fungi>basidiomycota                           Stereum hirsutum FP-91666 SS1                                                   DUF367-domain-containing protein [Stereum hirsutum FP-91666 SS1].
451855369       COCSADRAFT_275043               398     eukaryota>fungi>ascomycota                              Bipolaris sorokiniana ND90Pr                                                    hypothetical protein COCSADRAFT_275043 [Bipolaris sorokiniana ND90Pr].
260950983       CLUG_00947                      463     eukaryota>fungi>ascomycota                              Clavispora lusitaniae ATCC 42720                                                hypothetical protein CLUG_00947 [Clavispora lusitaniae ATCC 42720].
425769170       PDIP_72630                      369     eukaryota>fungi>ascomycota                              Penicillium digitatum Pd1                                                       hypothetical protein PDIP_72630 [Penicillium digitatum Pd1].
294659366       DEHA2G04290g                    333     eukaryota>fungi>ascomycota                              Debaryomyces hansenii CBS767                                                    DEHA2G04290p [Debaryomyces hansenii CBS767].
259484812       ANIA_05711                      370     eukaryota>fungi>ascomycota                              Aspergillus nidulans FGSC A4                                                    TPA: RLI and DUF367 domain protein (AFU_orthologue; AFUA_1G06690) [Aspergillus nidulans FGSC A4].
320584111       HPODL_0002                      335     eukaryota>fungi>ascomycota                              Ogataea parapolymorpha DL-1                                                     hypothetical protein HPODL_0002 [Ogataea parapolymorpha DL-1].
378732187       HMPREF1120_06650                398     eukaryota>fungi>ascomycota                              Exophiala dermatitidis NIH/UT8656                                               hypothetical protein HMPREF1120_06650 [Exophiala dermatitidis NIH/UT8656].
254566229       PAS_chr1-4_0115                 287     eukaryota>fungi>ascomycota                              Komagataella pastoris GS115                                                     Putative protein of unknown function [Komagataella pastoris GS115].
19113919        SPAC1F3.04c                     288     eukaryota>fungi>ascomycota                              Schizosaccharomyces pombe 972h-                                                 SSU-rRNA maturation protein Tsr3 (predicted) [Schizosaccharomyces pombe 972h-].
156844447       Kpol_1037p24                    366     eukaryota>fungi>ascomycota                              Vanderwaltozyma polyspora DSM 70294                                             hypothetical protein Kpol_1037p24 [Vanderwaltozyma polyspora DSM 70294].
254580001       ZYRO0C07832g                    281     eukaryota>fungi>ascomycota                              Zygosaccharomyces rouxii CBS 732                                                ZYRO0C07832p [Zygosaccharomyces rouxii].
482812946       SETTUDRAFT_175653               395     eukaryota>fungi>ascomycota                              Setosphaeria turcica Et28A                                                      hypothetical protein SETTUDRAFT_175653 [Setosphaeria turcica Et28A].
448118269       GNLVRS01_PISO0D10167g           330     eukaryota>fungi>ascomycota                              Millerozyma farinosa CBS 7064                                                   Piso0_001064 [Millerozyma farinosa CBS 7064].
385302244       AWRI1499_3693                   279     eukaryota>fungi>ascomycota                              Dekkera bruxellensis AWRI1499                                                   yor006c-like protein [Dekkera bruxellensis AWRI1499].
475671285       FOC4_g10013057                  396     eukaryota>fungi>ascomycota                              Fusarium oxysporum f. sp. cubense race 4                                        Ribosome biogenesis protein TSR3 [Fusarium oxysporum f. sp. cubense race 4].
429860378       CGGC5_5131                      371     eukaryota>fungi>ascomycota                              Colletotrichum gloeosporioides Nara gc5                                         rli and duf367 domain protein [Colletotrichum gloeosporioides Nara gc5].
322708026       MAA_04532                       436     eukaryota>fungi>ascomycota                              Metarhizium anisopliae ARSEF 23                                                 RLI and DUF367 domain protein [Metarhizium anisopliae ARSEF 23].
315056111       MGYG_01506                      371     eukaryota>fungi>ascomycota                              Arthroderma gypseum CBS 118893                                                  RLI and DUF367 domain-containing protein [Arthroderma gypseum CBS 118893].
154278483       HCAG_05522                      368     eukaryota>fungi>ascomycota                              Ajellomyces capsulatus NAm1                                                     conserved hypothetical protein [Ajellomyces capsulatus NAm1].
119496443       NFIA_017990                     371     eukaryota>fungi>ascomycota                              Neosartorya fischeri NRRL 181                                                   RLI and DUF367 domain protein [Neosartorya fischeri NRRL 181].
225560602       HCBG_02420                      368     eukaryota>fungi>ascomycota                              Ajellomyces capsulatus G186AR                                                   RLI and DUF367 domain-containing protein [Ajellomyces capsulatus G186AR].
226288099       PADG_08232                      372     eukaryota>fungi>ascomycota                              Paracoccidioides brasiliensis Pb18                                              RLI and DUF367 domain-containing protein [Paracoccidioides brasiliensis Pb18].
347836151       BofuT4_P087860.1                398     eukaryota>fungi>ascomycota                              Botryotinia fuckeliana T4                                                       similar to RLI and DUF367 domain-containing protein [Botryotinia fuckeliana T4].
410084409       KAFR_0L00390                    329     eukaryota>fungi>ascomycota                              Kazachstania africana CBS 2517                                                  hypothetical protein KAFR_0L00390 [Kazachstania africana CBS 2517].
46123943        FG06349.1                       398     eukaryota>fungi>ascomycota                              Fusarium graminearum PH-1                                                       hypothetical protein FG06349.1 [Fusarium graminearum PH-1].
327294119       TERG_07374                      373     eukaryota>fungi>ascomycota                              Trichophyton rubrum CBS 118892                                                  RLI domain-containing protein [Trichophyton rubrum CBS 118892].
472241942       BcDW1_4654                      399     eukaryota>fungi>ascomycota                              Botryotinia fuckeliana BcDW1                                                    putative rli and duf367 domain protein [Botryotinia fuckeliana BcDW1].
408399557       FPSE_01144                      396     eukaryota>fungi>ascomycota                              Fusarium pseudograminearum CS3096                                               hypothetical protein FPSE_01144 [Fusarium pseudograminearum CS3096].
342883638       FOXB_05390                      399     eukaryota>fungi>ascomycota                              Fusarium oxysporum Fo5176                                                       hypothetical protein FOXB_05390 [Fusarium oxysporum Fo5176].
115389260       ATEG_02957                      373     eukaryota>fungi>ascomycota                              Aspergillus terreus NIH2624                                                     conserved hypothetical protein [Aspergillus terreus NIH2624].
154310001       BC1G_06921                      399     eukaryota>fungi>ascomycota                              Botryotinia fuckeliana B05.10                                                   hypothetical protein BC1G_06921 [Botryotinia fuckeliana B05.10].
367005114       TPHA_0J00320                    342     eukaryota>fungi>ascomycota                              Tetrapisispora phaffii CBS 4417                                                 hypothetical protein TPHA_0J00320 [Tetrapisispora phaffii CBS 4417].
146418571       PGUG_02980                      300     eukaryota>fungi>ascomycota                              Meyerozyma guilliermondii ATCC 6260                                             hypothetical protein PGUG_02980 [Meyerozyma guilliermondii ATCC 6260].
365985608       NDAI_0D00790                    401     eukaryota>fungi>ascomycota                              Naumovozyma dairenensis CBS 421                                                 hypothetical protein NDAI_0D00790 [Naumovozyma dairenensis CBS 421].
156039203       SS1G_11738                      401     eukaryota>fungi>ascomycota                              Sclerotinia sclerotiorum 1980 UF-70                                             hypothetical protein SS1G_11738 [Sclerotinia sclerotiorum 1980 UF-70].
213408234       SJAG_03755                      271     eukaryota>fungi>ascomycota                              Schizosaccharomyces japonicus yFS275                                            UPF0293 protein c [Schizosaccharomyces japonicus yFS275].
494830469       W97_06123                       397     eukaryota>fungi>ascomycota                              Coniosporium apollinis CBS 100218                                               hypothetical protein W97_06123 [Coniosporium apollinis CBS 100218].
320590998       CMQ_365                         403     eukaryota>fungi>ascomycota                              Grosmannia clavigera kw1407                                                     rli and duf367 domain containing protein [Grosmannia clavigera kw1407].
477533953       Cob_06228                       404     eukaryota>fungi>ascomycota                              Colletotrichum orbiculare MAFF 240422                                           rli and duf367 domain protein [Colletotrichum orbiculare MAFF 240422].
406605057       BN7_3079                        271     eukaryota>fungi>ascomycota                              Wickerhamomyces ciferrii                                                        hypothetical protein BN7_3079 [Wickerhamomyces ciferrii].
400601685       BBA_01275                       405     eukaryota>fungi>ascomycota                              Beauveria bassiana ARSEF 2860                                                   DUF367 domain-containing protein [Beauveria bassiana ARSEF 2860].
326480468       TEQG_03677                      374     eukaryota>fungi>ascomycota                              Trichophyton equinum CBS 127.97                                                 RLI and DUF367 domain-containing protein [Trichophyton equinum CBS 127.97].
323307122       FOSTERSO_4452                   190     eukaryota>fungi>ascomycota                              Saccharomyces cerevisiae FostersO                                               YOR006C-like protein [Saccharomyces cerevisiae FostersO].
346975695       VDAG_09481                      409     eukaryota>fungi>ascomycota                              Verticillium dahliae VdLs.17                                                    DUF367 domain-containing protein [Verticillium dahliae VdLs.17].
302404373       VDBG_09744                      412     eukaryota>fungi>ascomycota                              Verticillium alfalfae VaMs.102                                                  DUF367 domain-containing protein [Verticillium alfalfae VaMs.102].
367017059       TDEL_0G04500                    301     eukaryota>fungi>ascomycota                              Torulaspora delbrueckii                                                         hypothetical protein TDEL_0G04500 [Torulaspora delbrueckii].
295659211       PAAG_07853                      374     eukaryota>fungi>ascomycota                              Paracoccidioides sp. 'lutzii' Pb01                                              RLI and DUF367 domain-containing protein [Paracoccidioides sp. 'lutzii' Pb01].
477516032       FOC1_g10014096                  398     eukaryota>fungi>ascomycota                              Fusarium oxysporum f. sp. cubense race 1                                        Ribosome biogenesis protein TSR3 [Fusarium oxysporum f. sp. cubense race 1].
449303634       BAUCODRAFT_119218               414     eukaryota>fungi>ascomycota                              Baudoinia compniacensis UAMH 10762                                              hypothetical protein BAUCODRAFT_119218 [Baudoinia compniacensis UAMH 10762].
261206266       BDBG_02541                      374     eukaryota>fungi>ascomycota                              Ajellomyces dermatitidis SLH14081                                               RLI and DUF367 domain-containing protein [Ajellomyces dermatitidis SLH14081].
255724186       CTRG_01328                      344     eukaryota>fungi>ascomycota                              Candida tropicalis MYA-3404                                                     conserved hypothetical protein [Candida tropicalis MYA-3404].
255712845       KLTH0C11264g                    346     eukaryota>fungi>ascomycota                              Lachancea thermotolerans CBS 6340                                               KLTH0C11264p [Lachancea thermotolerans].
171679413       PODANSg1675                     416     eukaryota>fungi>ascomycota                              Podospora anserina S mat+                                                       hypothetical protein [Podospora anserina S mat+].
239610896       BDCG_03003                      374     eukaryota>fungi>ascomycota                              Ajellomyces dermatitidis ER-3                                                   RLI and DUF367 domain-containing protein [Ajellomyces dermatitidis ER-3].
238501456       AFLA_127690                     374     eukaryota>fungi>ascomycota                              Aspergillus flavus NRRL3357                                                     RLI and DUF367 domain protein [Aspergillus flavus NRRL3357].
340518847       TRIREDRAFT_77447                417     eukaryota>fungi>ascomycota                              Trichoderma reesei QM6a                                                         predicted protein [Trichoderma reesei QM6a].
169769024       AOR_1_814164                    374     eukaryota>fungi>ascomycota                              Aspergillus oryzae RIB40                                                        hypothetical protein AOR_1_814164 [Aspergillus oryzae RIB40].
452838392       DOTSEDRAFT_74960                420     eukaryota>fungi>ascomycota                              Dothistroma septosporum NZE10                                                   hypothetical protein DOTSEDRAFT_74960 [Dothistroma septosporum NZE10].
146322505       AFUA_1G06690                    374     eukaryota>fungi>ascomycota                              Aspergillus fumigatus Af293                                                     RLI and DUF367 domain protein [Aspergillus fumigatus Af293].
470317061       PNEG_02961                      191     eukaryota>fungi>ascomycota                              Pneumocystis murina B123                                                        hypothetical protein PNEG_02961 [Pneumocystis murina B123].
358394332       TRIATDRAFT_244323               420     eukaryota>fungi>ascomycota                              Trichoderma atroviride IMI 206040                                               Conserved hypothetical protein [Trichoderma atroviride IMI 206040].
240280173       HCDG_01707                      368     eukaryota>fungi>ascomycota                              Ajellomyces capsulatus H143                                                     RLI and DUF367 domain-containing protein [Ajellomyces capsulatus H143].
303316696       CPC735_003740                   375     eukaryota>fungi>ascomycota                              Coccidioides posadasii C735 delta SOWgp                                         hypothetical protein CPC735_003740 [Coccidioides posadasii C735 delta SOWgp].
444314751       TBLA_0A07250                    304     eukaryota>fungi>ascomycota                              Tetrapisispora blattae CBS 6284                                                 hypothetical protein TBLA_0A07250 [Tetrapisispora blattae CBS 6284].
302915407       NECHADRAFT_16258                375     eukaryota>fungi>ascomycota                              Nectria haematococca mpVI 77-13-4                                               hypothetical protein NECHADRAFT_16258, partial [Nectria haematococca mpVI 77-13-4].
121702447       ACLA_027870                     375     eukaryota>fungi>ascomycota                              Aspergillus clavatus NRRL 1                                                     RLI and DUF367 domain protein [Aspergillus clavatus NRRL 1].
241951064       CD36_17770                      346     eukaryota>fungi>ascomycota                              Candida dubliniensis CD36                                                       uncharacterized metal-binding protein, putative [Candida dubliniensis CD36].
358385694       TRIVIDRAFT_56125                421     eukaryota>fungi>ascomycota                              Trichoderma virens Gv29-8                                                       hypothetical protein TRIVIDRAFT_56125 [Trichoderma virens Gv29-8].
336467234       NEUTE1DRAFT_85671               421     eukaryota>fungi>ascomycota                              Neurospora tetrasperma FGSC 2508                                                hypothetical protein NEUTE1DRAFT_85671 [Neurospora tetrasperma FGSC 2508].
501756311       TAPDE_000234                    265     eukaryota>fungi>ascomycota                              Taphrina deformans PYCC 5710                                                    UPF0293 protein C1F3.04c [Taphrina deformans PYCC 5710].
85094874        NCU06104                        421     eukaryota>fungi>ascomycota                              Neurospora crassa OR74A                                                         hypothetical protein NCU06104 [Neurospora crassa OR74A].
403218478       KNAG_0M01150                    307     eukaryota>fungi>ascomycota                              Kazachstania naganishii CBS 8797                                                hypothetical protein KNAG_0M01150 [Kazachstania naganishii CBS 8797].
119187919       CIMG_04007                      375     eukaryota>fungi>ascomycota                              Coccidioides immitis RS                                                         hypothetical protein CIMG_04007 [Coccidioides immitis RS].
50286755        CAGL0E02717g                    325     eukaryota>fungi>ascomycota                              Candida glabrata CBS 138                                                        hypothetical protein [Candida glabrata CBS 138].
238882095       CAWG_04067                      346     eukaryota>fungi>ascomycota                              Candida albicans WO-1                                                           conserved hypothetical protein [Candida albicans WO-1].
350630798       ASPNIDRAFT_202631               377     eukaryota>fungi>ascomycota                              Aspergillus niger ATCC 1015                                                     hypothetical protein ASPNIDRAFT_202631, partial [Aspergillus niger ATCC 1015].
452978378       MYCFIDRAFT_205413               422     eukaryota>fungi>ascomycota                              Pseudocercospora fijiensis CIRAD86                                              hypothetical protein MYCFIDRAFT_205413 [Pseudocercospora fijiensis CIRAD86].
145255636       ANI_1_760164                    378     eukaryota>fungi>ascomycota                              Aspergillus niger CBS 513.88                                                    hypothetical protein ANI_1_760164 [Aspergillus niger CBS 513.88].
258576519       UREG_01957                      379     eukaryota>fungi>ascomycota                              Uncinocarpus reesii 1704                                                        hypothetical protein UREG_01957 [Uncinocarpus reesii 1704].
485922855       UCRNP2_5087                     383     eukaryota>fungi>ascomycota                              Neofusicoccum parvum UCRNP2                                                     putative rli and duf367 domain protein [Neofusicoccum parvum UCRNP2].
452004414       COCHEDRAFT_1018606              397     eukaryota>fungi>ascomycota                              Bipolaris maydis C5                                                             hypothetical protein COCHEDRAFT_1018606 [Bipolaris maydis C5].
126131620       PICST_81457                     309     eukaryota>fungi>ascomycota                              Scheffersomyces stipitis CBS 6054                                               hypothetical protein PICST_81457 [Scheffersomyces stipitis CBS 6054].
358373436       AKAW_08148                      383     eukaryota>fungi>ascomycota                              Aspergillus kawachii IFO 4308                                                   RLI and DUF367 domain protein [Aspergillus kawachii IFO 4308].
440636275       GMDG_07849                      387     eukaryota>fungi>ascomycota                              Pseudogymnoascus destructans 20631-21                                           hypothetical protein GMDG_07849 [Pseudogymnoascus destructans 20631-21].
68481408        CaO19.5802                      346     eukaryota>fungi>ascomycota                              Candida albicans SC5314                                                         hypothetical protein CaO19.5802 [Candida albicans SC5314].
255942811       Pc18g03350                      367     eukaryota>fungi>ascomycota                              Penicillium chrysogenum Wisconsin 54-1255                                       Pc18g03350 [Penicillium chrysogenum Wisconsin 54-1255].
302652345       TRV_07966                       258     eukaryota>fungi>ascomycota                              Trichophyton verrucosum HKI 0517                                                hypothetical protein TRV_07966 [Trichophyton verrucosum HKI 0517].
344303169       SPAPADRAFT_60797                324     eukaryota>fungi>ascomycota                              Spathaspora passalidarum NRRL Y-27907                                           hypothetical protein SPAPADRAFT_60797, partial [Spathaspora passalidarum NRRL Y-27907].
302503194       ARB_00004                       256     eukaryota>fungi>ascomycota                              Arthroderma benhamiae CBS 112371                                                hypothetical protein ARB_00004 [Arthroderma benhamiae CBS 112371].
296419901       GSTUM_00007521001               347     eukaryota>fungi>ascomycota                              Tuber melanosporum Mel28                                                        hypothetical protein [Tuber melanosporum Mel28].
50551087        YALI0D19118g                    348     eukaryota>fungi>ascomycota                              Yarrowia lipolytica CLIB122                                                     YALI0D19118p [Yarrowia lipolytica].
225683062       PABG_07865                      323     eukaryota>fungi>ascomycota                              Paracoccidioides brasiliensis Pb03                                              DUF367 family protein [Paracoccidioides brasiliensis Pb03].
340966722       CTHT_0017460                    436     eukaryota>fungi>ascomycota                              Chaetomium thermophilum var. thermophilum DSM 1495                              hypothetical protein CTHT_0017460 [Chaetomium thermophilum var. thermophilum DSM 1495].
149248380       LELG_01097                      390     eukaryota>fungi>ascomycota                              Lodderomyces elongisporus NRRL YB-4239                                          conserved hypothetical protein [Lodderomyces elongisporus NRRL YB-4239].
366995956       NCAS_0H00810                    323     eukaryota>fungi>ascomycota                              Naumovozyma castellii CBS 4309                                                  hypothetical protein NCAS_0H00810 [Naumovozyma castellii CBS 4309].
406864608       MBM_04021                       391     eukaryota>fungi>ascomycota                              Marssonina brunnea f. sp. 'multigermtubi' MB_m1                                 RLI and DUF367 domain protein [Marssonina brunnea f. sp. 'multigermtubi' MB_m1].
323303064       FOSTERSB_4333                   205     eukaryota>fungi>ascomycota                              Saccharomyces cerevisiae FostersB                                               YOR006C-like protein [Saccharomyces cerevisiae FostersB].
399169358       CPUR_03724                      435     eukaryota>fungi>ascomycota                              Claviceps purpurea 20.1                                                         uncharacterized protein CPUR_03724 [Claviceps purpurea 20.1].
363750278       Ecym_3023                       349     eukaryota>fungi>ascomycota                              Eremothecium cymbalariae DBVPG#7215                                             hypothetical protein Ecym_3023 [Eremothecium cymbalariae DBVPG#7215].
169617546       SNOG_11955                      394     eukaryota>fungi>ascomycota                              Phaeosphaeria nodorum SN15                                                      hypothetical protein SNOG_11955 [Phaeosphaeria nodorum SN15].
398390005       MYCGRDRAFT_77085                391     eukaryota>fungi>ascomycota                              Zymoseptoria tritici IPO323                                                     hypothetical protein MYCGRDRAFT_77085 [Zymoseptoria tritici IPO323].
365758437       VIN7_9867                       312     eukaryota>fungi>ascomycota                              Saccharomyces cerevisiae x Saccharomyces kudriavzevii VIN7                      YOR006C-like protein [Saccharomyces cerevisiae x Saccharomyces kudriavzevii VIN7].
326472825       TESG_04262                      351     eukaryota>fungi>ascomycota                              Trichophyton tonsurans CBS 112818                                               RLI domain-containing protein [Trichophyton tonsurans CBS 112818].
116203705       CHGG_09736                      423     eukaryota>fungi>ascomycota                              Chaetomium globosum CBS 148.51                                                  hypothetical protein CHGG_09736 [Chaetomium globosum CBS 148.51].
242766849       TSTA_072560                     391     eukaryota>fungi>ascomycota                              Talaromyces stipitatus ATCC 10500                                               RLI and DUF367 domain protein [Talaromyces stipitatus ATCC 10500].
367048081       THITE_2117442                   432     eukaryota>fungi>ascomycota                              Thielavia terrestris NRRL 8126                                                  hypothetical protein THITE_2117442 [Thielavia terrestris NRRL 8126].
448513508       CORT_0A11510                    355     eukaryota>fungi>ascomycota                              Candida orthopsilosis Co 90-125                                                 Tsr3 protein [Candida orthopsilosis Co 90-125].
212528282       PMAA_026280                     391     eukaryota>fungi>ascomycota                              Talaromyces marneffei ATCC 18224                                                RLI and DUF367 domain protein [Talaromyces marneffei ATCC 18224].
471573094       UCREL1_1630                     425     eukaryota>fungi>ascomycota                              Eutypa lata UCREL1                                                              putative duf367 domain-containing protein [Eutypa lata UCREL1].
344234524       CANTEDRAFT_112122               321     eukaryota>fungi>ascomycota                              Candida tenuis ATCC 10573                                                       DUF367-domain-containing protein [Candida tenuis ATCC 10573].
50306295        KLLA0D01111g                    314     eukaryota>fungi>ascomycota                              Kluyveromyces lactis NRRL Y-1140                                                hypothetical protein [Kluyveromyces lactis NRRL Y-1140].
380494387       CH063_00927                     392     eukaryota>fungi>ascomycota                              Colletotrichum higginsianum                                                     hypothetical protein CH063_00927 [Colletotrichum higginsianum].
361127766       M7I_4404                        393     eukaryota>fungi>ascomycota                              Glarea lozoyensis 74030                                                         putative Ribosome biogenesis protein TSR3 [Glarea lozoyensis 74030].
354546841       CPAR2_212170                    355     eukaryota>fungi>ascomycota                              Candida parapsilosis                                                            hypothetical protein CPAR2_212170 [Candida parapsilosis].
389640989       MGG_00851                       425     eukaryota>fungi>ascomycota                              Magnaporthe oryzae 70-15                                                        hypothetical protein MGG_00851 [Magnaporthe oryzae 70-15].
396483276       LEMA_P095980.1                  397     eukaryota>fungi>ascomycota                              Leptosphaeria maculans JN3                                                      similar to RLI and DUF367 domain-containing protein [Leptosphaeria maculans JN3].
330926298       PTT_12896                       393     eukaryota>fungi>ascomycota                              Pyrenophora teres f. teres 0-1                                                  hypothetical protein PTT_12896 [Pyrenophora teres f. teres 0-1].
374106616       FAGOS_FACR007W                  313     eukaryota>fungi>ascomycota                              Ashbya gossypii FDAG1                                                           FACR007Wp [Ashbya gossypii FDAG1].
349581171       SYK7_063801                     313     eukaryota>fungi>ascomycota                              Saccharomyces cerevisiae Kyokai no. 7                                           K7_Yor006cp [Saccharomyces cerevisiae Kyokai no. 7].
310796322       GLRG_06758                      393     eukaryota>fungi>ascomycota                              Colletotrichum graminicola M1.001                                               hypothetical protein GLRG_06758 [Colletotrichum graminicola M1.001].
322697535       MAC_04695                       425     eukaryota>fungi>ascomycota                              Metarhizium acridum CQMa 102                                                    RLI and DUF367 domain protein [Metarhizium acridum CQMa 102].
453080662       SEPMUDRAFT_151678               431     eukaryota>fungi>ascomycota                              Sphaerulina musiva SO2202                                                       DUF367-domain-containing protein [Sphaerulina musiva SO2202].
190407347       SCRG_01408                      313     eukaryota>fungi>ascomycota                              Saccharomyces cerevisiae RM11-1a                                                conserved hypothetical protein [Saccharomyces cerevisiae RM11-1a].
6324579         YOR006C                         313     eukaryota>fungi>ascomycota                              Saccharomyces cerevisiae S288c                                                  Tsr3p [Saccharomyces cerevisiae S288c].
367031724       MYCTH_2308548                   427     eukaryota>fungi>ascomycota                              Myceliophthora thermophila ATCC 42464                                           hypothetical protein MYCTH_2308548 [Myceliophthora thermophila ATCC 42464].
346327014       CCM_01268                       394     eukaryota>fungi>ascomycota                              Cordyceps militaris CM01                                                        RLI and DUF367 domain protein [Cordyceps militaris CM01].
296822836       MCYG_00453                      357     eukaryota>fungi>ascomycota                              Arthroderma otae CBS 113480                                                     RLI and DUF367 domain-containing protein [Arthroderma otae CBS 113480].
345568311       AOL_s00054g584                  394     eukaryota>fungi>ascomycota                              Arthrobotrys oligospora ATCC 24927                                              hypothetical protein AOL_s00054g584 [Arthrobotrys oligospora ATCC 24927].
45185694        AGOS_ACR007W                    313     eukaryota>fungi>ascomycota                              Ashbya gossypii ATCC 10895                                                      ACR007Wp [Ashbya gossypii ATCC 10895].
323352380       VL3_4393                        217     eukaryota>fungi>ascomycota                              Saccharomyces cerevisiae VL3                                                    YOR006C-like protein [Saccharomyces cerevisiae VL3].
500259147       UCRPA7_2514                     402     eukaryota>fungi>ascomycota                              Togninia minima UCRPA7                                                          putative duf367 domain-containing protein [Togninia minima UCRPA7].
189194643       PTRG_03327                      394     eukaryota>fungi>ascomycota                              Pyrenophora tritici-repentis Pt-1C-BFP                                          hypothetical protein PTRG_03327 [Pyrenophora tritici-repentis Pt-1C-BFP].
151945635       SCY_5079                        313     eukaryota>fungi>ascomycota                              Saccharomyces cerevisiae YJM789                                                 conserved protein [Saccharomyces cerevisiae YJM789].
336259923       SMAC_06415                      426     eukaryota>fungi>ascomycota                              Sordaria macrospora k-hell                                                      hypothetical protein SMAC_06415 [Sordaria macrospora k-hell].
407917357       MPH_12250                       468     eukaryota>fungi>ascomycota                              Macrophomina phaseolina MS6                                                     protein of unknown function DUF367 [Macrophomina phaseolina MS6].
384500854       RO3G_16056                      249     eukaryota>fungi                                         Rhizopus delemar RA 99-880                                                      hypothetical protein RO3G_16056 [Rhizopus delemar RA 99-880].
389602659       LBRM_32_2170                    293     eukaryota>euglenozoa>kinetoplastida                     Leishmania braziliensis MHOM/BR/75/M2904                                        conserved hypothetical protein [Leishmania braziliensis MHOM/BR/75/M2904].
74026018        Tb11.01.6830                    264     eukaryota>euglenozoa>kinetoplastida                     Trypanosoma brucei brucei strain 927/4 GUTat10.1                                hypothetical protein [Trypanosoma brucei brucei strain 927/4 GUTat10.1].
261335591       TbgDal_XI17050                  264     eukaryota>euglenozoa>kinetoplastida                     Trypanosoma brucei gambiense DAL972                                             hypothetical protein, conserved [Trypanosoma brucei gambiense DAL972].
401427121       LMXM_31_1970                    297     eukaryota>euglenozoa>kinetoplastida                     Leishmania mexicana MHOM/GT/2001/U1103                                          conserved hypothetical protein [Leishmania mexicana MHOM/GT/2001/U1103].
398020920       LDBPK_322090                    297     eukaryota>euglenozoa>kinetoplastida                     Leishmania donovani                                                             hypothetical protein, conserved [Leishmania donovani].
407411057       MOQ_002857                      278     eukaryota>euglenozoa>kinetoplastida                     Trypanosoma cruzi marinkellei                                                   hypothetical protein MOQ_002857 [Trypanosoma cruzi marinkellei].
157874046       LMJF_32_1970                    297     eukaryota>euglenozoa>kinetoplastida                     Leishmania major strain Friedlin                                                conserved hypothetical protein [Leishmania major strain Friedlin].
340059722       TVY486_1116000                  259     eukaryota>euglenozoa>kinetoplastida                     Trypanosoma vivax Y486                                                          conserved hypothetical protein [Trypanosoma vivax Y486].
71410179        Tc00.1047053503987.70           239     eukaryota>euglenozoa>kinetoplastida                     Trypanosoma cruzi strain CL Brener                                              hypothetical protein [Trypanosoma cruzi strain CL Brener].
146096672       LINJ_32_2090                    297     eukaryota>euglenozoa>kinetoplastida                     Leishmania infantum JPCM5                                                       conserved hypothetical protein [Leishmania infantum JPCM5].
71405309        Tc00.1047053511713.40           322     eukaryota>euglenozoa>kinetoplastida                     Trypanosoma cruzi strain CL Brener                                              hypothetical protein [Trypanosoma cruzi strain CL Brener].
342186530       TCIL3000_11_15340               264     eukaryota>euglenozoa>kinetoplastida                     Trypanosoma congolense IL3000                                                   unnamed protein product [Trypanosoma congolense IL3000].
407850436       TCSYLVIO_004103                 269     eukaryota>euglenozoa>kinetoplastida                     Trypanosoma cruzi                                                               hypothetical protein TCSYLVIO_004103 [Trypanosoma cruzi].
428178277       GUITHDRAFT_69967                209     eukaryota>cryptophyta                                   Guillardia theta CCMP2712                                                       hypothetical protein GUITHDRAFT_69967 [Guillardia theta CCMP2712].
326436441       PTSG_02697                      333     eukaryota>choanoflagellida                              Salpingoeca rosetta                                                             hypothetical protein PTSG_02697 [Salpingoeca rosetta].
167516942       MONBRDRAFT_13793                303     eukaryota>choanoflagellida                              Monosiga brevicollis MX1                                                        hypothetical protein [Monosiga brevicollis MX1].
470265735       DFA_05876                       338     eukaryota>amoebozoa>mycetozoa>dictyosteliida            Dictyostelium fasciculatum                                                      Hypothetical UPF0293 protein [Dictyostelium fasciculatum].
330845784       DICPUDRAFT_20500                170     eukaryota>amoebozoa>mycetozoa>dictyosteliida            Dictyostelium purpureum                                                         hypothetical protein DICPUDRAFT_20500, partial [Dictyostelium purpureum].
66827959        DDB_G0267822                    365     eukaryota>amoebozoa>mycetozoa>dictyosteliida            Dictyostelium discoideum AX4                                                    hypothetical protein DDB_G0267822 [Dictyostelium discoideum AX4].
281212362       PPL_00317                       362     eukaryota>amoebozoa>mycetozoa>dictyosteliida            Polysphondylium pallidum PN500                                                  Hypothetical UPF0293 protein [Polysphondylium pallidum PN500].
449702338       EHI5A_035350                    231     eukaryota>amoebozoa>entamoebidae                        Entamoeba histolytica KU27                                                      metalbinding family, in rnase L inhibitor RLI family protein, putative [Entamoeba histolytica KU27].
67479453        EHI_100490                      234     eukaryota>amoebozoa>entamoebidae                        Entamoeba histolytica HM-1:IMSS                                                 hypothetical protein [Entamoeba histolytica HM-1:IMSS].
167383085       EDI_129860                      231     eukaryota>amoebozoa>entamoebidae                        Entamoeba dispar SAW760                                                         hypothetical protein [Entamoeba dispar SAW760].
407034727       ENU1_200140                     227     eukaryota>amoebozoa>entamoebidae                        Entamoeba nuttalli P19                                                          RNase L inhibitor (RLI) domain-containing protein [Entamoeba nuttalli P19].
471197998       EIN_484800                      227     eukaryota>amoebozoa>entamoebidae                        Entamoeba invadens IP1                                                          hypothetical protein EIN_484800 [Entamoeba invadens IP1].
472464689       KM1_043280                      227     eukaryota>amoebozoa>entamoebidae                        Entamoeba histolytica HM-3:IMSS                                                 UPF0293 family protein [Entamoeba histolytica HM-3:IMSS].
470383761       ACA1_400580                     293     eukaryota>amoebozoa>acanthamoebidae                     Acanthamoeba castellanii str. Neff                                              hypothetical protein ACA1_400580 [Acanthamoeba castellanii str. Neff].
145505483       GSPATT00001025001               256     eukaryota>alveolata>ciliophora                          Paramecium tetraurelia strain d4-2                                              hypothetical protein [Paramecium tetraurelia strain d4-2].
145507686       GSPATT00008843001               255     eukaryota>alveolata>ciliophora                          Paramecium tetraurelia strain d4-2                                              hypothetical protein [Paramecium tetraurelia strain d4-2].
403355917       OXYTRI_00818                    261     eukaryota>alveolata>ciliophora                          Oxytricha trifallax                                                             DUF367 family protein [Oxytricha trifallax].
403369769       OXYTRI_17418                    332     eukaryota>alveolata>ciliophora                          Oxytricha trifallax                                                             DUF367 family protein [Oxytricha trifallax].
457877450       PCYB_144630                     243     eukaryota>alveolata>apicomplexa                         Plasmodium cynomolgi strain B                                                   hypothetical protein PCYB_144630 [Plasmodium cynomolgi strain B].
403222007       TOT_020000402                   248     eukaryota>alveolata>apicomplexa                         Theileria orientalis strain Shintoku                                            uncharacterized protein TOT_020000402 [Theileria orientalis strain Shintoku].
156088527       BBOV_III005390                  270     eukaryota>alveolata>apicomplexa                         Babesia bovis T2Bo                                                              metal-binding domain in RNase L inhibitor, RLI family protein [Babesia bovis].
429327544       BEWA_021520                     250     eukaryota>alveolata>apicomplexa                         Babesia equi                                                                    hypothetical protein BEWA_021520 [Babesia equi].
209876904       CMU_025520                      290     eukaryota>alveolata>apicomplexa                         Cryptosporidium muris RN66                                                      hypothetical protein [Cryptosporidium muris RN66].
66359774        cgd8_1580                       330     eukaryota>alveolata>apicomplexa                         Cryptosporidium parvum Iowa II                                                  saccharomyces Yor006cp like protein conserved across euks and archaea [Cryptosporidium parvum Iowa II].
82752449        PY06626                         339     eukaryota>alveolata>apicomplexa                         Plasmodium yoelii yoelii 17XNL                                                  hypothetical protein [Plasmodium yoelii yoelii 17XNL].
67587365        Chro.80186                      324     eukaryota>alveolata>apicomplexa                         Cryptosporidium hominis TU502                                                   hypothetical protein [Cryptosporidium hominis TU502].
399218872       BBM_III07860                    267     eukaryota>alveolata>apicomplexa                         Babesia microti strain RI                                                       unnamed protein product [Babesia microti strain RI].
156103205       PVX_123665                      321     eukaryota>alveolata>apicomplexa                         Plasmodium vivax Sal-1                                                          hypothetical protein [Plasmodium vivax Sal-1].
71030664        TP02_0408                       284     eukaryota>alveolata>apicomplexa                         Theileria parva strain Muguga                                                   hypothetical protein [Theileria parva strain Muguga].
124806015       PFL0985c                        318     eukaryota>alveolata>apicomplexa                         Plasmodium falciparum 3D7                                                       conserved protein [Plasmodium falciparum 3D7].
84995170        TA13030                         271     eukaryota>alveolata>apicomplexa                         Theileria annulata strain Ankara                                                hypothetical protein [Theileria annulata strain Ankara].
221061411       PKH_143710                      309     eukaryota>alveolata>apicomplexa                         Plasmodium knowlesi strain H                                                    hypothetical protein [Plasmodium knowlesi strain H].
68069837        PB000582.02.0                   307     eukaryota>alveolata>apicomplexa                         Plasmodium berghei ANKA                                                         hypothetical protein, partial [Plasmodium berghei ANKA].
70929412        PC301627.00.0                   157     eukaryota>alveolata>apicomplexa                         Plasmodium chabaudi chabaudi                                                    hypothetical protein, partial [Plasmodium chabaudi chabaudi].
294941345       Pmar_PMAR000998                 168     eukaryota>alveolata                                     Perkinsus marinus ATCC 50983                                                    conserved hypothetical protein, partial [Perkinsus marinus ATCC 50983].
294933932       Pmar_PMAR025335                 231     eukaryota>alveolata                                     Perkinsus marinus ATCC 50983                                                    conserved hypothetical protein [Perkinsus marinus ATCC 50983].
308159944       GLP15_2356                      227     eukaryota                                               Giardia lamblia P15                                                             RNase P RNA component [Giardia lamblia P15].
470288840       CAOG_08090                      358     eukaryota                                               Capsaspora owczarzaki ATCC 30864                                                metal-binding domain in RNase L inhibitor [Capsaspora owczarzaki ATCC 30864].
253744184       GL50581_2321                    250     eukaryota                                               Giardia intestinalis ATCC 50581                                                 RNase P RNA component [Giardia intestinalis ATCC 50581].
159110411       GL50803_16463                   320     eukaryota                                               Giardia lamblia ATCC 50803                                                      RNase P RNA component [Giardia lamblia ATCC 50803].
395329661       DICSQDRAFT_126764               282     eukaryota>fungi>basidiomycota                           Dichomitus squalens LYAD-421 SS1                                                hypothetical protein DICSQDRAFT_126764 [Dichomitus squalens LYAD-421 SS1].
#;TDD solo domain
170290747       Kcr_1134                        170     archaea>korarchaeota                                    Candidatus Korarchaeum cryptofilum OPF8                                         hypothetical protein Kcr_1134 [Candidatus Korarchaeum cryptofilum OPF8].
492956871       -                               179     archaea>euryarchaeota                                   Natronorubrum bangense                                                          hypothetical protein [Natronorubrum bangense].
257386814       -                               178     archaea>euryarchaeota                                   Halomicrobium mukohataei DSM 12286                                              hypothetical protein Hmuk_0749 [Halomicrobium mukohataei DSM 12286].
494233474       -                               181     archaea>euryarchaeota                                   Halobiforma lacisalsi                                                           hypothetical protein [Halobiforma lacisalsi].
150401096       Maeo_0666                       177     archaea>euryarchaeota                                   Methanococcus aeolicus Nankai-3                                                 hypothetical protein Maeo_0666 [Methanococcus aeolicus Nankai-3].
154151992       -                               163     archaea>euryarchaeota                                   Methanoregula boonei 6A8                                                        hypothetical protein Mboo_2453 [Methanoregula boonei 6A8].
497148103       -                               177     archaea>euryarchaeota                                   Halobacterium sp. DL1                                                           hypothetical protein [Halobacterium sp. DL1].
492985776       -                               198     archaea>euryarchaeota                                   Halococcus saccharolyticus                                                      hypothetical protein [Halococcus saccharolyticus].
432329633       -                               163     archaea>euryarchaeota                                   Methanoregula formicica SMSP                                                    hypothetical protein Metfor_0187 [Methanoregula formicica SMSP].
493010493       -                               176     archaea>euryarchaeota                                   Natronorubrum tibetense                                                         hypothetical protein [Natronorubrum tibetense].
493044943       -                               176     archaea>euryarchaeota                                   Natrialba asiatica                                                              hypothetical protein [Natrialba asiatica].
494470589       -                               176     archaea>euryarchaeota                                   Natronolimnobius innermongolicus                                                hypothetical protein [Natronolimnobius innermongolicus].
495659530       -                               176     archaea>euryarchaeota                                   Halosarcina pallida                                                             hypothetical protein [Halosarcina pallida].
147920882       -                               175     archaea>euryarchaeota                                   Methanocella arvoryzae MRE50                                                    hypothetical protein RCIX578 [Methanocella arvoryzae MRE50].
289581114       -                               175     archaea>euryarchaeota                                   Natrialba magadii ATCC 43099                                                    hypothetical protein Nmag_1439 [Natrialba magadii ATCC 43099].
491181685       -                               163     archaea>euryarchaeota                                   Halococcus salifodinae                                                          hypothetical protein [Halococcus salifodinae].
336253413       -                               175     archaea>euryarchaeota                                   Halopiger xanaduensis SH-6                                                      hypothetical protein Halxa_2016 [Halopiger xanaduensis SH-6].
495801004       -                               183     archaea>euryarchaeota                                   Halorhabdus tiamatea                                                            hypothetical protein [Halorhabdus tiamatea].
493878771       -                               175     archaea>euryarchaeota                                   Natrialba taiwanensis                                                           hypothetical protein [Natrialba taiwanensis].
20091339        -                               174     archaea>euryarchaeota                                   Methanosarcina acetivorans C2A                                                  hypothetical protein MA2508 [Methanosarcina acetivorans C2A].
126180409       -                               163     archaea>euryarchaeota                                   Methanoculleus marisnigri JR1                                                   hypothetical protein Memar_2471 [Methanoculleus marisnigri JR1].
494525312       -                               163     archaea>euryarchaeota                                   Methanolinea tarda                                                              hypothetical protein [Methanolinea tarda].
296110056       Metin_1391                      174     archaea>euryarchaeota                                   Methanocaldococcus infernus ME                                                  Protein of unknown function DUF367 [methanocaldococcus infernus ME].
312136516       Mfer_0288                       174     archaea>euryarchaeota                                   Methanothermus fervidus DSM 2088                                                hypothetical protein Mfer_0288 [Methanothermus fervidus DSM 2088].
116754117       Mthe_0806                       162     archaea>euryarchaeota                                   Methanosaeta thermophila PT                                                     hypothetical protein Mthe_0806 [Methanosaeta thermophila PT].
385804612       -                               184     archaea>euryarchaeota                                   Haloquadratum walsbyi C23                                                       UPF0293 family protein [Haloquadratum walsbyi C23].
478483668       MMALV_16820                     174     archaea>euryarchaeota                                   Candidatus Methanomethylophilus alvus Mx1201                                    Ribosome biogenesis protein TSR3 [Candidatus Methanomethylophilus alvus Mx1201].
493702897       -                               174     archaea>euryarchaeota                                   Natrialba hulunbeirensis                                                        hypothetical protein [Natrialba hulunbeirensis].
21229176        -                               173     archaea>euryarchaeota                                   Methanosarcina mazei Go1                                                        hypothetical protein MM_3074 [Methanosarcina mazei Go1].
73669897        -                               173     archaea>euryarchaeota                                   Methanosarcina barkeri str. Fusaro                                              conserved hypothetical protein [Methanosarcina barkeri str. Fusaro].
91772165        Mbur_0088                       173     archaea>euryarchaeota                                   Methanococcoides burtonii DSM 6242                                              hypothetical protein Mbur_0088 [Methanococcoides burtonii DSM 6242].
16082472        Ta0023m                         163     archaea>euryarchaeota                                   Thermoplasma acidophilum DSM 1728                                               hypothetical protein Ta0023m [Thermoplasma acidophilum DSM 1728].
298675558       Metev_1672                      173     archaea>euryarchaeota                                   Methanohalobium evestigatum Z-7303                                              hypothetical protein Metev_1672 [Methanohalobium evestigatum Z-7303].
497572677       -                               164     archaea>euryarchaeota                                   Ferroplasma acidarmanus                                                         ribosome biogenesis protein [Ferroplasma acidarmanus].
336476648       -                               173     archaea>euryarchaeota                                   Methanosalsum zhilinae DSM 4017                                                 hypothetical protein [Methanosalsum zhilinae DSM 4017].
261403576       Metvu_1465                      184     archaea>euryarchaeota                                   Methanocaldococcus vulcanius M7                                                 hypothetical protein Metvu_1465 [Methanocaldococcus vulcanius M7].
110669024       HQ3139A                         184     archaea>euryarchaeota                                   Haloquadratum walsbyi DSM 16790                                                 hypothetical protein HQ3139A [Haloquadratum walsbyi DSM 16790].
435851877       -                               173     archaea>euryarchaeota                                   Methanomethylovorans hollandica DSM 15978                                       hypothetical protein Metho_1744 [Methanomethylovorans hollandica DSM 15978].
452211569       -                               173     archaea>euryarchaeota                                   Methanosarcina mazei Tuc01                                                      Ribosome biogenesis protein TSR3 [Methanosarcina mazei Tuc01].
15668890        MJ_0708                         172     archaea>euryarchaeota                                   Methanocaldococcus jannaschii DSM 2661                                          hypothetical protein MJ_0708 [Methanocaldococcus jannaschii DSM 2661].
490178381       -                               164     archaea>euryarchaeota                                   Methanoplanus limicola                                                          hypothetical protein [Methanoplanus limicola].
288560356       mru_1099                        172     archaea>euryarchaeota                                   Methanobrevibacter ruminantium M1                                               hypothetical protein mru_1099 [Methanobrevibacter ruminantium M1].
330507271       MCON_1157                       160     archaea>euryarchaeota                                   Methanosaeta concilii GP6                                                       hypothetical protein MCON_1157 [Methanosaeta concilii GP6].
333986611       -                               172     archaea>euryarchaeota                                   Methanobacterium sp. SWAN-1                                                     hypothetical protein MSWAN_0374 [Methanobacterium sp. SWAN-1].
491703955       -                               186     archaea>euryarchaeota                                   Natronococcus amylolyticus                                                      hypothetical protein [Natronococcus amylolyticus].
494103868       -                               149     archaea>euryarchaeota                                   Methanotorris formicicus                                                        ribosome biogenesis protein MJ0708 [Methanotorris formicicus].
493938660       -                               172     archaea>euryarchaeota                                   Halosimplex carlsbadense                                                        hypothetical protein [Halosimplex carlsbadense].
494169254       -                               172     archaea>euryarchaeota                                   Natrinema altunense                                                             hypothetical protein [Natrinema altunense].
45357713        MMP0150                         171     archaea>euryarchaeota                                   Methanococcus maripaludis S2                                                    hypothetical protein MMP0150 [Methanococcus maripaludis S2].
84490081        Msp_1293                        171     archaea>euryarchaeota                                   Methanosphaera stadtmanae DSM 3091                                              hypothetical protein Msp_1293 [Methanosphaera stadtmanae DSM 3091].
134046551       MmarC5_1525                     171     archaea>euryarchaeota                                   Methanococcus maripaludis C5                                                    hypothetical protein MmarC5_1525 [Methanococcus maripaludis C5].
148642186       Msm_0126                        171     archaea>euryarchaeota                                   Methanobrevibacter smithii ATCC 35061                                           hypothetical protein Msm_0126 [Methanobrevibacter smithii ATCC 35061].
150399898       Mevan_1155                      171     archaea>euryarchaeota                                   Methanococcus vannielii SB                                                      hypothetical protein Mevan_1155 [Methanococcus vannielii SB].
150403073       MmarC7_1150                     171     archaea>euryarchaeota                                   Methanococcus maripaludis C7                                                    hypothetical protein MmarC7_1150 [Methanococcus maripaludis C7].
289192936       MFS40622_1467                   171     archaea>euryarchaeota                                   Methanocaldococcus sp. FS406-22                                                 Protein of unknown function DUF367 [Methanocaldococcus sp. FS406-22].
313125299       -                               171     archaea>euryarchaeota                                   Halogeometricum borinquense DSM 11551                                           hypothetical protein Hbor_05230 [Halogeometricum borinquense DSM 11551].
493724368       -                               187     archaea>euryarchaeota                                   Halobiforma nitratireducens                                                     hypothetical protein [Halobiforma nitratireducens].
336122367       Metok_1399                      171     archaea>euryarchaeota                                   Methanothermococcus okinawensis IH1                                             hypothetical protein Metok_1399 [Methanothermococcus okinawensis IH1].
340623331       GYY_00765                       171     archaea>euryarchaeota                                   Methanococcus maripaludis X1                                                    hypothetical protein GYY_00765 [Methanococcus maripaludis X1].
344210386       -                               171     archaea>euryarchaeota                                   Haloarcula hispanica ATCC 33960                                                 hypothetical protein HAH_0085 [Haloarcula hispanica ATCC 33960].
432328590       AciM339_0687                    171     archaea>euryarchaeota                                   Aciduliprofundum sp. MAR08-339                                                  hypothetical protein AciM339_0687 [Aciduliprofundum sp. MAR08-339].
490131057       -                               171     archaea>euryarchaeota                                   Methanobacterium formicicum                                                     ribosome biogenesis protein MJ0708 [Methanobacterium formicicum].
490650990       -                               171     archaea>euryarchaeota                                   Haloarcula vallismortis                                                         hypothetical protein [Haloarcula vallismortis].
490727797       -                               171     archaea>euryarchaeota                                   Haloarcula japonica                                                             hypothetical protein [Haloarcula japonica].
491098918       -                               171     archaea>euryarchaeota                                   Haloarcula sinaiiensis                                                          hypothetical protein [Haloarcula sinaiiensis].
491680547       -                               171     archaea>euryarchaeota                                   Haloarcula argentinensis                                                        hypothetical protein [Haloarcula argentinensis].
15678582        MTH554                          188     archaea>euryarchaeota                                   Methanothermobacter thermautotrophicus str. Delta H                             hypothetical protein MTH554 [Methanothermobacter thermautotrophicus str. Delta H].
494342197       -                               171     archaea>euryarchaeota                                   Haloarcula californiae                                                          hypothetical protein [Haloarcula californiae].
495582514       -                               171     archaea>euryarchaeota                                   Haloarcula amylolytica                                                          hypothetical protein [Haloarcula amylolytica].
495787619       -                               171     archaea>euryarchaeota                                   Methanobacterium sp. Maddingley MBC34                                           hypothetical protein [Methanobacterium sp. Maddingley MBC34].
495880133       -                               171     archaea>euryarchaeota                                   Thermoplasmatales archaeon SCGC AB-540-F20                                      hypothetical protein [Thermoplasmatales archaeon SCGC AB-540-F20].
497418908       -                               171     archaea>euryarchaeota                                   Halogranum salarium                                                             hypothetical protein [Halogranum salarium].
397781770       BN140_2604                      163     archaea>euryarchaeota                                   Methanoculleus bourgensis MS2                                                   hypothetical protein BN140_2604 [Methanoculleus bourgensis MS2].
374724640       MG2_1132                        189     archaea>euryarchaeota                                   uncultured marine group II euryarchaeote                                        hypothetical protein DUF367 [uncultured marine group II euryarchaeote].
282165453       MCP_2783                        170     archaea>euryarchaeota                                   Methanocella paludicola SANAE                                                   hypothetical protein MCP_2783 [Methanocella paludicola SANAE].
493716174       -                               190     archaea>euryarchaeota                                   Natrialba aegyptia                                                              hypothetical protein [Natrialba aegyptia].
307352334       -                               164     archaea>euryarchaeota                                   Methanoplanus petrolearius DSM 11571                                            hypothetical protein Mpet_0169 [Methanoplanus petrolearius DSM 11571].
474935103       TALC_01559                      170     archaea>euryarchaeota                                   Thermoplasmatales archaeon BRNA1                                                hypothetical protein TALC_01559 [Thermoplasmatales archaeon BRNA1].
491747519       -                               170     archaea>euryarchaeota                                   Natronobacterium gregoryi                                                       hypothetical protein [Natronobacterium gregoryi].
495435904       -                               170     archaea>euryarchaeota                                   Natronorubrum sulfidifaciens                                                    hypothetical protein [Natronorubrum sulfidifaciens].
20094076        MK0638                          169     archaea>euryarchaeota                                   Methanopyrus kandleri AV19                                                      hypothetical protein MK0638 [Methanopyrus kandleri AV19].
219853334       -                               164     archaea>euryarchaeota                                   Methanosphaerula palustris E1-9c                                                hypothetical protein Mpal_2788 [Methanosphaerula palustris E1-9c].
429191146       -                               190     archaea>euryarchaeota                                   Natronobacterium gregoryi SP2                                                   hypothetical protein Natgr_1148 [Natronobacterium gregoryi SP2].
297620086       Mvol_1563                       169     archaea>euryarchaeota                                   Methanococcus voltae A3                                                         hypothetical protein Mvol_1563 [Methanococcus voltae A3].
304314704       MTBMA_c09430                    169     archaea>euryarchaeota                                   Methanothermobacter marburgensis str. Marburg                                   hypothetical protein MTBMA_c09430 [Methanothermobacter marburgensis str. Marburg].
325959991       -                               169     archaea>euryarchaeota                                   Methanobacterium sp. AL-21                                                      hypothetical protein Metbo_2271 [Methanobacterium sp. AL-21].
383320891       Mtc_2489                        169     archaea>euryarchaeota                                   Methanocella conradii HZ254                                                     hypothetical protein Mtc_2489 [Methanocella conradii HZ254].
397773450       NJ7G_1675                       169     archaea>euryarchaeota                                   Natrinema sp. J7-2                                                              hypothetical protein NJ7G_1675 [Natrinema sp. J7-2].
435847555       -                               169     archaea>euryarchaeota                                   Natronococcus occultus SP4                                                      hypothetical protein Natoc_2237 [Natronococcus occultus SP4].
493194747       -                               169     archaea>euryarchaeota                                   Natrinema pallidum                                                              hypothetical protein [Natrinema pallidum].
495695803       -                               169     archaea>euryarchaeota                                   Natronococcus jeotgali                                                          hypothetical protein [Natronococcus jeotgali].
495729169       -                               169     archaea>euryarchaeota                                   Natrinema gari                                                                  hypothetical protein [Natrinema gari].
48477633        PTO0561                         168     archaea>euryarchaeota                                   Picrophilus torridus DSM 9790                                                   hypothetical protein PTO0561 [Picrophilus torridus DSM 9790].
124485051       Mlab_0224                       168     archaea>euryarchaeota                                   Methanocorpusculum labreanum Z                                                  hypothetical protein Mlab_0224 [Methanocorpusculum labreanum Z].
289596032       Aboo_0355                       168     archaea>euryarchaeota                                   Aciduliprofundum boonei T469                                                    Protein of unknown function DUF367 [Aciduliprofundum boonei T469].
294495848       Mmah_1192                       168     archaea>euryarchaeota                                   Methanohalophilus mahii DSM 5219                                                hypothetical protein Mmah_1192 [Methanohalophilus mahii DSM 5219].
55379416        -                               148     archaea>euryarchaeota                                   Haloarcula marismortui ATCC 43049                                               hypothetical protein rrnAC2804 [Haloarcula marismortui ATCC 43049].
88603648        -                               164     archaea>euryarchaeota                                   Methanospirillum hungatei JF-1                                                  hypothetical protein Mhun_2405 [Methanospirillum hungatei JF-1].
433590349       -                               168     archaea>euryarchaeota                                   Natrinema pellirubrum DSM 15624                                                 hypothetical protein Natpe_1029 [Natrinema pellirubrum DSM 15624].
490727487       -                               168     archaea>euryarchaeota                                   Methanocaldococcus villosus                                                     hypothetical protein [Methanocaldococcus villosus].
493699777       -                               168     archaea>euryarchaeota                                   Haloterrigena thermotolerans                                                    hypothetical protein [Haloterrigena thermotolerans].
495359898       -                               168     archaea>euryarchaeota                                   Aciduliprofundum boonei                                                         hypothetical protein [Aciduliprofundum boonei].
13540907        TVN0076                         164     archaea>euryarchaeota                                   Thermoplasma volcanium GSS1                                                     hypothetical protein TVN0076 [Thermoplasma volcanium GSS1].
76801499        NP1700A                         167     archaea>euryarchaeota                                   Natronomonas pharaonis DSM 2160                                                 hypothetical protein NP1700A [Natronomonas pharaonis DSM 2160].
222480096       -                               167     archaea>euryarchaeota                                   Halorubrum lacusprofundi ATCC 49239                                             hypothetical protein Hlac_1678 [Halorubrum lacusprofundi ATCC 49239].
256811023       Mefer_1081                      167     archaea>euryarchaeota                                   Methanocaldococcus fervens AG86                                                 hypothetical protein Mefer_1081 [Methanocaldococcus fervens AG86].
284166419       -                               167     archaea>euryarchaeota                                   Haloterrigena turkmenica DSM 5511                                               hypothetical protein Htur_3160 [Haloterrigena turkmenica DSM 5511].
300710204       HacjB3_04175                    167     archaea>euryarchaeota                                   Halalkalicoccus jeotgali B3                                                     hypothetical protein HacjB3_04175 [Halalkalicoccus jeotgali B3].
495883716       -                               165     archaea>euryarchaeota                                   Haloferax sp. BAB2207                                                           hypothetical protein [Haloferax sp. BAB2207].
386000751       Mhar_0034                       167     archaea>euryarchaeota                                   Methanosaeta harundinacea 6Ac                                                   hypothetical protein Mhar_0034 [Methanosaeta harundinacea 6Ac].
410670050       Mpsy_0844                       145     archaea>euryarchaeota                                   Methanolobus psychrophilus R15                                                  hypothetical protein Mpsy_0844 [Methanolobus psychrophilus R15].
490146031       -                               167     archaea>euryarchaeota                                   Halorubrum saccharovorum                                                        hypothetical protein [Halorubrum saccharovorum].
490155335       -                               167     archaea>euryarchaeota                                   Halococcus morrhuae                                                             hypothetical protein [Halococcus morrhuae].
490735652       -                               167     archaea>euryarchaeota                                   Halorubrum                                                                      hypothetical protein [Halorubrum].
493052775       -                               167     archaea>euryarchaeota                                   Halorubrum coriense                                                             hypothetical protein [Halorubrum coriense].
493476171       -                               167     archaea>euryarchaeota                                   Natrinema versiforme                                                            hypothetical protein [Natrinema versiforme].
493677691       -                               167     archaea>euryarchaeota                                   Halorubrum tebenquichense                                                       hypothetical protein [Halorubrum tebenquichense].
494587387       -                               167     archaea>euryarchaeota                                   Halorubrum                                                                      hypothetical protein [Halorubrum].
494965446       -                               167     archaea>euryarchaeota                                   Halococcus hamelinensis                                                         hypothetical protein [Halococcus hamelinensis].
495014166       -                               167     archaea>euryarchaeota                                   Halococcus thailandensis                                                        hypothetical protein [Halococcus thailandensis].
495251824       -                               167     archaea>euryarchaeota                                   Haladaptatus paucihalophilus                                                    hypothetical protein [Haladaptatus paucihalophilus].
495275391       -                               167     archaea>euryarchaeota                                   Halorubrum aidingense                                                           hypothetical protein [Halorubrum aidingense].
495277937       -                               167     archaea>euryarchaeota                                   Halorubrum lipolyticum                                                          hypothetical protein [Halorubrum lipolyticum].
495289343       -                               167     archaea>euryarchaeota                                   Haloterrigena limicola                                                          hypothetical protein [Haloterrigena limicola].
495719343       -                               167     archaea>euryarchaeota                                   Halorubrum californiense                                                        hypothetical protein [Halorubrum californiense].
495858456       -                               167     archaea>euryarchaeota                                   Halorubrum hochstenium                                                          hypothetical protein [Halorubrum hochstenium].
496123929       -                               167     archaea>euryarchaeota                                   Halorubrum kocurii                                                              hypothetical protein [Halorubrum kocurii].
496169427       -                               167     archaea>euryarchaeota                                   Haloterrigena salina                                                            hypothetical protein [Haloterrigena salina].
495850307       -                               165     archaea>euryarchaeota                                   Haloferax                                                                       hypothetical protein [Haloferax].
495602068       -                               165     archaea>euryarchaeota                                   Haloferax elongans                                                              hypothetical protein [Haloferax elongans].
257052398       -                               166     archaea>euryarchaeota                                   Halorhabdus utahensis DSM 12940                                                 hypothetical protein Huta_1322 [Halorhabdus utahensis DSM 12940].
495594366       -                               165     archaea>euryarchaeota                                   Haloferax mucosum                                                               hypothetical protein [Haloferax mucosum].
433639509       -                               166     archaea>euryarchaeota                                   Halovivax ruber XH-70                                                           hypothetical protein Halru_2555 [Halovivax ruber XH-70].
452206912       Nmlp_2112                       166     archaea>euryarchaeota                                   Natronomonas moolapensis 8.8.11                                                 UPF0293 family protein [Natronomonas moolapensis 8.8.11].
494976606       -                               166     archaea>euryarchaeota                                   Halovivax asiaticus                                                             hypothetical protein [Halovivax asiaticus].
495371747       -                               165     archaea>euryarchaeota                                   Haloferax prahovense                                                            hypothetical protein [Haloferax prahovense].
494806325       -                               165     archaea>euryarchaeota                                   Haloferax larsenii                                                              hypothetical protein [Haloferax larsenii].
491117889       -                               165     archaea>euryarchaeota                                   Haloferax gibbonsii                                                             hypothetical protein [Haloferax gibbonsii].
333911612       Metig_1748                      171     archaea>euryarchaeota                                   Methanotorris igneus Kol 5                                                      hypothetical protein [Methanotorris igneus Kol 5].
15790920        VNG2075C                        193     archaea>euryarchaeota                                   Halobacterium sp. NRC-1                                                         hypothetical protein VNG2075C [Halobacterium sp. NRC-1].
491108053       -                               165     archaea>euryarchaeota                                   Haloferax                                                                       hypothetical protein [Haloferax].
169236665       OE3895F                         165     archaea>euryarchaeota                                   Halobacterium salinarum R1                                                      hypothetical protein OE3895F [Halobacterium salinarum R1].
292656052       HVO_1923                        165     archaea>euryarchaeota                                   Haloferax volcanii DS2                                                          hypothetical protein HVO_1923 [Haloferax volcanii DS2].
389847456       HFX_2016                        165     archaea>euryarchaeota                                   Haloferax mediterranei ATCC 33500                                               hypothetical protein HFX_2016 [Haloferax mediterranei ATCC 33500].
490137000       -                               165     archaea>euryarchaeota                                   Methanofollis liminatans                                                        hypothetical protein [Methanofollis liminatans].
493158293       -                               171     archaea>euryarchaeota                                   Natrialba chahannaoensis                                                        hypothetical protein [Natrialba chahannaoensis].
70606531        Saci_0730                       165     archaea>crenarchaeota                                   Sulfolobus acidocaldarius DSM 639                                               hypothetical protein Saci_0730 [Sulfolobus acidocaldarius DSM 639].
330835894       Mcup_2037                       148     archaea>crenarchaeota                                   Metallosphaera cuprina Ar-4                                                     hypothetical protein Mcup_2037 [Metallosphaera cuprina Ar-4].
347522711       -                               195     archaea>crenarchaeota                                   Pyrolobus fumarii 1A                                                            hypothetical protein Pyrfu_0157 [Pyrolobus fumarii 1A].
325968641       VMUT_1126                       186     archaea>crenarchaeota                                   Vulcanisaeta moutnovskia 768-28                                                 hypothetical protein VMUT_1126 [Vulcanisaeta moutnovskia 768-28].
385773539       SiH_1543                        166     archaea>crenarchaeota                                   Sulfolobus islandicus HVE10/4                                                   hypothetical protein [Sulfolobus islandicus HVE10/4].
229585114       M1627_1693                      166     archaea>crenarchaeota                                   Sulfolobus islandicus M.16.27                                                   hypothetical protein M1627_1693 [Sulfolobus islandicus M.16.27].
227827844       M1425_1577                      166     archaea>crenarchaeota                                   Sulfolobus islandicus M.14.25                                                   hypothetical protein M1425_1577 [Sulfolobus islandicus M.14.25].
15897473        -                               208     archaea>crenarchaeota                                   Sulfolobus solfataricus P2                                                      hypothetical protein SSO0551 [Sulfolobus solfataricus P2].
496364632       -                               165     archaea>crenarchaeota                                   Metallosphaera yellowstonensis                                                  hypothetical protein [Metallosphaera yellowstonensis].
15920504        ST0311                          167     archaea>crenarchaeota                                   Sulfolobus tokodaii str. 7                                                      hypothetical protein ST0311 [Sulfolobus tokodaii str. 7].
296242958       Tagg_1227                       153     archaea>crenarchaeota                                   Thermosphaera aggregans DSM 11486                                               hypothetical protein Tagg_1227 [Thermosphaera aggregans DSM 11486].
146305039       -                               169     archaea>crenarchaeota                                   Metallosphaera sedula DSM 5348                                                  hypothetical protein Msed_2294 [Metallosphaera sedula DSM 5348].
389860573       TCELL_0249                      170     archaea>crenarchaeota                                   Thermogladius cellulolyticus 1633                                               hypothetical protein TCELL_0249 [Thermogladius cellulolyticus 1633].
390939041       Desfe_1337                      186     archaea>crenarchaeota                                   Desulfurococcus fermentans DSM 16532                                            hypothetical protein Desfe_1337 [Desulfurococcus fermentans DSM 16532].
307594553       -                               185     archaea>crenarchaeota                                   Vulcanisaeta distributa DSM 14429                                               hypothetical protein Vdis_0418 [Vulcanisaeta distributa DSM 14429].
320101415       Desmu_1229                      173     archaea>crenarchaeota                                   Desulfurococcus mucosus DSM 2162                                                hypothetical protein [Desulfurococcus mucosus DSM 2162].
126465853       Smar_0955                       195     archaea>crenarchaeota                                   Staphylothermus marinus F1                                                      hypothetical protein Smar_0955 [Staphylothermus marinus F1].
385805852       FFONT_0810                      183     archaea>crenarchaeota                                   Fervidicoccus fontis Kam940                                                     hypothetical protein FFONT_0810 [Fervidicoccus fontis Kam940].
218884546       DKAM_1235                       174     archaea>crenarchaeota                                   Desulfurococcus kamchatkensis 1221n                                             hypothetical protein DKAM_1235 [Desulfurococcus kamchatkensis 1221n].
124026914       Hbut_0010                       197     archaea>crenarchaeota                                   Hyperthermus butylicus DSM 5456                                                 hypothetical protein Hbut_0010 [Hyperthermus butylicus DSM 5456].
305662498       -                               191     archaea>crenarchaeota                                   Ignisphaera aggregans DSM 17230                                                 hypothetical protein [Ignisphaera aggregans DSM 17230].
297527474       Shell_1511                      178     archaea>crenarchaeota                                   Staphylothermus hellenicus DSM 12710                                            hypothetical protein Shell_1511 [Staphylothermus hellenicus DSM 12710].
374857463       HGMM_F45C05C30                  196     archaea>crenarchaeota                                   uncultured crenarchaeote                                                        hypothetical conserved protein [uncultured crenarchaeote].
332796467       Ahos_0783                       147     archaea>crenarchaeota                                   Acidianus hospitalis W1                                                         hypothetical protein Ahos_0783 [Acidianus hospitalis W1].
407464501       NSED_03175                      163     archaea                                                 Candidatus Nitrosopumilus sp. AR2                                               hypothetical protein NSED_03175 [Candidatus Nitrosopumilus sp. AR2].
161528125       Nmar_0617                       163     archaea                                                 Nitrosopumilus maritimus SCM1                                                   hypothetical protein Nmar_0617 [Nitrosopumilus maritimus SCM1].
345005908       -                               182     archaea                                                 halophilic archaeon DL31                                                        hypothetical protein [halophilic archaeon DL31].
52550181        GZ35D7_15                       163     archaea                                                 uncultured archaeon GZfos35D7                                                   RNase P RNA component [uncultured archaeon GZfos35D7].
268323393       BSM_04580                       173     archaea                                                 uncultured archaeon                                                             conserved hypothetical protein containing DUF367 domain [uncultured archaeon].
494814693       -                               164     archaea                                                 Candidatus Nitrosoarchaeum koreensis                                            ribonuclease P [Candidatus Nitrosoarchaeum koreensis].
167042572       ALOHA_HF4000ANIW133I6ctg1g12    159     archaea                                                 uncultured marine crenarchaeote HF4000_ANIW133I6                                putative domain of unknown function (DUF367) [uncultured marine crenarchaeote HF4000_ANIW133I6].
497881530       -                               159     archaea                                                 Candidatus Nitrosoarchaeum limnia                                               ribonuclease P [Candidatus Nitrosoarchaeum limnia].
497880326       -                               158     archaea                                                 Candidatus Nitrosoarchaeum limnia                                               ribonuclease P [Candidatus Nitrosoarchaeum limnia].
118576405       CENSYa_1220                     172     archaea                                                 Cenarchaeum symbiosum A                                                         hypothetical protein CENSYa_1220 [Cenarchaeum symbiosum A].
408404008       Ngar_c13970                     168     archaea                                                 Candidatus Nitrososphaera gargensis Ga9.2                                       hypothetical protein Ngar_c13970 [Candidatus Nitrososphaera gargensis Ga9.2].
495574528       -                               166     archaea                                                 Candidatus Nitrosopumilus salaria                                               ribonuclease P [Candidatus Nitrosopumilus salaria].
14548153        -                               165     archaea                                                 uncultured crenarchaeote 4B7                                                    uncharacterized conserved protein [uncultured crenarchaeote 4B7].
494643385       -                               165     archaea                                                 Candidatus Nitrosoarchaeum limnia                                               ribonuclease P [Candidatus Nitrosoarchaeum limnia].
407462150       NKOR_03155                      163     archaea                                                 Candidatus Nitrosopumilus koreensis AR1                                         hypothetical protein NKOR_03155 [Candidatus Nitrosopumilus koreensis AR1].
167042146       ALOHA_HF4000ANIW93E5ctg7g24     165     archaea                                                 uncultured marine crenarchaeote HF4000_ANIW93E5                                 putative domain of unknown function (DUF367) [uncultured marine crenarchaeote HF4000_ANIW93E5].
167043417       ALOHA_HF4000APKG1C9ctg2g22      181                                                             uncultured marine microorganism HF4000_APKG1C9                                  putative domain of unknown function (DUF367) [uncultured marine microorganism HF4000_APKG1C9].
#;TDD+insert+TDD
221483840       TGGT1_016140                    586     eukaryota>alveolata>apicomplexa                         Toxoplasma gondii GT1                                                           conserved hypothetical protein [Toxoplasma gondii GT1].
221505127       TGVEG_044680                    586     eukaryota>alveolata>apicomplexa                         Toxoplasma gondii VEG                                                           conserved hypothetical protein [Toxoplasma gondii VEG].
237836857       TGME49_004540                   586     eukaryota>alveolata>apicomplexa                         Toxoplasma gondii ME49                                                          hypothetical protein TGME49_004540 [Toxoplasma gondii ME49].
401405733       NCLIV_020710                    579     eukaryota>alveolata>apicomplexa                         Neospora caninum Liverpool                                                      hypothetical protein NCLIV_020710 [Neospora caninum Liverpool].
#;TDD+Noc2
484856238       NBO_41g0010                     399     eukaryota>fungi>microsporidia                           Nosema bombycis CQ1                                                             hypothetical protein NBO_41g0010, partial [Nosema bombycis CQ1].
#;TDD+STYKIN
25396009        -                               605     eukaryota>metazoa>nematoda                              Caenorhabditis elegans                                                          protein F52C12.2 [imported] - Caenorhabditis elegans.
268553847       CBG22508                        556     eukaryota>metazoa>nematoda                              Caenorhabditis briggsae                                                         Hypothetical protein CBG22508 [Caenorhabditis briggsae].
#;proteins containing TDD with likely spurious domain fusions or fusions to regions of low complexity
459373764       G210_0225                       1579    eukaryota>fungi>ascomycota                              Candida maltosa Xu316                                                           hypothetical protein G210_0225, partial [Candida maltosa Xu316].
358054597       E5Q_06224                       1239    eukaryota>fungi>basidiomycota                           Mixia osmundae IAM 14324                                                        hypothetical protein E5Q_06224 [Mixia osmundae IAM 14324].
443924131       AG1IA_02765                     1154    eukaryota>fungi>basidiomycota                           Rhizoctonia solani AG-1 IA                                                      DUF367 family protein [Rhizoctonia solani AG-1 IA].
403412301       FIBRA_01009                     743     eukaryota>fungi>basidiomycota                           Fibroporia radiculosa                                                           predicted protein [Fibroporia radiculosa].
118353061       TTHERM_00160710                 694     eukaryota>alveolata>ciliophora                          Tetrahymena thermophila                                                         hypothetical protein TTHERM_00160710 [Tetrahymena thermophila].
339241697       Tsp_00957                       694     eukaryota>metazoa>nematoda                              Trichinella spiralis                                                            conserved hypothetical protein [Trichinella spiralis].
380012742       LOC100869694                    662     eukaryota>metazoa>hexapoda                              Apis florea                                                                     PREDICTED: uncharacterized protein LOC100869694 [Apis florea].
405974122       CGI_10026589                    571     eukaryota>metazoa>mollusca                              Crassostrea gigas                                                               UPF0293 protein C16orf42-like protein [Crassostrea gigas].
358336209       CLF_105394                      501     eukaryota>metazoa                                       Clonorchis sinensis                                                             pre-rRNA-processing protein TSR3 [Clonorchis sinensis].

2. pc1599 family

#;TDD
498274327       -                               235     bacteria>planctomycetes                                 Schlesneria paludicola                                                          hypothetical protein [Schlesneria paludicola].
492730403       -                               215     bacteria>actinobacteria                                 Gordonia hirsuta                                                                hypothetical protein [Gordonia hirsuta].
46447233        pc1599                          178     bacteria>chlamydiae                                     Candidatus Protochlamydia amoebophila UWE25                                     hypothetical protein pc1599 [Candidatus Protochlamydia amoebophila UWE25].
297621044       wcw_0808                        162     bacteria>chlamydiae                                     Waddlia chondrophila WSU 86-1044                                                hypothetical protein wcw_0808 [Waddlia chondrophila WSU 86-1044].
325108807       -                               162     bacteria>planctomycetes                                 Planctomyces brasiliensis DSM 5305                                              hypothetical protein Plabr_2251 [Planctomyces brasiliensis DSM 5305].
338175900       PUV_19060                       159     bacteria>chlamydiae                                     Parachlamydia acanthamoebae UV-7                                                hypothetical protein PUV_19060 [Parachlamydia acanthamoebae UV-7].
379335141       -                               156     bacteria                                                uncultured bacterium W5-77b                                                     hypothetical protein [uncultured bacterium W5-77b].
338733735       SNE_A18400                      152     bacteria>chlamydiae                                     Simkania negevensis Z                                                           hypothetical protein SNE_A18400 [Simkania negevensis Z].
488721742       -                               152     bacteria>planctomycetes                                 Planctomyces maris                                                              hypothetical protein [Planctomyces maris].
297180515       -                               151     bacteria>verrucomicrobia                                uncultured Verrucomicrobiales bacterium HF0010_05E02                            hypothetical protein [uncultured Verrucomicrobiales bacterium HF0010_05E02].
294055331       Caka_1800                       150     bacteria>verrucomicrobia                                Coraliomargarita akajimensis DSM 45221                                          hypothetical protein [Coraliomargarita akajimensis DSM 45221].
406987877       ACD_17C00361G0002               148     bacteria                                                uncultured bacterium                                                            hypothetical protein ACD_17C00361G0002 [uncultured bacterium].

3. DTWD2 family

#;zinc ribbon+TDD
378954064       -                               226     proteobacteria>gammaproteobacteria                      Salmonella enterica subsp. enterica serovar Gallinarum/pullorum                 hypothetical protein SPUL_0275 [Salmonella enterica subsp. enterica serovar Gallinarum/pullorum str. RKS5078].
384245671       COCSUDRAFT_54759                308     eukaryota>viridiplantae>chlorophyta                     Coccomyxa subellipsoidea C-169                                                  DTW-domain-containing protein [Coccomyxa subellipsoidea C-169].
226496455       LOC100280375                    262     eukaryota>viridiplantae                                 Zea mays                                                                        hypothetical protein [Zea mays].
225441854       LOC100250284                    254     eukaryota>viridiplantae                                 Vitis vinifera                                                                  PREDICTED: DTW domain-containing protein 2-like [Vitis vinifera].
474370806       TRIUR3_10924                    195     eukaryota>viridiplantae                                 Triticum urartu                                                                 hypothetical protein TRIUR3_10924 [Triticum urartu].
242082572       SORBIDRAFT_08g001110            263     eukaryota>viridiplantae                                 Sorghum bicolor                                                                 hypothetical protein SORBIDRAFT_08g001110 [Sorghum bicolor].
460372871       LOC101261564                    263     eukaryota>viridiplantae                                 Solanum lycopersicum                                                            PREDICTED: DTW domain-containing protein 2-like [Solanum lycopersicum].
302799836       SELMODRAFT_421162               226     eukaryota>viridiplantae                                 Selaginella moellendorffii                                                      hypothetical protein SELMODRAFT_421162 [Selaginella moellendorffii].
255577459       RCOM_0749500                    249     eukaryota>viridiplantae                                 Ricinus communis                                                                conserved hypothetical protein [Ricinus communis].
462400352       PRUPE_ppb019012mg               300     eukaryota>viridiplantae                                 Prunus persica                                                                  hypothetical protein PRUPE_ppb019012mg [Prunus persica].
224087020       POPTRDRAFT_560511               247     eukaryota>viridiplantae                                 Populus trichocarpa                                                             predicted protein [Populus trichocarpa].
222870321       POPTRDRAFT_580176               345     eukaryota>viridiplantae                                 Populus trichocarpa                                                             predicted protein [Populus trichocarpa].
294460758       -                               264     eukaryota>viridiplantae                                 Picea sitchensis                                                                unknown [Picea sitchensis].
168043423       PHYPADRAFT_17594                227     eukaryota>viridiplantae                                 Physcomitrella patens subsp. patens                                             predicted protein, partial [Physcomitrella patens subsp. patens].
115484133       Os11g0145100                    256     eukaryota>viridiplantae                                 Oryza sativa Japonica Group                                                     Os11g0145100 [Oryza sativa Japonica Group].
115487290       Os12g0142600                    259     eukaryota>viridiplantae                                 Oryza sativa Japonica Group                                                     Os12g0142600 [Oryza sativa Japonica Group].
346703772       Ogl12g0089G06_3                 259     eukaryota>viridiplantae                                 Oryza glaberrima                                                                hypothetical_protein [Oryza glaberrima].
346703385       Ogl11g0039H06_13                256     eukaryota>viridiplantae                                 Oryza glaberrima                                                                DTW domain containing protein, expressed [Oryza glaberrima].
346703196       Ob11g0082C11_15                 264     eukaryota>viridiplantae                                 Oryza brachyantha                                                               hypothetical_protein [Oryza brachyantha].
326504812       -                               259     eukaryota>viridiplantae                                 Hordeum vulgare subsp. vulgare                                                  predicted protein [Hordeum vulgare subsp. vulgare].
356503444       LOC100816233                    242     eukaryota>viridiplantae                                 Glycine max                                                                     PREDICTED: DTW domain-containing protein 2-like [Glycine max].
449463356       LOC101214345                    246     eukaryota>viridiplantae                                 Cucumis sativus                                                                 PREDICTED: DTW domain-containing protein 2-like [Cucumis sativus].
449510541       LOC101214345                    246     eukaryota>viridiplantae                                 Cucumis sativus                                                                 PREDICTED: LOW QUALITY PROTEIN: DTW domain-containing protein 2-like [Cucumis sativus].
502108802       LOC101507793                    261     eukaryota>viridiplantae                                 Cicer arietinum                                                                 PREDICTED: DTW domain-containing protein 2-like [Cicer arietinum].
357160984       LOC100829855                    264     eukaryota>viridiplantae                                 Brachypodium distachyon                                                         PREDICTED: DTW domain-containing protein 2-like isoform 1 [Brachypodium distachyon].
15227419        AT2G41750                       253     eukaryota>viridiplantae                                 Arabidopsis thaliana                                                            DTW domain-containing protein [Arabidopsis thaliana].
297827825       ARALYDRAFT_903514               251     eukaryota>viridiplantae                                 Arabidopsis lyrata subsp. lyrata                                                DTW domain-containing protein [Arabidopsis lyrata subsp. lyrata].
219120320       PHATRDRAFT_36582                154     eukaryota>stramenopiles                                 Phaeodactylum tricornutum CCAP 1055/1                                           predicted protein [Phaeodactylum tricornutum CCAP 1055/1].
223994981       THAPSDRAFT_268179               268     eukaryota>stramenopiles                                 Thalassiosira pseudonana CCMP1335                                               hypothetical protein THAPSDRAFT_268179 [Thalassiosira pseudonana CCMP1335].
397625412       THAOC_11135                     178     eukaryota>stramenopiles                                 Thalassiosira oceanica                                                          hypothetical protein THAOC_11135 [Thalassiosira oceanica].
405977352       CGI_10026427                    214     eukaryota>metazoa>mollusca                              Crassostrea gigas                                                               DTW domain-containing protein 2 [Crassostrea gigas].
91076622        LOC658098                       247     eukaryota>metazoa>hexapoda                              Tribolium castaneum                                                             PREDICTED: similar to CG10050 CG10050-PA [Tribolium castaneum].
242022550       Phum_PHUM541180                 248     eukaryota>metazoa>hexapoda                              Pediculus humanus corporis                                                      conserved hypothetical protein [Pediculus humanus corporis].
345495555       LOC100120911                    253     eukaryota>metazoa>hexapoda                              Nasonia vitripennis                                                             PREDICTED: DTW domain-containing protein 2-like [Nasonia vitripennis].
383852991       LOC100878333                    252     eukaryota>metazoa>hexapoda                              Megachile rotundata                                                             PREDICTED: DTW domain-containing protein 2-like [Megachile rotundata].
121543632       -                               279     eukaryota>metazoa>hexapoda                              Maconellicoccus hirsutus                                                        hypothetical protein [Maconellicoccus hirsutus].
307196320       EAI_00975                       253     eukaryota>metazoa>hexapoda                              Harpegnathos saltator                                                           DTW domain-containing protein 2 [Harpegnathos saltator].
195498789       Dyak_GE24916                    251     eukaryota>metazoa>hexapoda                              Drosophila yakuba                                                               GE24916 [Drosophila yakuba].
195445465       Dwil_GK12004                    258     eukaryota>metazoa>hexapoda                              Drosophila willistoni                                                           GK12004 [Drosophila willistoni].
195395568       Dvir_GJ10931                    264     eukaryota>metazoa>hexapoda                              Drosophila virilis                                                              GJ10931 [Drosophila virilis].
195568930       Dsim_GD19925                    251     eukaryota>metazoa>hexapoda                              Drosophila simulans                                                             GD19925 [Drosophila simulans].
195344189       Dsec_GM10945                    251     eukaryota>metazoa>hexapoda                              Drosophila sechellia                                                            GM10945 [Drosophila sechellia].
195157236       Dper_GL12433                    252     eukaryota>metazoa>hexapoda                              Drosophila persimilis                                                           GL12433 [Drosophila persimilis].
195108217       Dmoj_GI24108                    265     eukaryota>metazoa>hexapoda                              Drosophila mojavensis                                                           GI24108 [Drosophila mojavensis].
225581135       GA10033                         252     eukaryota>metazoa>hexapoda                              Drosophila miranda                                                              GA10033 [Drosophila miranda].
21358337        Dmel_CG10050                    251     eukaryota>metazoa>hexapoda                              Drosophila melanogaster                                                         CG10050 [Drosophila melanogaster].
157816604       CG10050                         269     eukaryota>metazoa>hexapoda                              Drosophila melanogaster                                                         IP19844p [Drosophila melanogaster].
195037761       Dgri_GH18297                    261     eukaryota>metazoa>hexapoda                              Drosophila grimshawi                                                            GH18297 [Drosophila grimshawi].
194899241       Dere_GG13963                    251     eukaryota>metazoa>hexapoda                              Drosophila erecta                                                               GG13963 [Drosophila erecta].
194741514       Dana_GF17666                    260     eukaryota>metazoa>hexapoda                              Drosophila ananassae                                                            GF17666 [Drosophila ananassae].
478255488       YQE_07666                       251     eukaryota>metazoa>hexapoda                              Dendroctonus ponderosae                                                         hypothetical protein YQE_07666, partial [Dendroctonus ponderosae].
357611226       KGM_13835                       249     eukaryota>metazoa>hexapoda                              Danaus plexippus                                                                hypothetical protein KGM_13835 [Danaus plexippus].
170056885       CpipJ_CPIJ013359                256     eukaryota>metazoa>hexapoda                              Culex quinquefasciatus                                                          conserved hypothetical protein [Culex quinquefasciatus].
498980164       LOC101453877                    278     eukaryota>metazoa>hexapoda                              Ceratitis capitata                                                              PREDICTED: DTW domain-containing protein 2-like isoform X2 [Ceratitis capitata].
498980160       LOC101453877                    288     eukaryota>metazoa>hexapoda                              Ceratitis capitata                                                              PREDICTED: DTW domain-containing protein 2-like isoform X1 [Ceratitis capitata].
498992661       LOC101459306                    232     eukaryota>metazoa>hexapoda                              Ceratitis capitata                                                              PREDICTED: DTW domain-containing protein YfiP-like [Ceratitis capitata].
307167212       EAG_06027                       253     eukaryota>metazoa>hexapoda                              Camponotus floridanus                                                           DTW domain-containing protein 2 [Camponotus floridanus].
340716788       LOC100642727                    252     eukaryota>metazoa>hexapoda                              Bombus terrestris                                                               PREDICTED: DTW domain-containing protein 2-like [Bombus terrestris].
350421855       LOC100742445                    252     eukaryota>metazoa>hexapoda                              Bombus impatiens                                                                PREDICTED: DTW domain-containing protein 2-like [Bombus impatiens].
66512549        LOC411215                       252     eukaryota>metazoa>hexapoda                              Apis mellifera                                                                  PREDICTED: DTW domain-containing protein 2-like isoform 1 [Apis mellifera].
380018034       LOC100868081                    252     eukaryota>metazoa>hexapoda                              Apis florea                                                                     PREDICTED: DTW domain-containing protein 2-like [Apis florea].
158301587       AgaP_AGAP001801                 265     eukaryota>metazoa>hexapoda                              Anopheles gambiae str. PEST                                                     AGAP001801-PA [Anopheles gambiae str. PEST].
312374850       AND_15436                       230     eukaryota>metazoa>hexapoda                              Anopheles darlingi                                                              hypothetical protein AND_15436 [Anopheles darlingi].
157131809       AaeL_AAEL002760                 262     eukaryota>metazoa>hexapoda                              Aedes aegypti                                                                   hypothetical protein AaeL_AAEL002760 [Aedes aegypti].
193603468       LOC100166158                    249     eukaryota>metazoa>hexapoda                              Acyrthosiphon pisum                                                             PREDICTED: DTW domain-containing protein 2-like [Acyrthosiphon pisum].
332027188       G5I_04127                       265     eukaryota>metazoa>hexapoda                              Acromyrmex echinatior                                                           DTW domain-containing protein 2 [Acromyrmex echinatior].
291221637       LOC100369401                    249     eukaryota>metazoa>hemichordata                          Saccoglossus kowalevskii                                                        PREDICTED: CG10050-like [Saccoglossus kowalevskii].
390343620       LOC588342                       268     eukaryota>metazoa>echinodermata                         Strongylocentrotus purpuratus                                                   PREDICTED: DTW domain-containing protein 2-like [Strongylocentrotus purpuratus].
321478474       DAPPUDRAFT_5534                 228     eukaryota>metazoa>crustacea                             Daphnia pulex                                                                   hypothetical protein DAPPUDRAFT_5534, partial [Daphnia pulex].
156387783       NEMVEDRAFT_v1g101190            237     eukaryota>metazoa>cnidaria                              Nematostella vectensis                                                          predicted protein [Nematostella vectensis].
432884713       LOC101154881                    293     eukaryota>metazoa>chordata>vertebrata>actinopterygii    Oryzias latipes                                                                 PREDICTED: DTW domain-containing protein 2-like [Oryzias latipes].
498981159       LOC101474204                    286     eukaryota>metazoa>chordata>vertebrata>actinopterygii    Maylandia zebra                                                                 PREDICTED: DTW domain-containing protein 2-like isoform X1 [Maylandia zebra].
348542304       LOC100706969                    286     eukaryota>metazoa>chordata>vertebrata>actinopterygii    Oreochromis niloticus                                                           PREDICTED: DTW domain-containing protein 2-like [Oreochromis niloticus].
498981163       LOC101474204                    273     eukaryota>metazoa>chordata>vertebrata>actinopterygii    Maylandia zebra                                                                 PREDICTED: DTW domain-containing protein 2-like isoform X2 [Maylandia zebra].
326677476       LOC100537837                    285     eukaryota>metazoa>chordata>vertebrata>actinopterygii    Danio rerio                                                                     PREDICTED: DTW domain-containing protein 2-like [Danio rerio].
47228430        GSTEN:00025216:G:001            229     eukaryota>metazoa>chordata>vertebrata>actinopterygii    Tetraodon nigroviridis                                                          unnamed protein product, partial [Tetraodon nigroviridis].
301759293       LOC100472492                    298     eukaryota>metazoa>chordata>vertebrata                   Ailuropoda melanoleuca                                                          PREDICTED: DTW domain-containing protein 2-like [Ailuropoda melanoleuca].
483505042       Anapl_15864                     226     eukaryota>metazoa>chordata>vertebrata                   Anas platyrhynchos                                                              DTW domain-containing protein 2, partial [Anas platyrhynchos].
327276595       LOC100558094                    282     eukaryota>metazoa>chordata>vertebrata                   Anolis carolinensis                                                             PREDICTED: DTW domain-containing protein 2-like [Anolis carolinensis].
440910090       M91_07284                       298     eukaryota>metazoa>chordata>vertebrata                   Bos mutus                                                                       DTW domain-containing protein 2 [Bos mutus].
329663612       DTWD2                           298     eukaryota>metazoa>chordata>vertebrata                   Bos taurus                                                                      DTW domain-containing protein 2 [Bos taurus].
296193901       DTWD2                           298     eukaryota>metazoa>chordata>vertebrata                   Callithrix jacchus                                                              PREDICTED: DTW domain-containing protein 2 [Callithrix jacchus].
345777734       LOC608488                       139     eukaryota>metazoa>chordata>vertebrata                   Canis lupus familiaris                                                          PREDICTED: DTW domain-containing protein 2-like [Canis lupus familiaris].
73970560        LOC608190                       319     eukaryota>metazoa>chordata>vertebrata                   Canis lupus familiaris                                                          PREDICTED: DTW domain-containing protein 2-like [Canis lupus familiaris].
348575105       Dtwd2                           298     eukaryota>metazoa>chordata>vertebrata                   Cavia porcellus                                                                 PREDICTED: DTW domain-containing protein 2 [Cavia porcellus].
478491951       DTWD2                           296     eukaryota>metazoa>chordata>vertebrata                   Ceratotherium simum simum                                                       PREDICTED: DTW domain-containing protein 2 [Ceratotherium simum simum].
449280201       A306_03751                      226     eukaryota>metazoa>chordata>vertebrata                   Columba livia                                                                   DTW domain-containing protein 2, partial [Columba livia].
488584769       DTWD2                           313     eukaryota>metazoa>chordata>vertebrata                   Dasypus novemcinctus                                                            PREDICTED: DTW domain-containing protein 2 [Dasypus novemcinctus].
194219996       DTWD2                           312     eukaryota>metazoa>chordata>vertebrata                   Equus caballus                                                                  PREDICTED: DTW domain-containing protein 2 [Equus caballus].
410948054       DTWD2                           275     eukaryota>metazoa>chordata>vertebrata                   Felis catus                                                                     PREDICTED: DTW domain-containing protein 2 [Felis catus].
71895351        DTWD2                           265     eukaryota>metazoa>chordata>vertebrata                   Gallus gallus                                                                   DTW domain-containing protein 2 [Gallus gallus].
426349733       LOC101130119                    243     eukaryota>metazoa>chordata>vertebrata                   Gorilla gorilla gorilla                                                         PREDICTED: DTW domain-containing protein 2-like, partial [Gorilla gorilla gorilla].
119569310       hCG_1646292                     232     eukaryota>metazoa>chordata>vertebrata                   Homo sapiens                                                                    DTW domain containing 2, isoform CRA_d [Homo sapiens].
27734779        DTWD2                           298     eukaryota>metazoa>chordata>vertebrata                   Homo sapiens                                                                    DTW domain-containing protein 2 [Homo sapiens].
344265496       LOC100659874                    269     eukaryota>metazoa>chordata>vertebrata                   Loxodonta africana                                                              PREDICTED: DTW domain-containing protein 2-like [Loxodonta africana].
355761951       EGM_20009                       226     eukaryota>metazoa>chordata>vertebrata                   Macaca fascicularis                                                             DTW domain-containing protein 2, partial [Macaca fascicularis].
386780911       DTWD2                           298     eukaryota>metazoa>chordata>vertebrata                   Macaca mulatta                                                                  DTW domain-containing protein 2 [Macaca mulatta].
126333840       LOC100028363                    304     eukaryota>metazoa>chordata>vertebrata                   Monodelphis domestica                                                           PREDICTED: DTW domain-containing protein 2-like [Monodelphis domestica].
110625694       Dtwd2                           298     eukaryota>metazoa>chordata>vertebrata                   Mus musculus                                                                    DTW domain-containing protein 2 isoform 1 [Mus musculus].
283837781       Dtwd2                           202     eukaryota>metazoa>chordata>vertebrata                   Mus musculus                                                                    DTW domain-containing protein 2 isoform 2 [Mus musculus].
432114660       MDA_GLEAN10021753               146     eukaryota>metazoa>chordata>vertebrata                   Myotis davidii                                                                  DTW domain-containing protein 2 [Myotis davidii].
332221530       DTWD2                           298     eukaryota>metazoa>chordata>vertebrata                   Nomascus leucogenys                                                             PREDICTED: DTW domain-containing protein 2 [Nomascus leucogenys].
472385439       DTWD2                           298     eukaryota>metazoa>chordata>vertebrata                   Odobenus rosmarus divergens                                                     PREDICTED: DTW domain-containing protein 2 [Odobenus rosmarus divergens].
465995867       DTWD2                           298     eukaryota>metazoa>chordata>vertebrata                   Orcinus orca                                                                    PREDICTED: DTW domain-containing protein 2 [Orcinus orca].
149412932       LOC100081959                    270     eukaryota>metazoa>chordata>vertebrata                   Ornithorhynchus anatinus                                                        PREDICTED: DTW domain-containing protein 2-like [Ornithorhynchus anatinus].
291387215       LOC100346002                    258     eukaryota>metazoa>chordata>vertebrata                   Oryctolagus cuniculus                                                           PREDICTED: DTW domain containing 2 [Oryctolagus cuniculus].
395831726       DTWD2                           298     eukaryota>metazoa>chordata>vertebrata                   Otolemur garnettii                                                              PREDICTED: DTW domain-containing protein 2 [Otolemur garnettii].
426229323       DTWD2                           298     eukaryota>metazoa>chordata>vertebrata                   Ovis aries                                                                      PREDICTED: DTW domain-containing protein 2 [Ovis aries].
397513001       LOC100986171                    372     eukaryota>metazoa>chordata>vertebrata                   Pan paniscus                                                                    PREDICTED: DTW domain-containing protein 2-like [Pan paniscus].
114601278       DTWD2                           298     eukaryota>metazoa>chordata>vertebrata                   Pan troglodytes                                                                 PREDICTED: DTW domain-containing protein 2 [Pan troglodytes].
402899373       LOC101017100                    299     eukaryota>metazoa>chordata>vertebrata                   Papio anubis                                                                    PREDICTED: LOW QUALITY PROTEIN: DTW domain-containing protein 2-like [Papio anubis].
297675823       LOC100447795                    298     eukaryota>metazoa>chordata>vertebrata                   Pongo abelii                                                                    PREDICTED: DTW domain-containing protein 2-like [Pongo abelii].
431907977       PAL_GLEAN10025060               293     eukaryota>metazoa>chordata>vertebrata                   Pteropus alecto                                                                 DTW domain-containing protein 2 [Pteropus alecto].
157822895       Dtwd2                           298     eukaryota>metazoa>chordata>vertebrata                   Rattus norvegicus                                                               DTW domain-containing protein 2 [Rattus norvegicus].
149064222       rCG_46778                       202     eukaryota>metazoa>chordata>vertebrata                   Rattus norvegicus                                                               rCG46778, isoform CRA_b [Rattus norvegicus].
403256070       DTWD2                           298     eukaryota>metazoa>chordata>vertebrata                   Saimiri boliviensis boliviensis                                                 PREDICTED: DTW domain-containing protein 2 [Saimiri boliviensis boliviensis].
395514220       LOC100922280                    275     eukaryota>metazoa>chordata>vertebrata                   Sarcophilus harrisii                                                            PREDICTED: DTW domain-containing protein 2-like [Sarcophilus harrisii].
395517026       LOC100923580                    180     eukaryota>metazoa>chordata>vertebrata                   Sarcophilus harrisii                                                            PREDICTED: DTW domain-containing protein 2-like [Sarcophilus harrisii].
350581022       LOC100523663                    199     eukaryota>metazoa>chordata>vertebrata                   Sus scrofa                                                                      PREDICTED: DTW domain-containing protein 2-like isoform 1, partial [Sus scrofa].
350581018       LOC100736959                    118     eukaryota>metazoa>chordata>vertebrata                   Sus scrofa                                                                      PREDICTED: DTW domain-containing protein 2-like [Sus scrofa].
350581020       LOC100737009                    204     eukaryota>metazoa>chordata>vertebrata                   Sus scrofa                                                                      PREDICTED: DTW domain-containing protein 2-like [Sus scrofa].
449514963       DTWD2                           365     eukaryota>metazoa>chordata>vertebrata                   Taeniopygia guttata                                                             PREDICTED: DTW domain-containing protein 2 [Taeniopygia guttata].
471378528       DTWD2                           276     eukaryota>metazoa>chordata>vertebrata                   Trichechus manatus latirostris                                                  PREDICTED: DTW domain-containing protein 2 [Trichechus manatus latirostris].
470647315       LOC101316905                    126     eukaryota>metazoa>chordata>vertebrata                   Tursiops truncatus                                                              PREDICTED: DTW domain-containing protein 2-like, partial [Tursiops truncatus].
62860026        dtwd2                           276     eukaryota>metazoa>chordata>vertebrata                   Xenopus (Silurana) tropicalis                                                   DTW domain containing 2 [Xenopus (Silurana) tropicalis].
148225582       dtwd2                           276     eukaryota>metazoa>chordata>vertebrata                   Xenopus laevis                                                                  DTW domain containing 2 [Xenopus laevis].
260785553       BRAFLDRAFT_94079                273     eukaryota>metazoa>chordata                              Branchiostoma floridae                                                          hypothetical protein BRAFLDRAFT_94079 [Branchiostoma floridae].
313242051       GSOID_T00024244001              223     eukaryota>metazoa>chordata                              Oikopleura dioica                                                               unnamed protein product [Oikopleura dioica].
198415464       LOC100184275                    227     eukaryota>metazoa>chordata                              Ciona intestinalis                                                              PREDICTED: DTW domain-containing protein 2-like [Ciona intestinalis].
313231156       GSOID_T00004577001              232     eukaryota>metazoa>chordata                              Oikopleura dioica                                                               unnamed protein product [Oikopleura dioica].
443691675       CAPTEDRAFT_101147               202     eukaryota>metazoa>annelida                              Capitella teleta                                                                hypothetical protein CAPTEDRAFT_101147 [Capitella teleta].
443692211       CAPTEDRAFT_180007               270     eukaryota>metazoa>annelida                              Capitella teleta                                                                hypothetical protein CAPTEDRAFT_180007 [Capitella teleta].
340372659       LOC100637747                    259     eukaryota>metazoa                                       Amphimedon queenslandica                                                        PREDICTED: DTW domain-containing protein 2-like [Amphimedon queenslandica].
358339962       CLF_100971                      247     eukaryota>metazoa                                       Clonorchis sinensis                                                             DTW domain-containing protein 2 [Clonorchis sinensis].
241628395       IscW_ISCW008675                 160     eukaryota>metazoa                                       Ixodes scapularis                                                               hypothetical protein IscW_ISCW008675 [Ixodes scapularis].
391333458       LOC100902541                    256     eukaryota>metazoa                                       Metaseiulus occidentalis                                                        PREDICTED: DTW domain-containing protein 2-like [Metaseiulus occidentalis].
471204323       EIN_381580                      207     eukaryota>amoebozoa>entamoebidae                        Entamoeba invadens IP1                                                          hypothetical protein EIN_381580 [Entamoeba invadens IP1].
407044571       ENU1_014080                     208     eukaryota>amoebozoa>entamoebidae                        Entamoeba nuttalli P19                                                          hypothetical protein ENU1_014080 [Entamoeba nuttalli P19].
183232037       EHI_024520                      208     eukaryota>amoebozoa>entamoebidae                        Entamoeba histolytica HM-1:IMSS                                                 hypothetical protein [Entamoeba histolytica HM-1:IMSS].
167379259       EDI_342080                      208     eukaryota>amoebozoa>entamoebidae                        Entamoeba dispar SAW760                                                         hypothetical protein [Entamoeba dispar SAW760].
294892728       Pmar_PMAR028208                 236     eukaryota>alveolata                                     Perkinsus marinus ATCC 50983                                                    conserved hypothetical protein [Perkinsus marinus ATCC 50983].
294887395       Pmar_PMAR017318                 191     eukaryota>alveolata                                     Perkinsus marinus ATCC 50983                                                    conserved hypothetical protein [Perkinsus marinus ATCC 50983].
494602193       -                               277     bacteria>verrucomicrobia                                Opitutaceae bacterium TAV1                                                      hypothetical protein [Opitutaceae bacterium TAV1].
495380075       -                               200     bacteria>verrucomicrobia                                Verrucomicrobiae bacterium DG1235                                               hypothetical protein [Verrucomicrobiae bacterium DG1235].
182415548       -                               241     bacteria>verrucomicrobia                                Opitutus terrae PB90-1                                                          DTW domain-containing protein [Opitutus terrae PB90-1].
497197630       -                               254     bacteria>verrucomicrobia                                Opitutaceae bacterium TAV5                                                      hypothetical protein [Opitutaceae bacterium TAV5].
494601373       -                               226     bacteria>verrucomicrobia                                Opitutaceae bacterium TAV1                                                      hypothetical protein [Opitutaceae bacterium TAV1].
497194682       -                               226     bacteria>verrucomicrobia                                Opitutaceae bacterium TAV5                                                      hypothetical protein [Opitutaceae bacterium TAV5].
182411974       -                               222     bacteria>verrucomicrobia                                Opitutus terrae PB90-1                                                          DTW domain-containing protein [Opitutus terrae PB90-1].
488863652       -                               205     bacteria>spirochaetes                                   Leptonema illini                                                                DTW domain containing protein [Leptonema illini].
488790454       -                               225     bacteria>spirochaetes                                   Treponema saccharophilum                                                        hypothetical protein [Treponema saccharophilum].
496394258       -                               226     bacteria>spirochaetes                                   Treponema sp. JC4                                                               hypothetical protein [Treponema sp. JC4].
328947538       -                               235     bacteria>spirochaetes                                   Treponema succinifaciens DSM 2489                                               DTW domain containing protein [Treponema succinifaciens DSM 2489].
392402867       Turpa_1325                      216     bacteria>spirochaetes                                   Turneriella parva DSM 21527                                                     DTW domain containing protein [Turneriella parva DSM 21527].
332297088       -                               253     bacteria>spirochaetes                                   Treponema brennaborense DSM 12168                                               DTW domain containing protein [Treponema brennaborense DSM 12168].
498320093       -                               151     bacteria>proteobacteria>gammaproteobacteria             Aeromonas                                                                       DTW domain-containing protein [Aeromonas].
498319937       -                               254     bacteria>proteobacteria>gammaproteobacteria             Aeromonas aquariorum                                                            DTW protein [Aeromonas aquariorum].
498360709       -                               249     bacteria>proteobacteria>gammaproteobacteria             Aeromonas caviae                                                                hypothetical protein [Aeromonas caviae].
498360840       -                               223     bacteria>proteobacteria>gammaproteobacteria             Aeromonas caviae                                                                hypothetical protein [Aeromonas caviae].
491501003       -                               244     bacteria>proteobacteria>gammaproteobacteria             Aeromonas diversa                                                               hypothetical protein [Aeromonas diversa].
491497962       -                               162     bacteria>proteobacteria>gammaproteobacteria             Aeromonas diversa                                                               DTW domain-containing protein [Aeromonas diversa].
491442154       -                               216     bacteria>proteobacteria>gammaproteobacteria             Aeromonas hydrophila                                                            hypothetical protein [Aeromonas hydrophila].
491442687       -                               253     bacteria>proteobacteria>gammaproteobacteria             Aeromonas hydrophila                                                            hypothetical protein [Aeromonas hydrophila].
117620945       -                               253     bacteria>proteobacteria>gammaproteobacteria             Aeromonas hydrophila subsp. hydrophila ATCC 7966                                DTW protein [Aeromonas hydrophila subsp. hydrophila ATCC 7966].
117619891       -                               228     bacteria>proteobacteria>gammaproteobacteria             Aeromonas hydrophila subsp. hydrophila ATCC 7966                                DTW domain-containing protein [Aeromonas hydrophila subsp. hydrophila ATCC 7966].
491470069       -                               226     bacteria>proteobacteria>gammaproteobacteria             Aeromonas media                                                                 hypothetical protein [Aeromonas media].
491471569       -                               258     bacteria>proteobacteria>gammaproteobacteria             Aeromonas media                                                                 hypothetical protein [Aeromonas media].
492636776       -                               169     bacteria>proteobacteria>gammaproteobacteria             Aeromonas molluscorum                                                           DTW domain-containing protein [Aeromonas molluscorum].
492636803       -                               280     bacteria>proteobacteria>gammaproteobacteria             Aeromonas molluscorum                                                           hypothetical protein [Aeromonas molluscorum].
145299217       -                               254     bacteria>proteobacteria>gammaproteobacteria             Aeromonas salmonicida subsp. salmonicida A449                                   hypothetical protein ASA_2259 [Aeromonas salmonicida subsp. salmonicida A449].
145298760       -                               206     bacteria>proteobacteria>gammaproteobacteria             Aeromonas salmonicida subsp. salmonicida A449                                   hypothetical protein ASA_1774 [Aeromonas salmonicida subsp. salmonicida A449].
491502211       -                               207     bacteria>proteobacteria>gammaproteobacteria             Aeromonas veronii                                                               hypothetical protein [Aeromonas veronii].
491475733       -                               251     bacteria>proteobacteria>gammaproteobacteria             Aeromonas veronii                                                               hypothetical protein [Aeromonas veronii].
491488732       -                               207     bacteria>proteobacteria>gammaproteobacteria             Aeromonas veronii                                                               hypothetical protein [Aeromonas veronii].
491479388       -                               207     bacteria>proteobacteria>gammaproteobacteria             Aeromonas veronii                                                               hypothetical protein [Aeromonas veronii].
491488315       -                               251     bacteria>proteobacteria>gammaproteobacteria             Aeromonas veronii                                                               hypothetical protein [Aeromonas veronii].
491503358       -                               251     bacteria>proteobacteria>gammaproteobacteria             Aeromonas veronii                                                               hypothetical protein [Aeromonas veronii].
330830004       -                               233     bacteria>proteobacteria>gammaproteobacteria             Aeromonas veronii B565                                                          DTW domain-containing protein [Aeromonas veronii B565].
330829404       -                               251     bacteria>proteobacteria>gammaproteobacteria             Aeromonas veronii B565                                                          hypothetical protein B565_1704 [Aeromonas veronii B565].
491559363       -                               195     bacteria>proteobacteria>gammaproteobacteria             Aliivibrio fischeri                                                             hypothetical protein [Aliivibrio fischeri].
491562418       -                               207     bacteria>proteobacteria>gammaproteobacteria             Aliivibrio fischeri                                                             hypothetical protein [Aliivibrio fischeri].
491561837       -                               247     bacteria>proteobacteria>gammaproteobacteria             Aliivibrio fischeri                                                             hypothetical protein [Aliivibrio fischeri].
209696350       -                               195     bacteria>proteobacteria>gammaproteobacteria             Aliivibrio salmonicida LFI1238                                                  hypothetical protein VSAL_I2954 [Aliivibrio salmonicida LFI1238].
209694917       -                               247     bacteria>proteobacteria>gammaproteobacteria             Aliivibrio salmonicida LFI1238                                                  hypothetical protein VSAL_I1391 [Aliivibrio salmonicida LFI1238].
495884088       -                               228     bacteria>proteobacteria>gammaproteobacteria             Alishewanella aestuarii                                                         hypothetical protein [Alishewanella aestuarii].
496273796       -                               234     bacteria>proteobacteria>gammaproteobacteria             Alishewanella agri                                                              hypothetical protein [Alishewanella agri].
496235809       -                               228     bacteria>proteobacteria>gammaproteobacteria             Alishewanella jeotgali                                                          hypothetical protein [Alishewanella jeotgali].
493845128       -                               211     bacteria>proteobacteria>gammaproteobacteria             Alteromonadales bacterium TW-7                                                  hypothetical protein [Alteromonadales bacterium TW-7].
493847546       -                               230     bacteria>proteobacteria>gammaproteobacteria             Alteromonadales bacterium TW-7                                                  hypothetical protein [Alteromonadales bacterium TW-7].
406598849       MASE_19715                      193     bacteria>proteobacteria>gammaproteobacteria             Alteromonas macleodii ATCC 27126                                                DTW domain containing protein [Alteromonas macleodii ATCC 27126].
406596937       MASE_09920                      230     bacteria>proteobacteria>gammaproteobacteria             Alteromonas macleodii ATCC 27126                                                hypothetical protein MASE_09920 [Alteromonas macleodii ATCC 27126].
410863675       amad1_20405                     194     bacteria>proteobacteria>gammaproteobacteria             Alteromonas macleodii AltDE1                                                    DTW domain containing protein [Alteromonas macleodii AltDE1].
407700166       AMBLS11_09605                   234     bacteria>proteobacteria>gammaproteobacteria             Alteromonas macleodii str. 'Black Sea 11'                                       hypothetical protein AMBLS11_09605 [Alteromonas macleodii str. 'Black Sea 11'].
407702010       AMBLS11_18905                   128     bacteria>proteobacteria>gammaproteobacteria             Alteromonas macleodii str. 'Black Sea 11'                                       DTW domain containing protein [Alteromonas macleodii str. 'Black Sea 11'].
332141502       MADE_1010520                    230     bacteria>proteobacteria>gammaproteobacteria             Alteromonas macleodii str. 'Deep ecotype'                                       hypothetical protein MADE_1010520 [Alteromonas macleodii str. 'Deep ecotype'].
332143468       MADE_1020430                    194     bacteria>proteobacteria>gammaproteobacteria             Alteromonas macleodii str. 'Deep ecotype'                                       hypothetical protein MADE_1020430 [Alteromonas macleodii str. 'Deep ecotype'].
407685773       AMEC673_19470                   193     bacteria>proteobacteria>gammaproteobacteria             Alteromonas macleodii str. 'English Channel 673'                                DTW domain containing protein [Alteromonas macleodii str. 'English Channel 673'].
407683929       AMEC673_10160                   230     bacteria>proteobacteria>gammaproteobacteria             Alteromonas macleodii str. 'English Channel 673'                                hypothetical protein AMEC673_10160 [Alteromonas macleodii str. 'English Channel 673'].
333893120       -                               235     bacteria>proteobacteria>gammaproteobacteria             Alteromonas sp. SN2                                                             hypothetical protein ambt_08325 [Alteromonas sp. SN2].
333895837       -                               193     bacteria>proteobacteria>gammaproteobacteria             Alteromonas sp. SN2                                                             DTW domain-containing protein [Alteromonas sp. SN2].
226945342       -                               213     bacteria>proteobacteria>gammaproteobacteria             Azotobacter vinelandii DJ                                                       DTW domain-containing protein [Azotobacter vinelandii DJ].
226945893       -                               232     bacteria>proteobacteria>gammaproteobacteria             Azotobacter vinelandii DJ                                                       hypothetical protein Avin_38500 [Azotobacter vinelandii DJ].
494074353       -                               177     bacteria>proteobacteria>gammaproteobacteria             Bermanella marisrubri                                                           hypothetical protein [Bermanella marisrubri].
494074986       -                               122     bacteria>proteobacteria>gammaproteobacteria             Bermanella marisrubri                                                           hypothetical protein [Bermanella marisrubri].
494075319       -                               223     bacteria>proteobacteria>gammaproteobacteria             Bermanella marisrubri                                                           hypothetical protein [Bermanella marisrubri].
496402647       -                               238     bacteria>proteobacteria>gammaproteobacteria             Brenneria sp. EniD312                                                           hypothetical protein [Brenneria sp. EniD312].
192360557       -                               278     bacteria>proteobacteria>gammaproteobacteria             Cellvibrio japonicus Ueda107                                                    DTW domain-containing protein [Cellvibrio japonicus Ueda107].
192359589       -                               206     bacteria>proteobacteria>gammaproteobacteria             Cellvibrio japonicus Ueda107                                                    DTW domain-containing protein [Cellvibrio japonicus Ueda107].
494914588       -                               208     bacteria>proteobacteria>gammaproteobacteria             Cellvibrio sp. BR                                                               hypothetical protein [Cellvibrio sp. BR].
494917946       -                               246     bacteria>proteobacteria>gammaproteobacteria             Cellvibrio sp. BR                                                               hypothetical protein [Cellvibrio sp. BR].
494919870       -                               169     bacteria>proteobacteria>gammaproteobacteria             Cellvibrio sp. BR                                                               DTW domain-containing protein [Cellvibrio sp. BR].
92115007        -                               247     bacteria>proteobacteria>gammaproteobacteria             Chromohalobacter salexigens DSM 3043                                            hypothetical protein Csal_2890 [Chromohalobacter salexigens DSM 3043].
489127766       -                               232     bacteria>proteobacteria>gammaproteobacteria             Citrobacter                                                                     hypothetical protein [Citrobacter].
489932029       -                               232     bacteria>proteobacteria>gammaproteobacteria             Citrobacter                                                                     hypothetical protein [Citrobacter].
489943744       -                               232     bacteria>proteobacteria>gammaproteobacteria             Citrobacter freundii                                                            DTW domain-containing protein [Citrobacter freundii].
157144484       -                               174     bacteria>proteobacteria>gammaproteobacteria             Citrobacter koseri ATCC BAA-895                                                 hypothetical protein CKO_00201 [Citrobacter koseri ATCC BAA-895].
283786198       -                               232     bacteria>proteobacteria>gammaproteobacteria             Citrobacter rodentium ICC168                                                    hypothetical protein ROD_25281 [Citrobacter rodentium ICC168].
500564804       WEU_03175                       232     bacteria>proteobacteria>gammaproteobacteria             Citrobacter sp. KTE32                                                           DTW domain-containing protein yfiP [Citrobacter sp. KTE32].
493734170       -                               174     bacteria>proteobacteria>gammaproteobacteria             Citrobacter youngae                                                             hypothetical protein [Citrobacter youngae].
71279615        CPS_2423                        179     bacteria>proteobacteria>gammaproteobacteria             Colwellia psychrerythraea 34H                                                   hypothetical protein CPS_2423 [Colwellia psychrerythraea 34H].
71282117        CPS_2149                        243     bacteria>proteobacteria>gammaproteobacteria             Colwellia psychrerythraea 34H                                                   DTW domain-containing protein [Colwellia psychrerythraea 34H].
71278044        CPS_1543                        266     bacteria>proteobacteria>gammaproteobacteria             Colwellia psychrerythraea 34H                                                   DTW domain-containing protein [Colwellia psychrerythraea 34H].
494938228       -                               233     bacteria>proteobacteria>gammaproteobacteria             Cronobacter condimenti                                                          FIG00554329: hypothetical protein [Cronobacter condimenti].
495025790       -                               240     bacteria>proteobacteria>gammaproteobacteria             Cronobacter dublinensis                                                         hypothetical protein [Cronobacter dublinensis].
495002338       -                               240     bacteria>proteobacteria>gammaproteobacteria             Cronobacter dublinensis                                                         FIG00554329: hypothetical protein [Cronobacter dublinensis].
495052559       -                               175     bacteria>proteobacteria>gammaproteobacteria             Cronobacter malonaticus                                                         FIG00554329: hypothetical protein [Cronobacter malonaticus].
495070875       -                               233     bacteria>proteobacteria>gammaproteobacteria             Cronobacter malonaticus                                                         FIG00554329: hypothetical protein [Cronobacter malonaticus].
495145305       -                               233     bacteria>proteobacteria>gammaproteobacteria             Cronobacter sakazakii                                                           FIG00554329: hypothetical protein [Cronobacter sakazakii].
495172407       -                               244     bacteria>proteobacteria>gammaproteobacteria             Cronobacter sakazakii                                                           FIG00554329: hypothetical protein [Cronobacter sakazakii].
389839951       -                               233     bacteria>proteobacteria>gammaproteobacteria             Cronobacter sakazakii ES15                                                      DTW domain-containing protein [Cronobacter sakazakii ES15].
449307200       -                               233     bacteria>proteobacteria>gammaproteobacteria             Cronobacter sakazakii SP291                                                     DTW domain-containing protein [Cronobacter sakazakii SP291].
495037811       -                               220     bacteria>proteobacteria>gammaproteobacteria             Cronobacter turicensis                                                          FIG00554329: hypothetical protein [Cronobacter turicensis].
260598964       -                               255     bacteria>proteobacteria>gammaproteobacteria             Cronobacter turicensis z3032                                                    DTW domain-containing protein yfiP [Cronobacter turicensis z3032].
494972276       -                               176     bacteria>proteobacteria>gammaproteobacteria             Cronobacter universalis                                                         FIG00554329: hypothetical protein [Cronobacter universalis].
307132326       -                               238     bacteria>proteobacteria>gammaproteobacteria             Dickeya dadantii 3937                                                           hypothetical protein Dda3937_00889 [Dickeya dadantii 3937].
271501804       -                               238     bacteria>proteobacteria>gammaproteobacteria             Dickeya dadantii Ech586                                                         DTW domain containing protein [Dickeya dadantii Ech586].
242238335       -                               237     bacteria>proteobacteria>gammaproteobacteria             Dickeya dadantii Ech703                                                         DTW domain containing protein [Dickeya dadantii Ech703].
251788490       -                               238     bacteria>proteobacteria>gammaproteobacteria             Dickeya zeae Ech1591                                                            DTW domain-containing protein [Dickeya zeae Ech1591].
238918593       -                               226     bacteria>proteobacteria>gammaproteobacteria             Edwardsiella ictaluri 93-146                                                    DTW domain protein [Edwardsiella ictaluri 93-146].
491436996       -                               230     bacteria>proteobacteria>gammaproteobacteria             Edwardsiella tarda                                                              hypothetical protein [Edwardsiella tarda].
269137916       -                               242     bacteria>proteobacteria>gammaproteobacteria             Edwardsiella tarda EIB202                                                       hypothetical protein ETAE_0558 [Edwardsiella tarda EIB202].
495777615       -                               232     bacteria>proteobacteria>gammaproteobacteria             Enterobacter                                                                    hypothetical protein [Enterobacter].
444355317       ST548_p3137                     206     bacteria>proteobacteria>gammaproteobacteria             Enterobacter aerogenes EA1509E                                                  FIG00731705: hypothetical protein [Enterobacter aerogenes EA1509E].
336246705       -                               232     bacteria>proteobacteria>gammaproteobacteria             Enterobacter aerogenes KCTC 2190                                                DTW domain-containing protein yfiP [Enterobacter aerogenes KCTC 2190].
345300446       -                               232     bacteria>proteobacteria>gammaproteobacteria             Enterobacter asburiae LF7a                                                      DTW domain-containing protein [Enterobacter asburiae LF7a].
493176095       -                               232     bacteria>proteobacteria>gammaproteobacteria             Enterobacter cancerogenus                                                       hypothetical protein [Enterobacter cancerogenus].
365971683       -                               232     bacteria>proteobacteria>gammaproteobacteria             Enterobacter cloacae EcWSU1                                                     DTW domain-containing protein YfiP [Enterobacter cloacae EcWSU1].
489957427       -                               232     bacteria>proteobacteria>gammaproteobacteria             Enterobacter cloacae complex                                                    hypothetical protein [Enterobacter cloacae complex].
296104256       ECL_03919                       232     bacteria>proteobacteria>gammaproteobacteria             Enterobacter cloacae subsp. cloacae ATCC 13047                                  DTW domain protein [Enterobacter cloacae subsp. cloacae ATCC 13047].
401764802       -                               232     bacteria>proteobacteria>gammaproteobacteria             Enterobacter cloacae subsp. cloacae ENHKU01                                     DTW domain-containing protein [Enterobacter cloacae subsp. cloacae ENHKU01].
479273784       -                               232     bacteria>proteobacteria>gammaproteobacteria             Enterobacter cloacae subsp. cloacae NCTC 9394                                   Uncharacterized conserved protein [Enterobacter cloacae subsp. cloacae NCTC 9394].
392980268       -                               232     bacteria>proteobacteria>gammaproteobacteria             Enterobacter cloacae subsp. dissolvens SDM                                      DTW domain-containing protein [Enterobacter cloacae subsp. dissolvens SDM].
493864911       -                               232     bacteria>proteobacteria>gammaproteobacteria             Enterobacter hormaechei                                                         hypothetical protein [Enterobacter hormaechei].
311278483       -                               249     bacteria>proteobacteria>gammaproteobacteria             Enterobacter lignolyticus SCF1                                                  DTW domain containing protein [Enterobacter lignolyticus SCF1].
498119940       -                               232     bacteria>proteobacteria>gammaproteobacteria             Enterobacter mori                                                               hypothetical protein [Enterobacter mori].
146312709       -                               232     bacteria>proteobacteria>gammaproteobacteria             Enterobacter sp. 638                                                            DTW domain-containing protein [Enterobacter sp. 638].
495734954       -                               233     bacteria>proteobacteria>gammaproteobacteria             Enterobacter sp. Ag1                                                            hypothetical protein [Enterobacter sp. Ag1].
496090180       -                               230     bacteria>proteobacteria>gammaproteobacteria             Enterobacteriaceae bacterium 9_2_54FAA                                          hypothetical protein [Enterobacteriaceae bacterium 9_2_54FAA].
440286537       -                               232     bacteria>proteobacteria>gammaproteobacteria             Enterobacteriaceae bacterium strain FGI 57                                      hypothetical protein D782_1093 [Enterobacteriaceae bacterium strain FGI 57].
292489190       -                               249     bacteria>proteobacteria>gammaproteobacteria             Erwinia amylovora CFBP1430                                                      hypothetical protein EAMY_2722 [Erwinia amylovora CFBP1430].
300718028       EbC_34530                       235     bacteria>proteobacteria>gammaproteobacteria             Erwinia billingiae Eb661                                                        DTW domain protein [Erwinia billingiae Eb661].
259907578       -                               236     bacteria>proteobacteria>gammaproteobacteria             Erwinia pyrifoliae Ep1/96                                                       hypothetical protein EpC_09050 [Erwinia pyrifoliae Ep1/96].
385785642       -                               236     bacteria>proteobacteria>gammaproteobacteria             Erwinia sp. Ejp617                                                              hypothetical protein EJP617_01830 [Erwinia sp. Ejp617].
188533073       -                               236     bacteria>proteobacteria>gammaproteobacteria             Erwinia tasmaniensis Et1/99                                                     hypothetical protein ETA_09260 [Erwinia tasmaniensis Et1/99].
446060333       -                               232     bacteria>proteobacteria>gammaproteobacteria             Escherichia                                                                     DTW domain-containing protein yfiP [Escherichia].
446060332       -                               232     bacteria>proteobacteria>gammaproteobacteria             Escherichia                                                                     DTW domain-containing protein yfiP [Escherichia].
446258535       -                               249     bacteria>proteobacteria>gammaproteobacteria             Escherichia albertii                                                            DTW domain-containing protein yfiP [Escherichia albertii].
446258534       -                               249     bacteria>proteobacteria>gammaproteobacteria             Escherichia albertii                                                            DTW domain-containing protein yfiP [Escherichia albertii].
446258531       -                               249     bacteria>proteobacteria>gammaproteobacteria             Escherichia coli                                                                DTW domain-containing protein yfiP [Escherichia coli].
446258532       -                               249     bacteria>proteobacteria>gammaproteobacteria             Escherichia coli                                                                DTW domain-containing protein yfiP [Escherichia coli].
446258533       -                               249     bacteria>proteobacteria>gammaproteobacteria             Escherichia coli                                                                DTW domain-containing protein yfiP [Escherichia coli].
446258530       -                               249     bacteria>proteobacteria>gammaproteobacteria             Escherichia coli                                                                DTW domain-containing protein yfiP [Escherichia coli].
446258528       -                               249     bacteria>proteobacteria>gammaproteobacteria             Escherichia coli                                                                DTW domain-containing protein yfiP [Escherichia coli].
486392771       -                               232     bacteria>proteobacteria>gammaproteobacteria             Escherichia coli                                                                DTW domain-containing protein yfiP [Escherichia coli].
446258537       -                               249     bacteria>proteobacteria>gammaproteobacteria             Escherichia coli                                                                DTW domain-containing protein yfiP [Escherichia coli].
446258538       -                               249     bacteria>proteobacteria>gammaproteobacteria             Escherichia coli                                                                DTW domain-containing protein yfiP [Escherichia coli].
446258527       -                               249     bacteria>proteobacteria>gammaproteobacteria             Escherichia coli                                                                DTW domain-containing protein yfiP [Escherichia coli].
486356881       -                               232     bacteria>proteobacteria>gammaproteobacteria             Escherichia coli                                                                DTW domain-containing protein yfiP [Escherichia coli].
486279655       -                               232     bacteria>proteobacteria>gammaproteobacteria             Escherichia coli                                                                DTW domain-containing protein yfiP [Escherichia coli].
486272318       -                               232     bacteria>proteobacteria>gammaproteobacteria             Escherichia coli                                                                DTW domain protein [Escherichia coli].
486141399       -                               232     bacteria>proteobacteria>gammaproteobacteria             Escherichia coli                                                                DTW domain-containing protein yfiP [Escherichia coli].
486117284       -                               232     bacteria>proteobacteria>gammaproteobacteria             Escherichia coli                                                                DTW domain protein [Escherichia coli].
485827924       -                               138     bacteria>proteobacteria>gammaproteobacteria             Escherichia coli                                                                hypothetical protein [Escherichia coli].
485862205       -                               232     bacteria>proteobacteria>gammaproteobacteria             Escherichia coli                                                                DTW domain protein [Escherichia coli].
485782953       -                               232     bacteria>proteobacteria>gammaproteobacteria             Escherichia coli                                                                DTW domain protein [Escherichia coli].
446258526       -                               249     bacteria>proteobacteria>gammaproteobacteria             Escherichia coli                                                                DTW domain-containing protein yfiP [Escherichia coli].
446258525       -                               249     bacteria>proteobacteria>gammaproteobacteria             Escherichia coli                                                                DTW domain-containing protein yfiP [Escherichia coli].
446258524       -                               249     bacteria>proteobacteria>gammaproteobacteria             Escherichia coli                                                                DTW domain-containing protein yfiP [Escherichia coli].
446258523       -                               249     bacteria>proteobacteria>gammaproteobacteria             Escherichia coli                                                                hypothetical protein [Escherichia coli].
446258522       -                               249     bacteria>proteobacteria>gammaproteobacteria             Escherichia coli                                                                DTW domain-containing protein yfiP [Escherichia coli].
446258521       -                               249     bacteria>proteobacteria>gammaproteobacteria             Escherichia coli                                                                DTW domain-containing protein yfiP [Escherichia coli].
446258520       -                               249     bacteria>proteobacteria>gammaproteobacteria             Escherichia coli                                                                DTW domain-containing protein yfiP [Escherichia coli].
446258519       -                               249     bacteria>proteobacteria>gammaproteobacteria             Escherichia coli                                                                DTW domain-containing protein yfiP [Escherichia coli].
446258518       -                               249     bacteria>proteobacteria>gammaproteobacteria             Escherichia coli                                                                DTW domain-containing protein yfiP [Escherichia coli].
446258517       -                               249     bacteria>proteobacteria>gammaproteobacteria             Escherichia coli                                                                DTW domain-containing protein yfiP [Escherichia coli].
446258516       -                               249     bacteria>proteobacteria>gammaproteobacteria             Escherichia coli                                                                DTW domain-containing protein yfiP [Escherichia coli].
446258515       -                               249     bacteria>proteobacteria>gammaproteobacteria             Escherichia coli                                                                DTW domain-containing protein yfiP [Escherichia coli].
446258513       -                               249     bacteria>proteobacteria>gammaproteobacteria             Escherichia coli                                                                DTW domain-containing protein yfiP [Escherichia coli].
446258512       -                               249     bacteria>proteobacteria>gammaproteobacteria             Escherichia coli                                                                DTW domain-containing protein yfiP [Escherichia coli].
446258510       -                               249     bacteria>proteobacteria>gammaproteobacteria             Escherichia coli                                                                DTW domain-containing protein yfiP [Escherichia coli].
487383562       -                               232     bacteria>proteobacteria>gammaproteobacteria             Escherichia coli                                                                DTW domain protein [Escherichia coli].
446258508       -                               249     bacteria>proteobacteria>gammaproteobacteria             Escherichia coli                                                                DTW domain-containing protein yfiP [Escherichia coli].
446258507       -                               249     bacteria>proteobacteria>gammaproteobacteria             Escherichia coli                                                                DTW domain-containing protein yfiP [Escherichia coli].
446258506       -                               249     bacteria>proteobacteria>gammaproteobacteria             Escherichia coli                                                                DTW domain-containing protein yfiP [Escherichia coli].
446258505       -                               249     bacteria>proteobacteria>gammaproteobacteria             Escherichia coli                                                                DTW domain-containing protein yfiP [Escherichia coli].
446258504       -                               249     bacteria>proteobacteria>gammaproteobacteria             Escherichia coli                                                                DTW domain-containing protein yfiP [Escherichia coli].
446258503       -                               249     bacteria>proteobacteria>gammaproteobacteria             Escherichia coli                                                                DTW domain-containing protein yfiP [Escherichia coli].
446258502       -                               249     bacteria>proteobacteria>gammaproteobacteria             Escherichia coli                                                                DTW domain-containing protein yfiP [Escherichia coli].
485769991       -                               155     bacteria>proteobacteria>gammaproteobacteria             Escherichia coli                                                                hypothetical protein [Escherichia coli].
446258499       -                               249     bacteria>proteobacteria>gammaproteobacteria             Escherichia coli                                                                DTW domain-containing protein yfiP [Escherichia coli].
446258497       -                               249     bacteria>proteobacteria>gammaproteobacteria             Escherichia coli                                                                DTW domain-containing protein yfiP [Escherichia coli].
446258496       -                               249     bacteria>proteobacteria>gammaproteobacteria             Escherichia coli                                                                DTW domain-containing protein yfiP [Escherichia coli].
487415785       -                               232     bacteria>proteobacteria>gammaproteobacteria             Escherichia coli                                                                DTW domain-containing protein yfiP [Escherichia coli].
446258494       -                               249     bacteria>proteobacteria>gammaproteobacteria             Escherichia coli                                                                DTW domain-containing protein yfiP [Escherichia coli].
446258426       -                               249     bacteria>proteobacteria>gammaproteobacteria             Escherichia coli                                                                DTW domain-containing protein yfiP [Escherichia coli].
487567464       -                               232     bacteria>proteobacteria>gammaproteobacteria             Escherichia coli                                                                DTW domain protein [Escherichia coli].
487601238       -                               232     bacteria>proteobacteria>gammaproteobacteria             Escherichia coli                                                                DTW domain protein [Escherichia coli].
487672317       -                               232     bacteria>proteobacteria>gammaproteobacteria             Escherichia coli                                                                DTW domain protein [Escherichia coli].
446258498       -                               249     bacteria>proteobacteria>gammaproteobacteria             Escherichia coli                                                                DTW domain-containing protein yfiP [Escherichia coli].
387608235       -                               249     bacteria>proteobacteria>gammaproteobacteria             Escherichia coli 042                                                            hypothetical protein EC042_2789 [Escherichia coli 042].
26248949        -                               249     bacteria>proteobacteria>gammaproteobacteria             Escherichia coli CFT073                                                         hypothetical protein c3108 [Escherichia coli CFT073].
446258501       -                               249     bacteria>proteobacteria>gammaproteobacteria             Escherichia fergusonii                                                          DTW domain-containing protein yfiP [Escherichia fergusonii].
446258500       -                               249     bacteria>proteobacteria>gammaproteobacteria             Escherichia fergusonii                                                          DTW domain-containing protein yfiP [Escherichia fergusonii].
488368747       -                               233     bacteria>proteobacteria>gammaproteobacteria             Escherichia hermannii                                                           hypothetical protein [Escherichia hermannii].
446258529       -                               249     bacteria>proteobacteria>gammaproteobacteria             Escherichia sp. TW09308                                                         DTW domain-containing protein yfiP [Escherichia sp. TW09308].
446060330       -                               232     bacteria>proteobacteria>gammaproteobacteria             Escherichia sp. TW11588                                                         DTW domain-containing protein yfiP [Escherichia sp. TW11588].
446060331       -                               232     bacteria>proteobacteria>gammaproteobacteria             Escherichia sp. TW14182                                                         DTW domain-containing protein yfiP [Escherichia sp. TW14182].
308051229       -                               196     bacteria>proteobacteria>gammaproteobacteria             Ferrimonas balearica DSM 9799                                                   hypothetical protein Fbal_3524 [Ferrimonas balearica DSM 9799].
308047903       -                               238     bacteria>proteobacteria>gammaproteobacteria             Ferrimonas balearica DSM 9799                                                   hypothetical protein Fbal_0181 [Ferrimonas balearica DSM 9799].
495760931       -                               230     bacteria>proteobacteria>gammaproteobacteria             Gallaecimonas xiamenensis                                                       DTW protein [Gallaecimonas xiamenensis].
495758452       -                               164     bacteria>proteobacteria>gammaproteobacteria             Gallaecimonas xiamenensis                                                       DTW domain-containing protein [Gallaecimonas xiamenensis].
494896208       -                               228     bacteria>proteobacteria>gammaproteobacteria             Glaciecola arctica                                                              conserved hypothetical protein [Glaciecola arctica].
494889383       -                               208     bacteria>proteobacteria>gammaproteobacteria             Glaciecola arctica                                                              hypothetical protein [Glaciecola arctica].
495262367       -                               200     bacteria>proteobacteria>gammaproteobacteria             Glaciecola chathamensis                                                         DTW domain-containing protein [Glaciecola chathamensis].
496121529       -                               166     bacteria>proteobacteria>gammaproteobacteria             Glaciecola lipolytica                                                           DTW domain containing protein [Glaciecola lipolytica].
494052822       -                               218     bacteria>proteobacteria>gammaproteobacteria             Glaciecola mesophila                                                            DTW domain-containing protein [Glaciecola mesophila].
348031029       -                               229     bacteria>proteobacteria>gammaproteobacteria             Glaciecola nitratireducens FR1064                                               DTW domain containing protein [Glaciecola nitratireducens FR1064].
492863402       -                               197     bacteria>proteobacteria>gammaproteobacteria             Glaciecola pallidula                                                            hypothetical protein [Glaciecola pallidula].
494163248       -                               190     bacteria>proteobacteria>gammaproteobacteria             Glaciecola polaris                                                              DTW domain-containing protein [Glaciecola polaris].
494909313       -                               219     bacteria>proteobacteria>gammaproteobacteria             Glaciecola psychrophila                                                         conserved hypothetical protein [Glaciecola psychrophila].
476414253       -                               120     bacteria>proteobacteria>gammaproteobacteria             Glaciecola psychrophila 170                                                     hypothetical protein C427_2714 [Glaciecola psychrophila 170].
476417120       -                               204     bacteria>proteobacteria>gammaproteobacteria             Glaciecola psychrophila 170                                                     hypothetical protein C427_5596 [Glaciecola psychrophila 170].
492850932       -                               192     bacteria>proteobacteria>gammaproteobacteria             Glaciecola punicea                                                              hypothetical protein [Glaciecola punicea].
332308596       -                               200     bacteria>proteobacteria>gammaproteobacteria             Glaciecola sp. 4H-3-7+YE-5                                                      DTW domain containing protein [Glaciecola sp. 4H-3-7+YE-5].
497867439       -                               182     bacteria>proteobacteria>gammaproteobacteria             Glaciecola sp. HTCC2999                                                         DTW domain-containing protein [Glaciecola sp. HTCC2999].
491647951       -                               241     bacteria>proteobacteria>gammaproteobacteria             Grimontia hollisae                                                              hypothetical protein [Grimontia hollisae].
491649160       -                               206     bacteria>proteobacteria>gammaproteobacteria             Grimontia hollisae                                                              hypothetical protein [Grimontia hollisae].
491644190       -                               210     bacteria>proteobacteria>gammaproteobacteria             Grimontia hollisae                                                              hypothetical protein [Grimontia hollisae].
488499090       -                               242     bacteria>proteobacteria>gammaproteobacteria             Grimontia sp. AK16                                                              putative cytoplasmic protein [Grimontia sp. AK16].
488492106       -                               178     bacteria>proteobacteria>gammaproteobacteria             Grimontia sp. AK16                                                              hypothetical protein [Grimontia sp. AK16].
488492001       -                               204     bacteria>proteobacteria>gammaproteobacteria             Grimontia sp. AK16                                                              hypothetical protein [Grimontia sp. AK16].
490190618       -                               173     bacteria>proteobacteria>gammaproteobacteria             Hafnia alvei                                                                    hypothetical protein [Hafnia alvei].
307543957       -                               263     bacteria>proteobacteria>gammaproteobacteria             Halomonas elongata DSM 2581                                                     hypothetical protein HELO_1368 [Halomonas elongata DSM 2581].
496391843       -                               256     bacteria>proteobacteria>gammaproteobacteria             Halomonas sp. GFAJ-1                                                            hypothetical protein [Halomonas sp. GFAJ-1].
496246402       -                               267     bacteria>proteobacteria>gammaproteobacteria             Halomonas sp. HAL1                                                              hypothetical protein [Halomonas sp. HAL1].
498312456       -                               266     bacteria>proteobacteria>gammaproteobacteria             Halomonas sp. KM-1                                                              hypothetical protein [Halomonas sp. KM-1].
497410046       -                               253     bacteria>proteobacteria>gammaproteobacteria             Halomonas sp. TD01                                                              hypothetical protein [Halomonas sp. TD01].
495764783       -                               174     bacteria>proteobacteria>gammaproteobacteria             Idiomarina xiamenensis                                                          hypothetical protein [Idiomarina xiamenensis].
490278866       -                               232     bacteria>proteobacteria>gammaproteobacteria             Klebsiella                                                                      hypothetical protein [Klebsiella].
490234440       -                               232     bacteria>proteobacteria>gammaproteobacteria             Klebsiella oxytoca                                                              DTW domain-containing protein [Klebsiella oxytoca].
490216109       -                               232     bacteria>proteobacteria>gammaproteobacteria             Klebsiella oxytoca                                                              hypothetical protein [Klebsiella oxytoca].
491004764       -                               232     bacteria>proteobacteria>gammaproteobacteria             Klebsiella oxytoca                                                              hypothetical protein [Klebsiella oxytoca].
490206174       -                               232     bacteria>proteobacteria>gammaproteobacteria             Klebsiella oxytoca                                                              hypothetical protein [Klebsiella oxytoca].
490225370       -                               232     bacteria>proteobacteria>gammaproteobacteria             Klebsiella oxytoca                                                              hypothetical protein [Klebsiella oxytoca].
490991555       -                               232     bacteria>proteobacteria>gammaproteobacteria             Klebsiella oxytoca                                                              hypothetical protein [Klebsiella oxytoca].
397659416       -                               232     bacteria>proteobacteria>gammaproteobacteria             Klebsiella oxytoca E718                                                         hypothetical protein A225_4433 [Klebsiella oxytoca E718].
375262299       -                               232     bacteria>proteobacteria>gammaproteobacteria             Klebsiella oxytoca KCTC 1686                                                    DTW domain-containing protein yfiP [Klebsiella oxytoca KCTC 1686].
490306734       -                               232     bacteria>proteobacteria>gammaproteobacteria             Klebsiella pneumoniae                                                           DTW domain-containing protein [Klebsiella pneumoniae].
490289542       -                               232     bacteria>proteobacteria>gammaproteobacteria             Klebsiella pneumoniae                                                           hypothetical protein [Klebsiella pneumoniae].
490246185       -                               232     bacteria>proteobacteria>gammaproteobacteria             Klebsiella pneumoniae                                                           hypothetical protein [Klebsiella pneumoniae].
206577995       -                               232     bacteria>proteobacteria>gammaproteobacteria             Klebsiella pneumoniae 342                                                       DTW domain protein [Klebsiella pneumoniae 342].
386036060       -                               232     bacteria>proteobacteria>gammaproteobacteria             Klebsiella pneumoniae KCTC 2242                                                 DTW domain-containing protein yfiP [Klebsiella pneumoniae KCTC 2242].
152971437       -                               232     bacteria>proteobacteria>gammaproteobacteria             Klebsiella pneumoniae subsp. pneumoniae MGH 78578                               hypothetical protein KPN_02906 [Klebsiella pneumoniae subsp. pneumoniae MGH 78578].
496079249       -                               232     bacteria>proteobacteria>gammaproteobacteria             Klebsiella sp. 1_1_55                                                           hypothetical protein [Klebsiella sp. 1_1_55].
497340137       -                               232     bacteria>proteobacteria>gammaproteobacteria             Klebsiella sp. OBRC7                                                            hypothetical protein [Klebsiella sp. OBRC7].
494612516       -                               232     bacteria>proteobacteria>gammaproteobacteria             Kosakonia radicincitans                                                         hypothetical protein [Kosakonia radicincitans].
385333754       -                               168     bacteria>proteobacteria>gammaproteobacteria             Marinobacter adhaerens HP15                                                     DTW domain containing protein [Marinobacter adhaerens HP15].
120553258       -                               211     bacteria>proteobacteria>gammaproteobacteria             Marinobacter aquaeolei VT8                                                      DTW domain-containing protein [Marinobacter aquaeolei VT8].
387812712       -                               211     bacteria>proteobacteria>gammaproteobacteria             Marinobacter hydrocarbonoclasticus ATCC 49840                                   hypothetical protein MARHY0263 [Marinobacter hydrocarbonoclasticus ATCC 49840].
495452464       -                               135     bacteria>proteobacteria>gammaproteobacteria             Marinobacter manganoxydans                                                      hypothetical protein [Marinobacter manganoxydans].
490716611       -                               201     bacteria>proteobacteria>gammaproteobacteria             Marinobacter nanhaiticus                                                        hypothetical protein [Marinobacter nanhaiticus].
498010894       -                               219     bacteria>proteobacteria>gammaproteobacteria             Marinobacterium stanieri                                                        hypothetical protein [Marinobacterium stanieri].
326796217       -                               157     bacteria>proteobacteria>gammaproteobacteria             Marinomonas mediterranea MMB-1                                                  DTW domain containing protein [Marinomonas mediterranea MMB-1].
326794778       -                               198     bacteria>proteobacteria>gammaproteobacteria             Marinomonas mediterranea MMB-1                                                  DTW domain containing protein [Marinomonas mediterranea MMB-1].
326794915       -                               233     bacteria>proteobacteria>gammaproteobacteria             Marinomonas mediterranea MMB-1                                                  DTW domain containing protein [Marinomonas mediterranea MMB-1].
333907619       -                               194     bacteria>proteobacteria>gammaproteobacteria             Marinomonas posidonica IVIA-Po-181                                              DTW domain-containing protein [Marinomonas posidonica IVIA-Po-181].
333907890       -                               238     bacteria>proteobacteria>gammaproteobacteria             Marinomonas posidonica IVIA-Po-181                                              DTW domain-containing protein [Marinomonas posidonica IVIA-Po-181].
333907076       -                               152     bacteria>proteobacteria>gammaproteobacteria             Marinomonas posidonica IVIA-Po-181                                              DTW domain-containing protein [Marinomonas posidonica IVIA-Po-181].
497521907       -                               196     bacteria>proteobacteria>gammaproteobacteria             Marinomonas sp. MED121                                                          hypothetical protein [Marinomonas sp. MED121].
497521178       -                               165     bacteria>proteobacteria>gammaproteobacteria             Marinomonas sp. MED121                                                          hypothetical protein [Marinomonas sp. MED121].
497517606       -                               192     bacteria>proteobacteria>gammaproteobacteria             Marinomonas sp. MED121                                                          hypothetical protein [Marinomonas sp. MED121].
152996841       -                               200     bacteria>proteobacteria>gammaproteobacteria             Marinomonas sp. MWYL1                                                           DTW domain-containing protein [Marinomonas sp. MWYL1].
152996559       -                               236     bacteria>proteobacteria>gammaproteobacteria             Marinomonas sp. MWYL1                                                           DTW domain-containing protein [Marinomonas sp. MWYL1].
152997618       -                               152     bacteria>proteobacteria>gammaproteobacteria             Marinomonas sp. MWYL1                                                           hypothetical protein Mmwyl1_3616 [Marinomonas sp. MWYL1].
497816346       -                               178     bacteria>proteobacteria>gammaproteobacteria             Microbulbifer agarilyticus                                                      DTW domain-containing protein [Microbulbifer agarilyticus].
497819403       -                               237     bacteria>proteobacteria>gammaproteobacteria             Microbulbifer agarilyticus                                                      hypothetical protein [Microbulbifer agarilyticus].
497818175       -                               162     bacteria>proteobacteria>gammaproteobacteria             Microbulbifer agarilyticus                                                      DTW protein [Microbulbifer agarilyticus].
491042814       -                               234     bacteria>proteobacteria>gammaproteobacteria             Morganella morganii                                                             hypothetical protein [Morganella morganii].
455738469       -                               234     bacteria>proteobacteria>gammaproteobacteria             Morganella morganii subsp. morganii KT                                          hypothetical protein MU9_1316 [Morganella morganii subsp. morganii KT].
492900808       -                               153     bacteria>proteobacteria>gammaproteobacteria             Moritella sp. PE36                                                              hypothetical protein [Moritella sp. PE36].
492904188       -                               208     bacteria>proteobacteria>gammaproteobacteria             Moritella sp. PE36                                                              hypothetical protein [Moritella sp. PE36].
492904006       -                               239     bacteria>proteobacteria>gammaproteobacteria             Moritella sp. PE36                                                              hypothetical protein [Moritella sp. PE36].
494080939       -                               181     bacteria>proteobacteria>gammaproteobacteria             Neptuniibacter caesariensis                                                     DTW domain protein [Neptuniibacter caesariensis].
494078731       -                               196     bacteria>proteobacteria>gammaproteobacteria             Neptuniibacter caesariensis                                                     hypothetical cytosolic protein [Neptuniibacter caesariensis].
374334825       -                               186     bacteria>proteobacteria>gammaproteobacteria             Oceanimonas sp. GK1                                                             DTW domain-containing protein [Oceanimonas sp. GK1].
498357995       -                               225     bacteria>proteobacteria>gammaproteobacteria             Pantoea agglomerans                                                             hypothetical protein [Pantoea agglomerans].
489947082       -                               225     bacteria>proteobacteria>gammaproteobacteria             Pantoea agglomerans                                                             hypothetical protein [Pantoea agglomerans].
291618557       -                               226     bacteria>proteobacteria>gammaproteobacteria             Pantoea ananatis LMG 20103                                                      hypothetical protein PANA_3004 [Pantoea ananatis LMG 20103].
378766001       -                               226     bacteria>proteobacteria>gammaproteobacteria             Pantoea ananatis LMG 5342                                                       DTW domain protein [Pantoea ananatis LMG 5342].
386078257       -                               226     bacteria>proteobacteria>gammaproteobacteria             Pantoea ananatis PA13                                                           DTW domain-containing protein YfiP [Pantoea ananatis PA13].
317049242       -                               231     bacteria>proteobacteria>gammaproteobacteria             Pantoea sp. At-9b                                                               DTW domain-containing protein [Pantoea sp. At-9b].
495161587       -                               230     bacteria>proteobacteria>gammaproteobacteria             Pantoea sp. GM01                                                                hypothetical protein [Pantoea sp. GM01].
497943647       -                               225     bacteria>proteobacteria>gammaproteobacteria             Pantoea sp. SL1_M5                                                              hypothetical protein [Pantoea sp. SL1_M5].
496379549       -                               225     bacteria>proteobacteria>gammaproteobacteria             Pantoea sp. Sc1                                                                 hypothetical protein [Pantoea sp. Sc1].
495376939       -                               234     bacteria>proteobacteria>gammaproteobacteria             Pantoea sp. YR343                                                               hypothetical protein [Pantoea sp. YR343].
496208819       -                               225     bacteria>proteobacteria>gammaproteobacteria             Pantoea sp. aB                                                                  hypothetical protein [Pantoea sp. aB].
493071365       -                               226     bacteria>proteobacteria>gammaproteobacteria             Pantoea stewartii                                                               hypothetical protein [Pantoea stewartii].
308187858       -                               225     bacteria>proteobacteria>gammaproteobacteria             Pantoea vagans C9-1                                                             hypothetical protein Pvag_2367 [Pantoea vagans C9-1].
50122439        -                               250     bacteria>proteobacteria>gammaproteobacteria             Pectobacterium atrosepticum SCRI1043                                            hypothetical protein ECA3518 [Pectobacterium atrosepticum SCRI1043].
497968521       -                               237     bacteria>proteobacteria>gammaproteobacteria             Pectobacterium carotovorum                                                      hypothetical protein [Pectobacterium carotovorum].
497991232       -                               237     bacteria>proteobacteria>gammaproteobacteria             Pectobacterium carotovorum                                                      hypothetical protein [Pectobacterium carotovorum].
253689698       -                               249     bacteria>proteobacteria>gammaproteobacteria             Pectobacterium carotovorum subsp. carotovorum PC1                               DTW domain-containing protein [Pectobacterium carotovorum subsp. carotovorum PC1].
403059776       -                               237     bacteria>proteobacteria>gammaproteobacteria             Pectobacterium carotovorum subsp. carotovorum PCC21                             hypothetical protein PCC21_033370 [Pectobacterium carotovorum subsp. carotovorum PCC21].
492819040       -                               244     bacteria>proteobacteria>gammaproteobacteria             Pectobacterium wasabiae                                                         hypothetical protein [Pectobacterium wasabiae].
261822729       -                               244     bacteria>proteobacteria>gammaproteobacteria             Pectobacterium wasabiae WPP163                                                  DTW domain containing protein [Pectobacterium wasabiae WPP163].
491510411       -                               241     bacteria>proteobacteria>gammaproteobacteria             Photobacterium angustum                                                         hypothetical protein [Photobacterium angustum].
491511455       -                               203     bacteria>proteobacteria>gammaproteobacteria             Photobacterium angustum                                                         hypothetical protein [Photobacterium angustum].
491514461       -                               195     bacteria>proteobacteria>gammaproteobacteria             Photobacterium angustum                                                         hypothetical protein [Photobacterium angustum].
491444621       -                               193     bacteria>proteobacteria>gammaproteobacteria             Photobacterium damselae                                                         hypothetical protein [Photobacterium damselae].
491440482       -                               206     bacteria>proteobacteria>gammaproteobacteria             Photobacterium damselae                                                         hypothetical protein [Photobacterium damselae].
491441396       -                               240     bacteria>proteobacteria>gammaproteobacteria             Photobacterium damselae                                                         hypothetical protein [Photobacterium damselae].
358410576       PDP_0194                        156     bacteria>proteobacteria>gammaproteobacteria             Photobacterium damselae subsp. piscicida                                        hypothetical protein PDP_0194, partial [Photobacterium damselae subsp. piscicida].
496276203       -                               194     bacteria>proteobacteria>gammaproteobacteria             Photobacterium leiognathi                                                       hypothetical protein [Photobacterium leiognathi].
496275099       -                               239     bacteria>proteobacteria>gammaproteobacteria             Photobacterium leiognathi                                                       hypothetical protein [Photobacterium leiognathi].
496275617       -                               205     bacteria>proteobacteria>gammaproteobacteria             Photobacterium leiognathi                                                       hypothetical protein [Photobacterium leiognathi].
493275442       -                               193     bacteria>proteobacteria>gammaproteobacteria             Photobacterium profundum                                                        hypothetical protein [Photobacterium profundum].
493273951       -                               239     bacteria>proteobacteria>gammaproteobacteria             Photobacterium profundum                                                        hypothetical protein [Photobacterium profundum].
493275190       -                               216     bacteria>proteobacteria>gammaproteobacteria             Photobacterium profundum                                                        hypothetical protein [Photobacterium profundum].
54309790        -                               217     bacteria>proteobacteria>gammaproteobacteria             Photobacterium profundum SS9                                                    hypothetical protein PBPRA2629 [Photobacterium profundum SS9].
54308935        -                               242     bacteria>proteobacteria>gammaproteobacteria             Photobacterium profundum SS9                                                    hypothetical protein PBPRA1746 [Photobacterium profundum SS9].
54307354        -                               193     bacteria>proteobacteria>gammaproteobacteria             Photobacterium profundum SS9                                                    hypothetical protein PBPRA0133 [Photobacterium profundum SS9].
494734534       -                               238     bacteria>proteobacteria>gammaproteobacteria             Photobacterium sp. AK15                                                         Putative cytoplasmic protein [Photobacterium sp. AK15].
494733771       -                               192     bacteria>proteobacteria>gammaproteobacteria             Photobacterium sp. AK15                                                         hypothetical protein [Photobacterium sp. AK15].
494734275       -                               203     bacteria>proteobacteria>gammaproteobacteria             Photobacterium sp. AK15                                                         hypothetical protein [Photobacterium sp. AK15].
493697632       -                               195     bacteria>proteobacteria>gammaproteobacteria             Photobacterium sp. SKA34                                                        hypothetical protein [Photobacterium sp. SKA34].
493695712       -                               241     bacteria>proteobacteria>gammaproteobacteria             Photobacterium sp. SKA34                                                        hypothetical protein [Photobacterium sp. SKA34].
493696566       -                               203     bacteria>proteobacteria>gammaproteobacteria             Photobacterium sp. SKA34                                                        hypothetical protein [Photobacterium sp. SKA34].
253990661       -                               224     bacteria>proteobacteria>gammaproteobacteria             Photorhabdus asymbiotica                                                        conserved hypothetical protein [Photorhabdus asymbiotica].
498302618       -                               228     bacteria>proteobacteria>gammaproteobacteria             Plautia stali symbiont                                                          hypothetical protein [Plautia stali symbiont].
499149072       -                               235     bacteria>proteobacteria>gammaproteobacteria             Plesiomonas shigelloides                                                        hypothetical protein [Plesiomonas shigelloides].
490366430       -                               170     bacteria>proteobacteria>gammaproteobacteria             Proteus mirabilis                                                               hypothetical protein [Proteus mirabilis].
197284299       -                               227     bacteria>proteobacteria>gammaproteobacteria             Proteus mirabilis HI4320                                                        hypothetical protein PMI0399 [Proteus mirabilis HI4320].
493584772       -                               178     bacteria>proteobacteria>gammaproteobacteria             Proteus penneri                                                                 hypothetical protein [Proteus penneri].
493712739       -                               233     bacteria>proteobacteria>gammaproteobacteria             Providencia alcalifaciens                                                       hypothetical protein [Providencia alcalifaciens].
496187660       -                               187     bacteria>proteobacteria>gammaproteobacteria             Providencia burhodogranariea                                                    hypothetical protein [Providencia burhodogranariea].
490377542       -                               175     bacteria>proteobacteria>gammaproteobacteria             Providencia rettgeri                                                            hypothetical protein [Providencia rettgeri].
493866186       -                               175     bacteria>proteobacteria>gammaproteobacteria             Providencia rustigianii                                                         hypothetical protein [Providencia rustigianii].
496191793       -                               245     bacteria>proteobacteria>gammaproteobacteria             Providencia sneebia                                                             hypothetical protein [Providencia sneebia].
491055932       -                               179     bacteria>proteobacteria>gammaproteobacteria             Providencia stuartii                                                            hypothetical protein [Providencia stuartii].
386744382       -                               237     bacteria>proteobacteria>gammaproteobacteria             Providencia stuartii MRSN 2154                                                  hypothetical protein S70_15230 [Providencia stuartii MRSN 2154].
495406411       -                               230     bacteria>proteobacteria>gammaproteobacteria             Pseudoalteromonas                                                               hypothetical protein [Pseudoalteromonas].
494620503       -                               228     bacteria>proteobacteria>gammaproteobacteria             Pseudoalteromonas                                                               hypothetical protein [Pseudoalteromonas].
495388895       -                               197     bacteria>proteobacteria>gammaproteobacteria             Pseudoalteromonas                                                               hypothetical protein [Pseudoalteromonas].
490725382       -                               228     bacteria>proteobacteria>gammaproteobacteria             Pseudoalteromonas agarivorans                                                   hypothetical protein [Pseudoalteromonas agarivorans].
490725232       -                               198     bacteria>proteobacteria>gammaproteobacteria             Pseudoalteromonas agarivorans                                                   hypothetical protein [Pseudoalteromonas agarivorans].
498239188       -                               226     bacteria>proteobacteria>gammaproteobacteria             Pseudoalteromonas arctica                                                       hypothetical protein [Pseudoalteromonas arctica].
498240081       -                               198     bacteria>proteobacteria>gammaproteobacteria             Pseudoalteromonas arctica                                                       hypothetical protein [Pseudoalteromonas arctica].
109900590       -                               196     bacteria>proteobacteria>gammaproteobacteria             Pseudoalteromonas atlantica T6c                                                 DTW domain-containing protein [Pseudoalteromonas atlantica T6c].
498053629       -                               224     bacteria>proteobacteria>gammaproteobacteria             Pseudoalteromonas citrea                                                        hypothetical protein [Pseudoalteromonas citrea].
498050386       -                               198     bacteria>proteobacteria>gammaproteobacteria             Pseudoalteromonas citrea                                                        hypothetical protein [Pseudoalteromonas citrea].
498291914       -                               221     bacteria>proteobacteria>gammaproteobacteria             Pseudoalteromonas flavipulchra                                                  hypothetical protein [Pseudoalteromonas flavipulchra].
498290435       -                               196     bacteria>proteobacteria>gammaproteobacteria             Pseudoalteromonas flavipulchra                                                  hypothetical protein [Pseudoalteromonas flavipulchra].
489047540       -                               226     bacteria>proteobacteria>gammaproteobacteria             Pseudoalteromonas haloplanktis                                                  hypothetical protein [Pseudoalteromonas haloplanktis].
489049458       -                               198     bacteria>proteobacteria>gammaproteobacteria             Pseudoalteromonas haloplanktis                                                  hypothetical protein [Pseudoalteromonas haloplanktis].
77360264        -                               197     bacteria>proteobacteria>gammaproteobacteria             Pseudoalteromonas haloplanktis TAC125                                           hypothetical protein PSHAa1321 [Pseudoalteromonas haloplanktis TAC125].
77359360        -                               198     bacteria>proteobacteria>gammaproteobacteria             Pseudoalteromonas haloplanktis TAC125                                           hypothetical protein PSHAa0394 [Pseudoalteromonas haloplanktis TAC125].
491636832       -                               163     bacteria>proteobacteria>gammaproteobacteria             Pseudoalteromonas luteoviolacea                                                 hypothetical protein [Pseudoalteromonas luteoviolacea].
491634012       -                               225     bacteria>proteobacteria>gammaproteobacteria             Pseudoalteromonas luteoviolacea                                                 FIG00952298: hypothetical protein [Pseudoalteromonas luteoviolacea].
498243341       -                               197     bacteria>proteobacteria>gammaproteobacteria             Pseudoalteromonas marina                                                        hypothetical protein [Pseudoalteromonas marina].
498058151       -                               221     bacteria>proteobacteria>gammaproteobacteria             Pseudoalteromonas piscicida                                                     hypothetical protein [Pseudoalteromonas piscicida].
498057483       -                               163     bacteria>proteobacteria>gammaproteobacteria             Pseudoalteromonas piscicida                                                     hypothetical protein [Pseudoalteromonas piscicida].
498068837       -                               222     bacteria>proteobacteria>gammaproteobacteria             Pseudoalteromonas rubra                                                         hypothetical protein [Pseudoalteromonas rubra].
498067485       -                               171     bacteria>proteobacteria>gammaproteobacteria             Pseudoalteromonas rubra                                                         hypothetical protein [Pseudoalteromonas rubra].
495390244       -                               228     bacteria>proteobacteria>gammaproteobacteria             Pseudoalteromonas sp. BSi20311                                                  hypothetical protein [Pseudoalteromonas sp. BSi20311].
494856441       -                               198     bacteria>proteobacteria>gammaproteobacteria             Pseudoalteromonas sp. BSi20429                                                  hypothetical protein [Pseudoalteromonas sp. BSi20429].
494860017       -                               226     bacteria>proteobacteria>gammaproteobacteria             Pseudoalteromonas sp. BSi20429                                                  hypothetical protein [Pseudoalteromonas sp. BSi20429].
495741870       -                               228     bacteria>proteobacteria>gammaproteobacteria             Pseudoalteromonas sp. BSi20439                                                  hypothetical protein [Pseudoalteromonas sp. BSi20439].
495406784       -                               197     bacteria>proteobacteria>gammaproteobacteria             Pseudoalteromonas sp. BSi20480                                                  hypothetical protein [Pseudoalteromonas sp. BSi20480].
495413686       -                               198     bacteria>proteobacteria>gammaproteobacteria             Pseudoalteromonas sp. BSi20495                                                  hypothetical protein [Pseudoalteromonas sp. BSi20495].
495445982       -                               226     bacteria>proteobacteria>gammaproteobacteria             Pseudoalteromonas sp. BSi20652                                                  hypothetical protein [Pseudoalteromonas sp. BSi20652].
495443390       -                               198     bacteria>proteobacteria>gammaproteobacteria             Pseudoalteromonas sp. BSi20652                                                  hypothetical protein [Pseudoalteromonas sp. BSi20652].
494617172       -                               198     bacteria>proteobacteria>gammaproteobacteria             Pseudoalteromonas sp. Bsw20308                                                  hypothetical protein [Pseudoalteromonas sp. Bsw20308].
315127763       -                               198     bacteria>proteobacteria>gammaproteobacteria             Pseudoalteromonas sp. SM9913                                                    hypothetical protein PSM_A2701 [Pseudoalteromonas sp. SM9913].
315126744       -                               211     bacteria>proteobacteria>gammaproteobacteria             Pseudoalteromonas sp. SM9913                                                    hypothetical protein PSM_A1669 [Pseudoalteromonas sp. SM9913].
498247578       -                               223     bacteria>proteobacteria>gammaproteobacteria             Pseudoalteromonas spongiae                                                      hypothetical protein [Pseudoalteromonas spongiae].
498246143       -                               150     bacteria>proteobacteria>gammaproteobacteria             Pseudoalteromonas spongiae                                                      hypothetical protein [Pseudoalteromonas spongiae].
497525436       -                               167     bacteria>proteobacteria>gammaproteobacteria             Pseudoalteromonas tunicata                                                      hypothetical protein [Pseudoalteromonas tunicata].
497524128       -                               233     bacteria>proteobacteria>gammaproteobacteria             Pseudoalteromonas tunicata                                                      hypothetical protein [Pseudoalteromonas tunicata].
498077504       -                               228     bacteria>proteobacteria>gammaproteobacteria             Pseudoalteromonas undina                                                        hypothetical protein [Pseudoalteromonas undina].
498076028       -                               197     bacteria>proteobacteria>gammaproteobacteria             Pseudoalteromonas undina                                                        hypothetical protein [Pseudoalteromonas undina].
495189722       -                               198     bacteria>proteobacteria>gammaproteobacteria             Pseudomonas                                                                     hypothetical protein [Pseudomonas].
497898527       -                               239     bacteria>proteobacteria>gammaproteobacteria             Pseudomonas                                                                     hypothetical protein [Pseudomonas].
489301170       -                               236     bacteria>proteobacteria>gammaproteobacteria             Pseudomonas                                                                     hypothetical protein [Pseudomonas].
495183946       -                               249     bacteria>proteobacteria>gammaproteobacteria             Pseudomonas                                                                     hypothetical protein [Pseudomonas].
489303284       -                               198     bacteria>proteobacteria>gammaproteobacteria             Pseudomonas                                                                     hypothetical protein [Pseudomonas].
489255319       -                               238     bacteria>proteobacteria>gammaproteobacteria             Pseudomonas aeruginosa                                                          Putative cytoplasmic protein [Pseudomonas aeruginosa].
489221522       -                               238     bacteria>proteobacteria>gammaproteobacteria             Pseudomonas aeruginosa                                                          hypothetical protein [Pseudomonas aeruginosa].
497561968       -                               174     bacteria>proteobacteria>gammaproteobacteria             Pseudomonas aeruginosa                                                          hypothetical protein [Pseudomonas aeruginosa].
489224841       -                               203     bacteria>proteobacteria>gammaproteobacteria             Pseudomonas aeruginosa                                                          hypothetical protein [Pseudomonas aeruginosa].
386057446       -                               238     bacteria>proteobacteria>gammaproteobacteria             Pseudomonas aeruginosa M18                                                      hypothetical protein PAM18_1379 [Pseudomonas aeruginosa M18].
386067641       -                               238     bacteria>proteobacteria>gammaproteobacteria             Pseudomonas aeruginosa NCGM2.S1                                                 hypothetical protein NCGM2_4737 [Pseudomonas aeruginosa NCGM2.S1].
152983631       -                               238     bacteria>proteobacteria>gammaproteobacteria             Pseudomonas aeruginosa PA7                                                      hypothetical protein PSPA7_1535 [Pseudomonas aeruginosa PA7].
152985068       -                               203     bacteria>proteobacteria>gammaproteobacteria             Pseudomonas aeruginosa PA7                                                      hypothetical protein PSPA7_3914 [Pseudomonas aeruginosa PA7].
15598802        -                               238     bacteria>proteobacteria>gammaproteobacteria             Pseudomonas aeruginosa PAO1                                                     hypothetical protein PA3606 [Pseudomonas aeruginosa PAO1].
116051603       -                               238     bacteria>proteobacteria>gammaproteobacteria             Pseudomonas aeruginosa UCBPP-PA14                                               hypothetical protein PA14_17650 [Pseudomonas aeruginosa UCBPP-PA14].
116049370       -                               203     bacteria>proteobacteria>gammaproteobacteria             Pseudomonas aeruginosa UCBPP-PA14                                               hypothetical protein PA14_46020 [Pseudomonas aeruginosa UCBPP-PA14].
492058765       -                               236     bacteria>proteobacteria>gammaproteobacteria             Pseudomonas amygdali                                                            hypothetical protein [Pseudomonas amygdali].
497885898       -                               199     bacteria>proteobacteria>gammaproteobacteria             Pseudomonas amygdali                                                            hypothetical protein [Pseudomonas amygdali].
492071555       -                               199     bacteria>proteobacteria>gammaproteobacteria             Pseudomonas amygdali                                                            hypothetical protein [Pseudomonas amygdali].
488617735       -                               199     bacteria>proteobacteria>gammaproteobacteria             Pseudomonas amygdali                                                            hypothetical protein [Pseudomonas amygdali].
492051056       -                               236     bacteria>proteobacteria>gammaproteobacteria             Pseudomonas amygdali                                                            hypothetical protein [Pseudomonas amygdali].
491817633       -                               236     bacteria>proteobacteria>gammaproteobacteria             Pseudomonas avellanae                                                           hypothetical protein [Pseudomonas avellanae].
491818353       -                               199     bacteria>proteobacteria>gammaproteobacteria             Pseudomonas avellanae                                                           hypothetical protein [Pseudomonas avellanae].
330808165       -                               139     bacteria>proteobacteria>gammaproteobacteria             Pseudomonas brassicacearum subsp. brassicacearum NFM421                         hypothetical protein PSEBR_a1428, partial [Pseudomonas brassicacearum subsp. brassicacearum NFM421].
330807846       -                               249     bacteria>proteobacteria>gammaproteobacteria             Pseudomonas brassicacearum subsp. brassicacearum NFM421                         hypothetical protein PSEBR_a1127 [Pseudomonas brassicacearum subsp. brassicacearum NFM421].
496333264       -                               236     bacteria>proteobacteria>gammaproteobacteria             Pseudomonas chlororaphis                                                        hypothetical protein [Pseudomonas chlororaphis].
496333494       -                               203     bacteria>proteobacteria>gammaproteobacteria             Pseudomonas chlororaphis                                                        hypothetical protein [Pseudomonas chlororaphis].
496338151       -                               236     bacteria>proteobacteria>gammaproteobacteria             Pseudomonas chlororaphis                                                        hypothetical protein [Pseudomonas chlororaphis].
496338458       -                               205     bacteria>proteobacteria>gammaproteobacteria             Pseudomonas chlororaphis                                                        hypothetical protein [Pseudomonas chlororaphis].
492572073       -                               199     bacteria>proteobacteria>gammaproteobacteria             Pseudomonas coronafaciens                                                       hypothetical protein [Pseudomonas coronafaciens].
492574884       -                               236     bacteria>proteobacteria>gammaproteobacteria             Pseudomonas coronafaciens                                                       hypothetical protein [Pseudomonas coronafaciens].
472324800       -                               199     bacteria>proteobacteria>gammaproteobacteria             Pseudomonas denitrificans ATCC 13867                                            DTW domain-containing protein [Pseudomonas denitrificans ATCC 13867].
472324726       -                               241     bacteria>proteobacteria>gammaproteobacteria             Pseudomonas denitrificans ATCC 13867                                            hypothetical protein H681_07780 [Pseudomonas denitrificans ATCC 13867].
104782925       -                               197     bacteria>proteobacteria>gammaproteobacteria             Pseudomonas entomophila L48                                                     hypothetical protein PSEEN3930 [Pseudomonas entomophila L48].
104783099       -                               236     bacteria>proteobacteria>gammaproteobacteria             Pseudomonas entomophila L48                                                     hypothetical protein PSEEN4123 [Pseudomonas entomophila L48].
498249752       -                               201     bacteria>proteobacteria>gammaproteobacteria             Pseudomonas extremaustralis                                                     hypothetical protein [Pseudomonas extremaustralis].
498249296       -                               239     bacteria>proteobacteria>gammaproteobacteria             Pseudomonas extremaustralis                                                     hypothetical protein [Pseudomonas extremaustralis].
489315338       -                               197     bacteria>proteobacteria>gammaproteobacteria             Pseudomonas fluorescens                                                         hypothetical protein [Pseudomonas fluorescens].
489291445       -                               198     bacteria>proteobacteria>gammaproteobacteria             Pseudomonas fluorescens                                                         hypothetical protein [Pseudomonas fluorescens].
489285493       -                               199     bacteria>proteobacteria>gammaproteobacteria             Pseudomonas fluorescens                                                         hypothetical protein [Pseudomonas fluorescens].
489314894       -                               248     bacteria>proteobacteria>gammaproteobacteria             Pseudomonas fluorescens                                                         hypothetical protein [Pseudomonas fluorescens].
489323190       -                               198     bacteria>proteobacteria>gammaproteobacteria             Pseudomonas fluorescens                                                         hypothetical protein [Pseudomonas fluorescens].
489276446       -                               198     bacteria>proteobacteria>gammaproteobacteria             Pseudomonas fluorescens                                                         hypothetical protein [Pseudomonas fluorescens].
489264343       -                               239     bacteria>proteobacteria>gammaproteobacteria             Pseudomonas fluorescens                                                         hypothetical protein [Pseudomonas fluorescens].
489267603       -                               233     bacteria>proteobacteria>gammaproteobacteria             Pseudomonas fluorescens                                                         hypothetical protein [Pseudomonas fluorescens].
489324040       -                               188     bacteria>proteobacteria>gammaproteobacteria             Pseudomonas fluorescens                                                         DTW domain protein [Pseudomonas fluorescens].
489281418       -                               239     bacteria>proteobacteria>gammaproteobacteria             Pseudomonas fluorescens                                                         hypothetical protein [Pseudomonas fluorescens].
489277116       -                               248     bacteria>proteobacteria>gammaproteobacteria             Pseudomonas fluorescens                                                         hypothetical protein [Pseudomonas fluorescens].
387894959       -                               199     bacteria>proteobacteria>gammaproteobacteria             Pseudomonas fluorescens A506                                                    DTW domain protein [Pseudomonas fluorescens A506].
387892389       -                               239     bacteria>proteobacteria>gammaproteobacteria             Pseudomonas fluorescens A506                                                    DTW domain protein [Pseudomonas fluorescens A506].
378949438       -                               179     bacteria>proteobacteria>gammaproteobacteria             Pseudomonas fluorescens F113                                                    dtw domain-containing protein [Pseudomonas fluorescens F113].
378949133       -                               248     bacteria>proteobacteria>gammaproteobacteria             Pseudomonas fluorescens F113                                                    dtw protein [Pseudomonas fluorescens F113].
77457686        -                               197     bacteria>proteobacteria>gammaproteobacteria             Pseudomonas fluorescens Pf0-1                                                   hypothetical protein Pfl01_1459 [Pseudomonas fluorescens Pf0-1].
77457408        -                               247     bacteria>proteobacteria>gammaproteobacteria             Pseudomonas fluorescens Pf0-1                                                   hypothetical protein Pfl01_1181 [Pseudomonas fluorescens Pf0-1].
229588731       -                               239     bacteria>proteobacteria>gammaproteobacteria             Pseudomonas fluorescens SBW25                                                   hypothetical protein PFLU1193 [Pseudomonas fluorescens SBW25].
229591919       -                               201     bacteria>proteobacteria>gammaproteobacteria             Pseudomonas fluorescens SBW25                                                   hypothetical protein PFLU4508 [Pseudomonas fluorescens SBW25].
498342301       -                               202     bacteria>proteobacteria>gammaproteobacteria             Pseudomonas fragi                                                               hypothetical protein [Pseudomonas fragi].
498341531       -                               194     bacteria>proteobacteria>gammaproteobacteria             Pseudomonas fragi                                                               hypothetical protein [Pseudomonas fragi].
333899527       -                               236     bacteria>proteobacteria>gammaproteobacteria             Pseudomonas fulva 12-X                                                          DTW domain containing protein [Pseudomonas fulva 12-X].
333900108       -                               199     bacteria>proteobacteria>gammaproteobacteria             Pseudomonas fulva 12-X                                                          DTW domain containing protein [Pseudomonas fulva 12-X].
498135898       -                               199     bacteria>proteobacteria>gammaproteobacteria             Pseudomonas fuscovaginae                                                        hypothetical protein [Pseudomonas fuscovaginae].
498137119       -                               239     bacteria>proteobacteria>gammaproteobacteria             Pseudomonas fuscovaginae                                                        hypothetical protein [Pseudomonas fuscovaginae].
498148550       -                               252     bacteria>proteobacteria>gammaproteobacteria             Pseudomonas mandelii                                                            hypothetical protein [Pseudomonas mandelii].
498147846       -                               197     bacteria>proteobacteria>gammaproteobacteria             Pseudomonas mandelii                                                            hypothetical protein [Pseudomonas mandelii].
489337416       -                               197     bacteria>proteobacteria>gammaproteobacteria             Pseudomonas mendocina                                                           hypothetical protein [Pseudomonas mendocina].
489334471       -                               250     bacteria>proteobacteria>gammaproteobacteria             Pseudomonas mendocina                                                           hypothetical protein [Pseudomonas mendocina].
330502236       -                               240     bacteria>proteobacteria>gammaproteobacteria             Pseudomonas mendocina NK-01                                                     DTW domain-containing protein [Pseudomonas mendocina NK-01].
330503963       -                               168     bacteria>proteobacteria>gammaproteobacteria             Pseudomonas mendocina NK-01                                                     DTW domain-containing protein [Pseudomonas mendocina NK-01].
146306321       -                               244     bacteria>proteobacteria>gammaproteobacteria             Pseudomonas mendocina ymp                                                       DTW domain-containing protein [Pseudomonas mendocina ymp].
146306832       -                               197     bacteria>proteobacteria>gammaproteobacteria             Pseudomonas mendocina ymp                                                       DTW domain-containing protein [Pseudomonas mendocina ymp].
447915574       -                               188     bacteria>proteobacteria>gammaproteobacteria             Pseudomonas poae RE*1-1-14                                                      DTW domain protein [Pseudomonas poae RE*1-1-14].
447917685       -                               198     bacteria>proteobacteria>gammaproteobacteria             Pseudomonas poae RE*1-1-14                                                      hypothetical protein H045_13460 [Pseudomonas poae RE*1-1-14].
501678741       -                               224     bacteria>proteobacteria>gammaproteobacteria             Pseudomonas protegens CHA0                                                      DTW domain-containing protein YfiP [Pseudomonas protegens CHA0].
501679078       -                               198     bacteria>proteobacteria>gammaproteobacteria             Pseudomonas protegens CHA0                                                      DTW domain protein [Pseudomonas protegens CHA0].
70728968        -                               198     bacteria>proteobacteria>gammaproteobacteria             Pseudomonas protegens Pf-5                                                      DTW domain-containing protein [Pseudomonas protegens Pf-5].
70728618        -                               236     bacteria>proteobacteria>gammaproteobacteria             Pseudomonas protegens Pf-5                                                      DTW domain-containing protein [Pseudomonas protegens Pf-5].
490558436       -                               240     bacteria>proteobacteria>gammaproteobacteria             Pseudomonas pseudoalcaligenes                                                   hypothetical protein [Pseudomonas pseudoalcaligenes].
489550295       -                               203     bacteria>proteobacteria>gammaproteobacteria             Pseudomonas pseudoalcaligenes                                                   Hypothetical protein [Pseudomonas pseudoalcaligenes].
490556051       -                               202     bacteria>proteobacteria>gammaproteobacteria             Pseudomonas pseudoalcaligenes                                                   Putative cytoplasmic protein [Pseudomonas pseudoalcaligenes].
494276161       -                               245     bacteria>proteobacteria>gammaproteobacteria             Pseudomonas psychrotolerans                                                     hypothetical protein [Pseudomonas psychrotolerans].
494282024       -                               203     bacteria>proteobacteria>gammaproteobacteria             Pseudomonas psychrotolerans                                                     hypothetical protein [Pseudomonas psychrotolerans].
496878396       -                               236     bacteria>proteobacteria>gammaproteobacteria             Pseudomonas putida                                                              hypothetical protein [Pseudomonas putida].
496906466       -                               199     bacteria>proteobacteria>gammaproteobacteria             Pseudomonas putida                                                              DTW domain-containing protein [Pseudomonas putida].
7579038         -                               236     bacteria>proteobacteria>gammaproteobacteria             Pseudomonas putida                                                              unknown [Pseudomonas putida].
489351998       -                               194     bacteria>proteobacteria>gammaproteobacteria             Pseudomonas putida                                                              DTW domain-containing protein [Pseudomonas putida].
386010983       -                               199     bacteria>proteobacteria>gammaproteobacteria             Pseudomonas putida BIRD-1                                                       DTW domain-containing protein [Pseudomonas putida BIRD-1].
397696829       T1E_4089                        199     bacteria>proteobacteria>gammaproteobacteria             Pseudomonas putida DOT-T1E                                                      DTW domain-containing protein [Pseudomonas putida DOT-T1E].
148546631       -                               199     bacteria>proteobacteria>gammaproteobacteria             Pseudomonas putida F1                                                           DTW domain-containing protein [Pseudomonas putida F1].
148549289       -                               236     bacteria>proteobacteria>gammaproteobacteria             Pseudomonas putida F1                                                           DTW domain-containing protein [Pseudomonas putida F1].
167032248       -                               236     bacteria>proteobacteria>gammaproteobacteria             Pseudomonas putida GB-1                                                         DTW domain-containing protein [Pseudomonas putida GB-1].
167035021       -                               199     bacteria>proteobacteria>gammaproteobacteria             Pseudomonas putida GB-1                                                         DTW domain-containing protein [Pseudomonas putida GB-1].
431803775       -                               199     bacteria>proteobacteria>gammaproteobacteria             Pseudomonas putida HB3267                                                       DTW domain-containing protein [Pseudomonas putida HB3267].
26991205        -                               199     bacteria>proteobacteria>gammaproteobacteria             Pseudomonas putida KT2440                                                       DTW domain containing protein [Pseudomonas putida KT2440].
26988366        -                               236     bacteria>proteobacteria>gammaproteobacteria             Pseudomonas putida KT2440                                                       DTW domain containing protein [Pseudomonas putida KT2440].
395447886       YSA_07867                       210     bacteria>proteobacteria>gammaproteobacteria             Pseudomonas putida ND6                                                          DTW domain-containing protein [Pseudomonas putida ND6].
339486213       -                               236     bacteria>proteobacteria>gammaproteobacteria             Pseudomonas putida S16                                                          DTW domain-containing protein [Pseudomonas putida S16].
339488760       -                               199     bacteria>proteobacteria>gammaproteobacteria             Pseudomonas putida S16                                                          DTW domain-containing protein [Pseudomonas putida S16].
170722975       -                               199     bacteria>proteobacteria>gammaproteobacteria             Pseudomonas putida W619                                                         DTW domain-containing protein [Pseudomonas putida W619].
170723186       -                               236     bacteria>proteobacteria>gammaproteobacteria             Pseudomonas putida W619                                                         DTW domain-containing protein [Pseudomonas putida W619].
496672316       -                               203     bacteria>proteobacteria>gammaproteobacteria             Pseudomonas sp. 2_1_26                                                          hypothetical protein [Pseudomonas sp. 2_1_26].
495842396       -                               180     bacteria>proteobacteria>gammaproteobacteria             Pseudomonas sp. Chol1                                                           hypothetical protein [Pseudomonas sp. Chol1].
495842268       -                               236     bacteria>proteobacteria>gammaproteobacteria             Pseudomonas sp. Chol1                                                           hypothetical protein [Pseudomonas sp. Chol1].
495180348       -                               198     bacteria>proteobacteria>gammaproteobacteria             Pseudomonas sp. GM102                                                           hypothetical protein [Pseudomonas sp. GM102].
495182493       -                               247     bacteria>proteobacteria>gammaproteobacteria             Pseudomonas sp. GM102                                                           hypothetical protein [Pseudomonas sp. GM102].
495195891       -                               203     bacteria>proteobacteria>gammaproteobacteria             Pseudomonas sp. GM17                                                            hypothetical protein [Pseudomonas sp. GM17].
495199148       -                               236     bacteria>proteobacteria>gammaproteobacteria             Pseudomonas sp. GM17                                                            hypothetical protein [Pseudomonas sp. GM17].
495215785       -                               252     bacteria>proteobacteria>gammaproteobacteria             Pseudomonas sp. GM18                                                            hypothetical protein [Pseudomonas sp. GM18].
495213697       -                               198     bacteria>proteobacteria>gammaproteobacteria             Pseudomonas sp. GM18                                                            hypothetical protein [Pseudomonas sp. GM18].
495221219       -                               197     bacteria>proteobacteria>gammaproteobacteria             Pseudomonas sp. GM21                                                            hypothetical protein [Pseudomonas sp. GM21].
495222406       -                               252     bacteria>proteobacteria>gammaproteobacteria             Pseudomonas sp. GM21                                                            hypothetical protein [Pseudomonas sp. GM21].
495231825       -                               197     bacteria>proteobacteria>gammaproteobacteria             Pseudomonas sp. GM25                                                            hypothetical protein [Pseudomonas sp. GM25].
495230165       -                               247     bacteria>proteobacteria>gammaproteobacteria             Pseudomonas sp. GM25                                                            hypothetical protein [Pseudomonas sp. GM25].
495239610       -                               248     bacteria>proteobacteria>gammaproteobacteria             Pseudomonas sp. GM30                                                            hypothetical protein [Pseudomonas sp. GM30].
495237798       -                               198     bacteria>proteobacteria>gammaproteobacteria             Pseudomonas sp. GM30                                                            hypothetical protein [Pseudomonas sp. GM30].
495247759       -                               198     bacteria>proteobacteria>gammaproteobacteria             Pseudomonas sp. GM33                                                            hypothetical protein [Pseudomonas sp. GM33].
495246394       -                               252     bacteria>proteobacteria>gammaproteobacteria             Pseudomonas sp. GM33                                                            hypothetical protein [Pseudomonas sp. GM33].
495429287       -                               244     bacteria>proteobacteria>gammaproteobacteria             Pseudomonas sp. GM41(2012)                                                      hypothetical protein [Pseudomonas sp. GM41(2012)].
495428532       -                               198     bacteria>proteobacteria>gammaproteobacteria             Pseudomonas sp. GM41(2012)                                                      hypothetical protein [Pseudomonas sp. GM41(2012)].
495260161       -                               198     bacteria>proteobacteria>gammaproteobacteria             Pseudomonas sp. GM48                                                            hypothetical protein [Pseudomonas sp. GM48].
495265447       -                               252     bacteria>proteobacteria>gammaproteobacteria             Pseudomonas sp. GM48                                                            hypothetical protein [Pseudomonas sp. GM48].
495272155       -                               252     bacteria>proteobacteria>gammaproteobacteria             Pseudomonas sp. GM49                                                            hypothetical protein [Pseudomonas sp. GM49].
495279292       -                               198     bacteria>proteobacteria>gammaproteobacteria             Pseudomonas sp. GM49                                                            hypothetical protein [Pseudomonas sp. GM49].
495288785       -                               198     bacteria>proteobacteria>gammaproteobacteria             Pseudomonas sp. GM50                                                            hypothetical protein [Pseudomonas sp. GM50].
495285393       -                               247     bacteria>proteobacteria>gammaproteobacteria             Pseudomonas sp. GM50                                                            hypothetical protein [Pseudomonas sp. GM50].
495295053       -                               252     bacteria>proteobacteria>gammaproteobacteria             Pseudomonas sp. GM55                                                            hypothetical protein [Pseudomonas sp. GM55].
495293495       -                               198     bacteria>proteobacteria>gammaproteobacteria             Pseudomonas sp. GM55                                                            hypothetical protein [Pseudomonas sp. GM55].
495305272       -                               247     bacteria>proteobacteria>gammaproteobacteria             Pseudomonas sp. GM60                                                            hypothetical protein [Pseudomonas sp. GM60].
495309506       -                               198     bacteria>proteobacteria>gammaproteobacteria             Pseudomonas sp. GM60                                                            hypothetical protein [Pseudomonas sp. GM60].
495316220       -                               198     bacteria>proteobacteria>gammaproteobacteria             Pseudomonas sp. GM67                                                            hypothetical protein [Pseudomonas sp. GM67].
495314542       -                               247     bacteria>proteobacteria>gammaproteobacteria             Pseudomonas sp. GM67                                                            hypothetical protein [Pseudomonas sp. GM67].
495322067       -                               198     bacteria>proteobacteria>gammaproteobacteria             Pseudomonas sp. GM74                                                            hypothetical protein [Pseudomonas sp. GM74].
495329352       -                               252     bacteria>proteobacteria>gammaproteobacteria             Pseudomonas sp. GM74                                                            hypothetical protein [Pseudomonas sp. GM74].
495335909       -                               247     bacteria>proteobacteria>gammaproteobacteria             Pseudomonas sp. GM78                                                            hypothetical protein [Pseudomonas sp. GM78].
495335401       -                               198     bacteria>proteobacteria>gammaproteobacteria             Pseudomonas sp. GM78                                                            hypothetical protein [Pseudomonas sp. GM78].
495347982       -                               247     bacteria>proteobacteria>gammaproteobacteria             Pseudomonas sp. GM79                                                            hypothetical protein [Pseudomonas sp. GM79].
495345548       -                               198     bacteria>proteobacteria>gammaproteobacteria             Pseudomonas sp. GM79                                                            hypothetical protein [Pseudomonas sp. GM79].
495361528       -                               198     bacteria>proteobacteria>gammaproteobacteria             Pseudomonas sp. GM80                                                            hypothetical protein [Pseudomonas sp. GM80].
495352450       -                               249     bacteria>proteobacteria>gammaproteobacteria             Pseudomonas sp. GM80                                                            hypothetical protein [Pseudomonas sp. GM80].
495373245       -                               199     bacteria>proteobacteria>gammaproteobacteria             Pseudomonas sp. GM84                                                            hypothetical protein [Pseudomonas sp. GM84].
495369987       -                               236     bacteria>proteobacteria>gammaproteobacteria             Pseudomonas sp. GM84                                                            hypothetical protein [Pseudomonas sp. GM84].
498496700       -                               279     bacteria>proteobacteria>gammaproteobacteria             Pseudomonas sp. HPB0071                                                         hypothetical protein [Pseudomonas sp. HPB0071].
498494019       -                               203     bacteria>proteobacteria>gammaproteobacteria             Pseudomonas sp. HPB0071                                                         hypothetical protein [Pseudomonas sp. HPB0071].
497910609       -                               236     bacteria>proteobacteria>gammaproteobacteria             Pseudomonas sp. HYS                                                             hypothetical protein [Pseudomonas sp. HYS].
497908896       -                               199     bacteria>proteobacteria>gammaproteobacteria             Pseudomonas sp. HYS                                                             hypothetical protein [Pseudomonas sp. HYS].
489536990       -                               202     bacteria>proteobacteria>gammaproteobacteria             Pseudomonas sp. Lz4W                                                            DTW domain protein [Pseudomonas sp. Lz4W].
497309303       -                               197     bacteria>proteobacteria>gammaproteobacteria             Pseudomonas sp. M1                                                              DTW domain-containing protein [Pseudomonas sp. M1].
497302632       -                               246     bacteria>proteobacteria>gammaproteobacteria             Pseudomonas sp. M1                                                              DTW domain-containing protein [Pseudomonas sp. M1].
495641988       -                               194     bacteria>proteobacteria>gammaproteobacteria             Pseudomonas sp. M47T1                                                           hypothetical protein [Pseudomonas sp. M47T1].
495641862       -                               240     bacteria>proteobacteria>gammaproteobacteria             Pseudomonas sp. M47T1                                                           hypothetical protein [Pseudomonas sp. M47T1].
498493227       -                               203     bacteria>proteobacteria>gammaproteobacteria             Pseudomonas sp. P179                                                            hypothetical protein [Pseudomonas sp. P179].
498492580       -                               238     bacteria>proteobacteria>gammaproteobacteria             Pseudomonas sp. P179                                                            hypothetical protein [Pseudomonas sp. P179].
497855905       -                               236     bacteria>proteobacteria>gammaproteobacteria             Pseudomonas sp. PAMC 25886                                                      hypothetical protein [Pseudomonas sp. PAMC 25886].
497862322       -                               198     bacteria>proteobacteria>gammaproteobacteria             Pseudomonas sp. PAMC 25886                                                      hypothetical protein [Pseudomonas sp. PAMC 25886].
497894492       -                               196     bacteria>proteobacteria>gammaproteobacteria             Pseudomonas sp. R81                                                             hypothetical protein [Pseudomonas sp. R81].
498169044       -                               201     bacteria>proteobacteria>gammaproteobacteria             Pseudomonas sp. S9                                                              hypothetical protein [Pseudomonas sp. S9].
498176282       -                               237     bacteria>proteobacteria>gammaproteobacteria             Pseudomonas sp. S9                                                              hypothetical protein [Pseudomonas sp. S9].
497371829       -                               236     bacteria>proteobacteria>gammaproteobacteria             Pseudomonas sp. TJI-51                                                          hypothetical protein [Pseudomonas sp. TJI-51].
497369459       -                               199     bacteria>proteobacteria>gammaproteobacteria             Pseudomonas sp. TJI-51                                                          hypothetical protein [Pseudomonas sp. TJI-51].
426408043       -                               252     bacteria>proteobacteria>gammaproteobacteria             Pseudomonas sp. UW4                                                             hypothetical protein PputUW4_01130 [Pseudomonas sp. UW4].
426410852       -                               198     bacteria>proteobacteria>gammaproteobacteria             Pseudomonas sp. UW4                                                             DTW domain-containing protein [Pseudomonas sp. UW4].
489393472       -                               194     bacteria>proteobacteria>gammaproteobacteria             Pseudomonas stutzeri                                                            hypothetical protein [Pseudomonas stutzeri].
489384229       -                               180     bacteria>proteobacteria>gammaproteobacteria             Pseudomonas stutzeri                                                            hypothetical protein [Pseudomonas stutzeri].
489373856       -                               145     bacteria>proteobacteria>gammaproteobacteria             Pseudomonas stutzeri                                                            hypothetical protein [Pseudomonas stutzeri].
489379020       -                               238     bacteria>proteobacteria>gammaproteobacteria             Pseudomonas stutzeri                                                            hypothetical protein [Pseudomonas stutzeri].
489392885       -                               236     bacteria>proteobacteria>gammaproteobacteria             Pseudomonas stutzeri                                                            hypothetical protein [Pseudomonas stutzeri].
489387694       -                               194     bacteria>proteobacteria>gammaproteobacteria             Pseudomonas stutzeri                                                            hypothetical protein [Pseudomonas stutzeri].
489388385       -                               237     bacteria>proteobacteria>gammaproteobacteria             Pseudomonas stutzeri                                                            hypothetical protein [Pseudomonas stutzeri].
489383749       -                               238     bacteria>proteobacteria>gammaproteobacteria             Pseudomonas stutzeri                                                            hypothetical protein [Pseudomonas stutzeri].
146281888       -                               239     bacteria>proteobacteria>gammaproteobacteria             Pseudomonas stutzeri A1501                                                      hypothetical protein PST_1514 [Pseudomonas stutzeri A1501].
146282811       -                               192     bacteria>proteobacteria>gammaproteobacteria             Pseudomonas stutzeri A1501                                                      hypothetical protein PST_2470 [Pseudomonas stutzeri A1501].
339494447       -                               174     bacteria>proteobacteria>gammaproteobacteria             Pseudomonas stutzeri ATCC 17588 = LMG 11199                                     hypothetical protein PSTAB_2370 [Pseudomonas stutzeri ATCC 17588 = LMG 11199].
339493495       -                               239     bacteria>proteobacteria>gammaproteobacteria             Pseudomonas stutzeri ATCC 17588 = LMG 11199                                     hypothetical protein PSTAB_1418 [Pseudomonas stutzeri ATCC 17588 = LMG 11199].
392421676       A458_13130                      194     bacteria>proteobacteria>gammaproteobacteria             Pseudomonas stutzeri CCUG 29243                                                 hypothetical protein A458_13130 [Pseudomonas stutzeri CCUG 29243].
392420693       A458_08140                      236     bacteria>proteobacteria>gammaproteobacteria             Pseudomonas stutzeri CCUG 29243                                                 hypothetical protein A458_08140 [Pseudomonas stutzeri CCUG 29243].
397686792       PSJM300_08425                   153     bacteria>proteobacteria>gammaproteobacteria             Pseudomonas stutzeri DSM 10701                                                  hypothetical protein PSJM300_08425 [Pseudomonas stutzeri DSM 10701].
397687921       PSJM300_14100                   236     bacteria>proteobacteria>gammaproteobacteria             Pseudomonas stutzeri DSM 10701                                                  hypothetical protein PSJM300_14100 [Pseudomonas stutzeri DSM 10701].
386021177       -                               192     bacteria>proteobacteria>gammaproteobacteria             Pseudomonas stutzeri DSM 4166                                                   hypothetical protein PSTAA_2578 [Pseudomonas stutzeri DSM 4166].
431927966       -                               236     bacteria>proteobacteria>gammaproteobacteria             Pseudomonas stutzeri RCH2                                                       hypothetical protein Psest_2866 [Pseudomonas stutzeri RCH2].
431927002       -                               195     bacteria>proteobacteria>gammaproteobacteria             Pseudomonas stutzeri RCH2                                                       hypothetical protein Psest_1860 [Pseudomonas stutzeri RCH2].
492238582       -                               239     bacteria>proteobacteria>gammaproteobacteria             Pseudomonas synxantha                                                           hypothetical protein [Pseudomonas synxantha].
492250675       -                               201     bacteria>proteobacteria>gammaproteobacteria             Pseudomonas synxantha                                                           hypothetical protein [Pseudomonas synxantha].
489486039       -                               236     bacteria>proteobacteria>gammaproteobacteria             Pseudomonas syringae                                                            DTW protein [Pseudomonas syringae].
489401154       -                               123     bacteria>proteobacteria>gammaproteobacteria             Pseudomonas syringae                                                            DTW protein, partial [Pseudomonas syringae].
489437268       -                               170     bacteria>proteobacteria>gammaproteobacteria             Pseudomonas syringae                                                            DTW protein [Pseudomonas syringae].
489504469       -                               236     bacteria>proteobacteria>gammaproteobacteria             Pseudomonas syringae                                                            hypothetical protein [Pseudomonas syringae].
489464504       -                               199     bacteria>proteobacteria>gammaproteobacteria             Pseudomonas syringae                                                            hypothetical protein [Pseudomonas syringae].
489473847       -                               199     bacteria>proteobacteria>gammaproteobacteria             Pseudomonas syringae                                                            hypothetical protein [Pseudomonas syringae].
490551663       -                               199     bacteria>proteobacteria>gammaproteobacteria             Pseudomonas syringae                                                            DTW superfamily protein [Pseudomonas syringae].
498119423       -                               199     bacteria>proteobacteria>gammaproteobacteria             Pseudomonas syringae                                                            hypothetical protein [Pseudomonas syringae].
489528600       -                               236     bacteria>proteobacteria>gammaproteobacteria             Pseudomonas syringae                                                            hypothetical protein [Pseudomonas syringae].
490532414       -                               236     bacteria>proteobacteria>gammaproteobacteria             Pseudomonas syringae                                                            hypothetical protein [Pseudomonas syringae].
489433991       -                               236     bacteria>proteobacteria>gammaproteobacteria             Pseudomonas syringae                                                            DTW protein [Pseudomonas syringae].
489488802       -                               199     bacteria>proteobacteria>gammaproteobacteria             Pseudomonas syringae                                                            DTW protein [Pseudomonas syringae].
489499349       -                               199     bacteria>proteobacteria>gammaproteobacteria             Pseudomonas syringae                                                            hypothetical protein [Pseudomonas syringae].
489529653       -                               199     bacteria>proteobacteria>gammaproteobacteria             Pseudomonas syringae                                                            hypothetical protein [Pseudomonas syringae].
489513308       -                               199     bacteria>proteobacteria>gammaproteobacteria             Pseudomonas syringae                                                            hypothetical protein [Pseudomonas syringae].
489519942       -                               199     bacteria>proteobacteria>gammaproteobacteria             Pseudomonas syringae                                                            hypothetical protein [Pseudomonas syringae].
489510709       -                               236     bacteria>proteobacteria>gammaproteobacteria             Pseudomonas syringae                                                            hypothetical protein [Pseudomonas syringae].
498107850       -                               199     bacteria>proteobacteria>gammaproteobacteria             Pseudomonas syringae                                                            DTW [Pseudomonas syringae].
498118124       -                               236     bacteria>proteobacteria>gammaproteobacteria             Pseudomonas syringae                                                            hypothetical protein [Pseudomonas syringae].
489459556       -                               236     bacteria>proteobacteria>gammaproteobacteria             Pseudomonas syringae                                                            hypothetical protein [Pseudomonas syringae].
494455109       -                               199     bacteria>proteobacteria>gammaproteobacteria             Pseudomonas syringae group genomosp. 3                                          hypothetical protein [Pseudomonas syringae group genomosp. 3].
494460599       -                               236     bacteria>proteobacteria>gammaproteobacteria             Pseudomonas syringae group genomosp. 3                                          hypothetical protein [Pseudomonas syringae group genomosp. 3].
494461906       -                               197     bacteria>proteobacteria>gammaproteobacteria             Pseudomonas syringae group genomosp. 3                                          hypothetical protein [Pseudomonas syringae group genomosp. 3].
497896887       -                               236     bacteria>proteobacteria>gammaproteobacteria             Pseudomonas syringae group genomosp. 3                                          hypothetical protein [Pseudomonas syringae group genomosp. 3].
71734019        -                               236     bacteria>proteobacteria>gammaproteobacteria             Pseudomonas syringae pv. phaseolicola 1448A                                     hypothetical protein PSPPH_3801 [Pseudomonas syringae pv. phaseolicola 1448A].
71736404        -                               199     bacteria>proteobacteria>gammaproteobacteria             Pseudomonas syringae pv. phaseolicola 1448A                                     hypothetical protein PSPPH_3684 [Pseudomonas syringae pv. phaseolicola 1448A].
66044630        -                               236     bacteria>proteobacteria>gammaproteobacteria             Pseudomonas syringae pv. syringae B728a                                         hypothetical protein Psyr_1382 [Pseudomonas syringae pv. syringae B728a].
66046893        -                               199     bacteria>proteobacteria>gammaproteobacteria             Pseudomonas syringae pv. syringae B728a                                         hypothetical protein Psyr_3664 [Pseudomonas syringae pv. syringae B728a].
28868934        -                               199     bacteria>proteobacteria>gammaproteobacteria             Pseudomonas syringae pv. tomato str. DC3000                                     hypothetical protein PSPTO_1728 [Pseudomonas syringae pv. tomato str. DC3000].
28871172        -                               236     bacteria>proteobacteria>gammaproteobacteria             Pseudomonas syringae pv. tomato str. DC3000                                     hypothetical protein PSPTO_4029 [Pseudomonas syringae pv. tomato str. DC3000].
491022434       -                               200     bacteria>proteobacteria>gammaproteobacteria             Pseudomonas viridiflava                                                         hypothetical protein [Pseudomonas viridiflava].
491017388       -                               236     bacteria>proteobacteria>gammaproteobacteria             Pseudomonas viridiflava                                                         hypothetical protein [Pseudomonas viridiflava].
119947002       -                               239     bacteria>proteobacteria>gammaproteobacteria             Psychromonas ingrahamii 37                                                      DTW domain-containing protein [Psychromonas ingrahamii 37].
119944650       -                               208     bacteria>proteobacteria>gammaproteobacteria             Psychromonas ingrahamii 37                                                      DTW domain-containing protein [Psychromonas ingrahamii 37].
470480394       -                               205     bacteria>proteobacteria>gammaproteobacteria             Psychromonas sp. CNPT3                                                          DTW domain-containing protein [Psychromonas sp. CNPT3].
470478674       -                               204     bacteria>proteobacteria>gammaproteobacteria             Psychromonas sp. CNPT3                                                          DTW domain-containing protein [Psychromonas sp. CNPT3].
383188702       -                               234     bacteria>proteobacteria>gammaproteobacteria             Rahnella aquatilis CIP 78.65 = ATCC 33071                                       hypothetical protein Rahaq2_0777 [Rahnella aquatilis CIP 78.65 = ATCC 33071].
322831468       -                               234     bacteria>proteobacteria>gammaproteobacteria             Rahnella sp. Y9602                                                              DTW domain containing protein [Rahnella sp. Y9602].
481847819       -                               232     bacteria>proteobacteria>gammaproteobacteria             Raoultella ornithinolytica B6                                                   DTW domain-containing protein [Raoultella ornithinolytica B6].
495318480       -                               243     bacteria>proteobacteria>gammaproteobacteria             Reinekea blandensis                                                             hypothetical protein [Reinekea blandensis].
495495870       -                               242     bacteria>proteobacteria>gammaproteobacteria             Rheinheimera nanhaiensis                                                        hypothetical protein [Rheinheimera nanhaiensis].
496176179       -                               234     bacteria>proteobacteria>gammaproteobacteria             Rheinheimera sp. A13L                                                           hypothetical protein [Rheinheimera sp. A13L].
496174208       -                               175     bacteria>proteobacteria>gammaproteobacteria             Rheinheimera sp. A13L                                                           hypothetical protein [Rheinheimera sp. A13L].
90022563        -                               245     bacteria>proteobacteria>gammaproteobacteria             Saccharophagus degradans 2-40                                                   DTW domain protein [Saccharophagus degradans 2-40].
90020323        -                               183     bacteria>proteobacteria>gammaproteobacteria             Saccharophagus degradans 2-40                                                   DTW domain protein [Saccharophagus degradans 2-40].
340000310       -                               232     bacteria>proteobacteria>gammaproteobacteria             Salmonella bongori NCTC 12419                                                   hypothetical protein SBG_2368 [Salmonella bongori NCTC 12419].
487371962       -                               97      bacteria>proteobacteria>gammaproteobacteria             Salmonella enterica                                                             hypothetical protein [Salmonella enterica].
487377419       -                               153     bacteria>proteobacteria>gammaproteobacteria             Salmonella enterica                                                             hypothetical protein [Salmonella enterica].
486313470       -                               90      bacteria>proteobacteria>gammaproteobacteria             Salmonella enterica                                                             hypothetical protein, partial [Salmonella enterica].
487596036       -                               168     bacteria>proteobacteria>gammaproteobacteria             Salmonella enterica                                                             DTW domain-containing protein [Salmonella enterica].
446230735       -                               98      bacteria>proteobacteria>gammaproteobacteria             Salmonella enterica                                                             hypothetical protein, partial [Salmonella enterica].
446105792       -                               226     bacteria>proteobacteria>gammaproteobacteria             Salmonella enterica                                                             hypothetical protein [Salmonella enterica].
446105793       -                               226     bacteria>proteobacteria>gammaproteobacteria             Salmonella enterica                                                             hypothetical protein [Salmonella enterica].
487365847       -                               116     bacteria>proteobacteria>gammaproteobacteria             Salmonella enterica                                                             hypothetical protein, partial [Salmonella enterica].
446105778       -                               232     bacteria>proteobacteria>gammaproteobacteria             Salmonella enterica                                                             hypothetical protein [Salmonella enterica].
487697146       -                               129     bacteria>proteobacteria>gammaproteobacteria             Salmonella enterica                                                             hypothetical protein [Salmonella enterica].
446105779       -                               232     bacteria>proteobacteria>gammaproteobacteria             Salmonella enterica                                                             DTW domain-containing protein yfiP [Salmonella enterica].
446961360       -                               232     bacteria>proteobacteria>gammaproteobacteria             Salmonella enterica                                                             DTW domain-containing protein yfiP [Salmonella enterica].
487073946       -                               112     bacteria>proteobacteria>gammaproteobacteria             Salmonella enterica                                                             hypothetical protein, partial [Salmonella enterica].
446105783       -                               226     bacteria>proteobacteria>gammaproteobacteria             Salmonella enterica                                                             DTW domain-containing protein yfiP [Salmonella enterica].
489043952       -                               232     bacteria>proteobacteria>gammaproteobacteria             Salmonella enterica                                                             DTW domain-containing protein yfiP [Salmonella enterica].
446105780       -                               226     bacteria>proteobacteria>gammaproteobacteria             Salmonella enterica                                                             hypothetical protein [Salmonella enterica].
197248147       -                               226     bacteria>proteobacteria>gammaproteobacteria             Salmonella enterica subsp. enterica serovar Agona str. SL483                    DTW domain-containing protein [Salmonella enterica subsp. enterica serovar Agona str. SL483].
198242843       -                               226     bacteria>proteobacteria>gammaproteobacteria             Salmonella enterica subsp. enterica serovar Dublin str. CT_02021853             hypothetical protein SeD_A2978 [Salmonella enterica subsp. enterica serovar Dublin str. CT_02021853].
452123543       -                               226     bacteria>proteobacteria>gammaproteobacteria             Salmonella enterica subsp. enterica serovar Javiana str. CFSAN001992            DTW domain-containing protein [Salmonella enterica subsp. enterica serovar Javiana str. CFSAN001992].
194444694       -                               226     bacteria>proteobacteria>gammaproteobacteria             Salmonella enterica subsp. enterica serovar Newport str. SL254                  DTW domain-containing protein [Salmonella enterica subsp. enterica serovar Newport str. SL254].
56412527        SPA0267                         226     bacteria>proteobacteria>gammaproteobacteria             Salmonella enterica subsp. enterica serovar Paratyphi A str. ATCC 9150          hypothetical protein SPA0267 [Salmonella enterica subsp. enterica serovar Paratyphi A str. ATCC 9150].
161612630       -                               226     bacteria>proteobacteria>gammaproteobacteria             Salmonella enterica subsp. enterica serovar Paratyphi B str. SPB7               hypothetical protein SPAB_00325 [Salmonella enterica subsp. enterica serovar Paratyphi B str. SPB7].
194736464       -                               226     bacteria>proteobacteria>gammaproteobacteria             Salmonella enterica subsp. enterica serovar Schwarzengrund str. CVM19633        DTW domain-containing protein [Salmonella enterica subsp. enterica serovar Schwarzengrund str. CVM19633].
16761508        -                               226     bacteria>proteobacteria>gammaproteobacteria             Salmonella enterica subsp. enterica serovar Typhi str. CT18                     conserved hypothetical protein [Salmonella enterica subsp. enterica serovar Typhi str. CT18].
16765970        -                               226     bacteria>proteobacteria>gammaproteobacteria             Salmonella enterica subsp. enterica serovar Typhimurium str. LT2                hypothetical protein STM2650 [Salmonella enterica subsp. enterica serovar Typhimurium str. LT2].
409249701       SENTW_1228                      226     bacteria>proteobacteria>gammaproteobacteria             Salmonella enterica subsp. enterica serovar Weltevreden str. 2007-60-3289-1     DTW domain-containing protein yfiP [Salmonella enterica subsp. enterica serovar Weltevreden str. 2007-60-3289-1].
440232090       -                               231     bacteria>proteobacteria>gammaproteobacteria             Serratia marcescens FGI94                                                       hypothetical protein D781_3482 [Serratia marcescens FGI94].
448243622       -                               231     bacteria>proteobacteria>gammaproteobacteria             Serratia marcescens WW4                                                         hypothetical protein SMWW4_v1c38690 [Serratia marcescens WW4].
491097733       -                               236     bacteria>proteobacteria>gammaproteobacteria             Serratia odorifera                                                              hypothetical protein [Serratia odorifera].
493362177       -                               234     bacteria>proteobacteria>gammaproteobacteria             Serratia plymuthica                                                             hypothetical protein [Serratia plymuthica].
491092189       -                               234     bacteria>proteobacteria>gammaproteobacteria             Serratia plymuthica                                                             hypothetical protein [Serratia plymuthica].
157371982       -                               234     bacteria>proteobacteria>gammaproteobacteria             Serratia proteamaculans 568                                                     DTW domain-containing protein [Serratia proteamaculans 568].
333928771       -                               234     bacteria>proteobacteria>gammaproteobacteria             Serratia sp. AS12                                                               DTW domain-containing protein [Serratia sp. AS12].
497322727       -                               234     bacteria>proteobacteria>gammaproteobacteria             Serratia sp. M24T3                                                              hypothetical protein [Serratia sp. M24T3].
119774883       -                               252     bacteria>proteobacteria>gammaproteobacteria             Shewanella amazonensis SB2B                                                     hypothetical protein Sama_1748 [Shewanella amazonensis SB2B].
119776579       -                               209     bacteria>proteobacteria>gammaproteobacteria             Shewanella amazonensis SB2B                                                     hypothetical protein Sama_3447 [Shewanella amazonensis SB2B].
493005756       -                               254     bacteria>proteobacteria>gammaproteobacteria             Shewanella baltica                                                              hypothetical protein [Shewanella baltica].
493001103       -                               207     bacteria>proteobacteria>gammaproteobacteria             Shewanella baltica                                                              hypothetical protein [Shewanella baltica].
386324595       -                               272     bacteria>proteobacteria>gammaproteobacteria             Shewanella baltica BA175                                                        DTW domain containing protein [Shewanella baltica BA175].
386324535       -                               186     bacteria>proteobacteria>gammaproteobacteria             Shewanella baltica BA175                                                        DTW domain containing protein [Shewanella baltica BA175].
386326460       -                               207     bacteria>proteobacteria>gammaproteobacteria             Shewanella baltica BA175                                                        DTW domain containing protein [Shewanella baltica BA175].
126172452       -                               207     bacteria>proteobacteria>gammaproteobacteria             Shewanella baltica OS155                                                        DTW domain-containing protein [Shewanella baltica OS155].
126174539       -                               186     bacteria>proteobacteria>gammaproteobacteria             Shewanella baltica OS155                                                        DTW domain-containing protein [Shewanella baltica OS155].
153000846       -                               187     bacteria>proteobacteria>gammaproteobacteria             Shewanella baltica OS185                                                        DTW domain-containing protein [Shewanella baltica OS185].
153002636       -                               207     bacteria>proteobacteria>gammaproteobacteria             Shewanella baltica OS185                                                        DTW domain-containing protein [Shewanella baltica OS185].
160875494       -                               254     bacteria>proteobacteria>gammaproteobacteria             Shewanella baltica OS195                                                        DTW domain-containing protein [Shewanella baltica OS195].
160875554       -                               186     bacteria>proteobacteria>gammaproteobacteria             Shewanella baltica OS195                                                        DTW domain-containing protein [Shewanella baltica OS195].
217971407       -                               207     bacteria>proteobacteria>gammaproteobacteria             Shewanella baltica OS223                                                        DTW domain-containing protein [Shewanella baltica OS223].
217973196       -                               186     bacteria>proteobacteria>gammaproteobacteria             Shewanella baltica OS223                                                        DTW domain-containing protein [Shewanella baltica OS223].
378710587       -                               207     bacteria>proteobacteria>gammaproteobacteria             Shewanella baltica OS678                                                        DTW domain containing protein [Shewanella baltica OS678].
491638768       -                               174     bacteria>proteobacteria>gammaproteobacteria             Shewanella benthica                                                             hypothetical protein [Shewanella benthica].
491644225       -                               180     bacteria>proteobacteria>gammaproteobacteria             Shewanella benthica                                                             hypothetical protein [Shewanella benthica].
491639107       -                               252     bacteria>proteobacteria>gammaproteobacteria             Shewanella benthica                                                             hypothetical protein [Shewanella benthica].
91793136        -                               290     bacteria>proteobacteria>gammaproteobacteria             Shewanella denitrificans OS217                                                  hypothetical protein Sden_1780 [Shewanella denitrificans OS217].
91794840        -                               205     bacteria>proteobacteria>gammaproteobacteria             Shewanella denitrificans OS217                                                  hypothetical protein Sden_3493 [Shewanella denitrificans OS217].
91793964        -                               177     bacteria>proteobacteria>gammaproteobacteria             Shewanella denitrificans OS217                                                  hypothetical protein Sden_2613 [Shewanella denitrificans OS217].
114562484       -                               156     bacteria>proteobacteria>gammaproteobacteria             Shewanella frigidimarina NCIMB 400                                              DTW domain-containing protein [Shewanella frigidimarina NCIMB 400].
114561487       -                               203     bacteria>proteobacteria>gammaproteobacteria             Shewanella frigidimarina NCIMB 400                                              DTW domain-containing protein [Shewanella frigidimarina NCIMB 400].
114563093       -                               267     bacteria>proteobacteria>gammaproteobacteria             Shewanella frigidimarina NCIMB 400                                              DTW domain-containing protein [Shewanella frigidimarina NCIMB 400].
167623827       -                               138     bacteria>proteobacteria>gammaproteobacteria             Shewanella halifaxensis HAW-EB4                                                 DTW domain-containing protein [Shewanella halifaxensis HAW-EB4].
167623985       -                               258     bacteria>proteobacteria>gammaproteobacteria             Shewanella halifaxensis HAW-EB4                                                 DTW domain-containing protein [Shewanella halifaxensis HAW-EB4].
167622254       -                               197     bacteria>proteobacteria>gammaproteobacteria             Shewanella halifaxensis HAW-EB4                                                 DTW domain-containing protein [Shewanella halifaxensis HAW-EB4].
127513027       -                               189     bacteria>proteobacteria>gammaproteobacteria             Shewanella loihica PV-4                                                         DTW domain-containing protein [Shewanella loihica PV-4].
127514514       -                               200     bacteria>proteobacteria>gammaproteobacteria             Shewanella loihica PV-4                                                         DTW domain-containing protein [Shewanella loihica PV-4].
127512894       -                               249     bacteria>proteobacteria>gammaproteobacteria             Shewanella loihica PV-4                                                         DTW domain-containing protein [Shewanella loihica PV-4].
24376028        -                               197     bacteria>proteobacteria>gammaproteobacteria             Shewanella oneidensis MR-1                                                      DTW domain-containing protein [Shewanella oneidensis MR-1].
24373934        -                               251     bacteria>proteobacteria>gammaproteobacteria             Shewanella oneidensis MR-1                                                      DTW domain-containing protein [Shewanella oneidensis MR-1].
157961907       -                               253     bacteria>proteobacteria>gammaproteobacteria             Shewanella pealeana ATCC 700345                                                 DTW domain-containing protein [Shewanella pealeana ATCC 700345].
157962207       -                               171     bacteria>proteobacteria>gammaproteobacteria             Shewanella pealeana ATCC 700345                                                 DTW domain-containing protein [Shewanella pealeana ATCC 700345].
157963765       -                               223     bacteria>proteobacteria>gammaproteobacteria             Shewanella pealeana ATCC 700345                                                 DTW domain-containing protein [Shewanella pealeana ATCC 700345].
212556617       swp_2326                        167     bacteria>proteobacteria>gammaproteobacteria             Shewanella piezotolerans WP3                                                    DTW [Shewanella piezotolerans WP3].
212556794       swp_2508                        252     bacteria>proteobacteria>gammaproteobacteria             Shewanella piezotolerans WP3                                                    DTW [Shewanella piezotolerans WP3].
212558956       swp_4781                        200     bacteria>proteobacteria>gammaproteobacteria             Shewanella piezotolerans WP3                                                    DTW [Shewanella piezotolerans WP3].
386315711       -                               201     bacteria>proteobacteria>gammaproteobacteria             Shewanella putrefaciens 200                                                     DTW domain containing protein [Shewanella putrefaciens 200].
386313739       -                               256     bacteria>proteobacteria>gammaproteobacteria             Shewanella putrefaciens 200                                                     DTW domain containing protein [Shewanella putrefaciens 200].
386313828       -                               162     bacteria>proteobacteria>gammaproteobacteria             Shewanella putrefaciens 200                                                     DTW domain containing protein [Shewanella putrefaciens 200].
146291433       -                               201     bacteria>proteobacteria>gammaproteobacteria             Shewanella putrefaciens CN-32                                                   DTW domain-containing protein [Shewanella putrefaciens CN-32].
157375423       -                               247     bacteria>proteobacteria>gammaproteobacteria             Shewanella sediminis HAW-EB3                                                    DTW domain-containing protein [Shewanella sediminis HAW-EB3].
157375145       -                               174     bacteria>proteobacteria>gammaproteobacteria             Shewanella sediminis HAW-EB3                                                    DTW domain-containing protein [Shewanella sediminis HAW-EB3].
157373397       -                               197     bacteria>proteobacteria>gammaproteobacteria             Shewanella sediminis HAW-EB3                                                    DTW domain-containing protein [Shewanella sediminis HAW-EB3].
117920436       -                               254     bacteria>proteobacteria>gammaproteobacteria             Shewanella sp. ANA-3                                                            DTW domain-containing protein [Shewanella sp. ANA-3].
117922379       -                               200     bacteria>proteobacteria>gammaproteobacteria             Shewanella sp. ANA-3                                                            DTW domain-containing protein [Shewanella sp. ANA-3].
494921700       -                               253     bacteria>proteobacteria>gammaproteobacteria             Shewanella sp. HN-41                                                            hypothetical protein [Shewanella sp. HN-41].
494925902       -                               200     bacteria>proteobacteria>gammaproteobacteria             Shewanella sp. HN-41                                                            hypothetical protein [Shewanella sp. HN-41].
113970276       -                               254     bacteria>proteobacteria>gammaproteobacteria             Shewanella sp. MR-4                                                             DTW domain-containing protein [Shewanella sp. MR-4].
113972076       -                               188     bacteria>proteobacteria>gammaproteobacteria             Shewanella sp. MR-4                                                             DTW domain-containing protein [Shewanella sp. MR-4].
114049306       -                               188     bacteria>proteobacteria>gammaproteobacteria             Shewanella sp. MR-7                                                             DTW domain-containing protein [Shewanella sp. MR-7].
114047533       -                               254     bacteria>proteobacteria>gammaproteobacteria             Shewanella sp. MR-7                                                             DTW domain-containing protein [Shewanella sp. MR-7].
120598799       -                               256     bacteria>proteobacteria>gammaproteobacteria             Shewanella sp. W3-18-1                                                          DTW domain-containing protein [Shewanella sp. W3-18-1].
120600666       -                               237     bacteria>proteobacteria>gammaproteobacteria             Shewanella sp. W3-18-1                                                          DTW domain-containing protein [Shewanella sp. W3-18-1].
120598897       -                               164     bacteria>proteobacteria>gammaproteobacteria             Shewanella sp. W3-18-1                                                          DTW domain-containing protein [Shewanella sp. W3-18-1].
294139002       -                               198     bacteria>proteobacteria>gammaproteobacteria             Shewanella violacea DSS12                                                       hypothetical protein SVI_0231 [Shewanella violacea DSS12].
294140859       -                               252     bacteria>proteobacteria>gammaproteobacteria             Shewanella violacea DSS12                                                       hypothetical protein SVI_2088 [Shewanella violacea DSS12].
294141145       -                               166     bacteria>proteobacteria>gammaproteobacteria             Shewanella violacea DSS12                                                       hypothetical protein SVI_2374 [Shewanella violacea DSS12].
170726678       -                               249     bacteria>proteobacteria>gammaproteobacteria             Shewanella woodyi ATCC 51908                                                    DTW domain-containing protein [Shewanella woodyi ATCC 51908].
170726941       -                               168     bacteria>proteobacteria>gammaproteobacteria             Shewanella woodyi ATCC 51908                                                    DTW domain-containing protein [Shewanella woodyi ATCC 51908].
170724625       -                               174     bacteria>proteobacteria>gammaproteobacteria             Shewanella woodyi ATCC 51908                                                    DTW domain-containing protein [Shewanella woodyi ATCC 51908].
446258536       -                               249     bacteria>proteobacteria>gammaproteobacteria             Shigella boydii                                                                 DTW domain-containing protein yfiP [Shigella boydii].
446258539       -                               249     bacteria>proteobacteria>gammaproteobacteria             Shigella boydii                                                                 DTW domain-containing protein yfiP [Shigella boydii].
446258509       -                               249     bacteria>proteobacteria>gammaproteobacteria             Shigella boydii                                                                 DTW domain-containing protein yfiP [Shigella boydii].
491263077       -                               232     bacteria>proteobacteria>gammaproteobacteria             Shigella flexneri                                                               hypothetical protein [Shigella flexneri].
110806527       -                               249     bacteria>proteobacteria>gammaproteobacteria             Shigella flexneri 5 str. 8401                                                   hypothetical protein SFV_2646 [Shigella flexneri 5 str. 8401].
446258495       -                               249     bacteria>proteobacteria>gammaproteobacteria             Shigella sonnei                                                                 DTW domain-containing protein yfiP [Shigella sonnei].
387888303       -                               233     bacteria>proteobacteria>gammaproteobacteria             Shimwellia blattae DSM 4481 = NBRC 105725                                       hypothetical protein EBL_c09800 [Shimwellia blattae DSM 4481 = NBRC 105725].
410664018       -                               174     bacteria>proteobacteria>gammaproteobacteria             Simiduia agarivorans SA1 = DSM 21679                                            DTW domain containing protein [Simiduia agarivorans SA1 = DSM 21679].
410664933       -                               241     bacteria>proteobacteria>gammaproteobacteria             Simiduia agarivorans SA1 = DSM 21679                                            DTW domain-containing protein [Simiduia agarivorans SA1 = DSM 21679].
410665260       -                               195     bacteria>proteobacteria>gammaproteobacteria             Simiduia agarivorans SA1 = DSM 21679                                            DTW protein [Simiduia agarivorans SA1 = DSM 21679].
254788391       -                               236     bacteria>proteobacteria>gammaproteobacteria             Teredinibacter turnerae T7901                                                   Dtw [Teredinibacter turnerae T7901].
473827516       -                               238     bacteria>proteobacteria>gammaproteobacteria             Thalassolituus oleivorans MIL-1                                                 hypothetical protein TOL_0110 [Thalassolituus oleivorans MIL-1].
493504885       -                               180     bacteria>proteobacteria>gammaproteobacteria             Thioalkalimicrobium aerophilum                                                  hypothetical protein [Thioalkalimicrobium aerophilum].
334144243       Thicy_1153                      213     bacteria>proteobacteria>gammaproteobacteria             Thioalkalimicrobium cyclicum ALM1                                               DTW domain containing protein [Thioalkalimicrobium cyclicum ALM1].
78485208        Tcr_0863                        198     bacteria>proteobacteria>gammaproteobacteria             Thiomicrospira crunogena XCL-2                                                  hypothetical protein Tcr_0863 [Thiomicrospira crunogena XCL-2].
237808186       -                               225     bacteria>proteobacteria>gammaproteobacteria             Tolumonas auensis DSM 9187                                                      DTW domain containing protein [Tolumonas auensis DSM 9187].
446014578       -                               200     bacteria>proteobacteria>gammaproteobacteria             Vibrio                                                                          hypothetical protein [Vibrio].
446416269       -                               282     bacteria>proteobacteria>gammaproteobacteria             Vibrio albensis                                                                 hypothetical protein [Vibrio albensis].
446913168       -                               198     bacteria>proteobacteria>gammaproteobacteria             Vibrio albensis                                                                 hypothetical protein [Vibrio albensis].
491536214       -                               201     bacteria>proteobacteria>gammaproteobacteria             Vibrio alginolyticus                                                            hypothetical protein [Vibrio alginolyticus].
491524702       -                               204     bacteria>proteobacteria>gammaproteobacteria             Vibrio alginolyticus                                                            hypothetical protein [Vibrio alginolyticus].
491521109       -                               198     bacteria>proteobacteria>gammaproteobacteria             Vibrio alginolyticus                                                            hypothetical protein [Vibrio alginolyticus].
491526016       -                               180     bacteria>proteobacteria>gammaproteobacteria             Vibrio alginolyticus                                                            hypothetical protein [Vibrio alginolyticus].
491532912       -                               204     bacteria>proteobacteria>gammaproteobacteria             Vibrio alginolyticus                                                            DTW domain protein [Vibrio alginolyticus].
491526668       -                               91      bacteria>proteobacteria>gammaproteobacteria             Vibrio alginolyticus                                                            hypothetical protein [Vibrio alginolyticus].
491538578       -                               264     bacteria>proteobacteria>gammaproteobacteria             Vibrio alginolyticus                                                            hypothetical protein [Vibrio alginolyticus].
491531909       -                               249     bacteria>proteobacteria>gammaproteobacteria             Vibrio alginolyticus                                                            hypothetical protein [Vibrio alginolyticus].
336122719       VAA_01960                       201     bacteria>proteobacteria>gammaproteobacteria             Vibrio anguillarum 775                                                          hypothetical protein VAA_01960 [Vibrio anguillarum 775].
336123740       VAA_03322                       202     bacteria>proteobacteria>gammaproteobacteria             Vibrio anguillarum 775                                                          hypothetical protein VAA_03322 [Vibrio anguillarum 775].
336124210       VAA_1814                        243     bacteria>proteobacteria>gammaproteobacteria             Vibrio anguillarum 775                                                          hypothetical protein VAA_1814 [Vibrio anguillarum 775].
493934534       -                               237     bacteria>proteobacteria>gammaproteobacteria             Vibrio brasiliensis                                                             hypothetical protein [Vibrio brasiliensis].
493933782       -                               203     bacteria>proteobacteria>gammaproteobacteria             Vibrio brasiliensis                                                             hypothetical protein [Vibrio brasiliensis].
493937190       -                               209     bacteria>proteobacteria>gammaproteobacteria             Vibrio brasiliensis                                                             hypothetical protein [Vibrio brasiliensis].
498334995       -                               205     bacteria>proteobacteria>gammaproteobacteria             Vibrio campbellii                                                               hypothetical protein [Vibrio campbellii].
491677499       -                               198     bacteria>proteobacteria>gammaproteobacteria             Vibrio campbellii                                                               hypothetical protein [Vibrio campbellii].
491672270       -                               249     bacteria>proteobacteria>gammaproteobacteria             Vibrio campbellii                                                               cytoplasmic protein [Vibrio campbellii].
498333609       -                               194     bacteria>proteobacteria>gammaproteobacteria             Vibrio campbellii                                                               hypothetical protein [Vibrio campbellii].
491673766       -                               205     bacteria>proteobacteria>gammaproteobacteria             Vibrio campbellii                                                               hypothetical protein [Vibrio campbellii].
491572770       -                               198     bacteria>proteobacteria>gammaproteobacteria             Vibrio campbellii                                                               hypothetical protein [Vibrio campbellii].
491570058       -                               249     bacteria>proteobacteria>gammaproteobacteria             Vibrio campbellii                                                               hypothetical protein [Vibrio campbellii].
498336421       -                               198     bacteria>proteobacteria>gammaproteobacteria             Vibrio campbellii                                                               hypothetical protein [Vibrio campbellii].
156974538       -                               194     bacteria>proteobacteria>gammaproteobacteria             Vibrio campbellii ATCC BAA-1116                                                 hypothetical protein VIBHAR_02256 [Vibrio campbellii ATCC BAA-1116].
156973719       -                               171     bacteria>proteobacteria>gammaproteobacteria             Vibrio campbellii ATCC BAA-1116                                                 hypothetical protein VIBHAR_01424 [Vibrio campbellii ATCC BAA-1116].
156972652       -                               170     bacteria>proteobacteria>gammaproteobacteria             Vibrio campbellii ATCC BAA-1116                                                 hypothetical protein VIBHAR_00304 [Vibrio campbellii ATCC BAA-1116].
497286322       -                               150     bacteria>proteobacteria>gammaproteobacteria             Vibrio caribbenthicus                                                           hypothetical protein [Vibrio caribbenthicus].
497288185       -                               204     bacteria>proteobacteria>gammaproteobacteria             Vibrio caribbenthicus                                                           hypothetical protein [Vibrio caribbenthicus].
497285492       -                               198     bacteria>proteobacteria>gammaproteobacteria             Vibrio caribbenthicus                                                           hypothetical protein [Vibrio caribbenthicus].
446416266       -                               282     bacteria>proteobacteria>gammaproteobacteria             Vibrio cholerae                                                                 hypothetical protein [Vibrio cholerae].
446416267       -                               282     bacteria>proteobacteria>gammaproteobacteria             Vibrio cholerae                                                                 hypothetical protein [Vibrio cholerae].
487888447       -                               198     bacteria>proteobacteria>gammaproteobacteria             Vibrio cholerae                                                                 hypothetical protein [Vibrio cholerae].
487873801       -                               142     bacteria>proteobacteria>gammaproteobacteria             Vibrio cholerae                                                                 hypothetical protein [Vibrio cholerae].
446416261       -                               282     bacteria>proteobacteria>gammaproteobacteria             Vibrio cholerae                                                                 hypothetical protein [Vibrio cholerae].
487938945       -                               110     bacteria>proteobacteria>gammaproteobacteria             Vibrio cholerae                                                                 hypothetical protein [Vibrio cholerae].
446416264       -                               282     bacteria>proteobacteria>gammaproteobacteria             Vibrio cholerae                                                                 hypothetical protein [Vibrio cholerae].
487840318       -                               198     bacteria>proteobacteria>gammaproteobacteria             Vibrio cholerae                                                                 hypothetical protein [Vibrio cholerae].
488091205       -                               198     bacteria>proteobacteria>gammaproteobacteria             Vibrio cholerae                                                                 hypothetical protein [Vibrio cholerae].
487824915       -                               198     bacteria>proteobacteria>gammaproteobacteria             Vibrio cholerae                                                                 hypothetical protein [Vibrio cholerae].
446416265       -                               282     bacteria>proteobacteria>gammaproteobacteria             Vibrio cholerae                                                                 hypothetical protein [Vibrio cholerae].
446339798       -                               110     bacteria>proteobacteria>gammaproteobacteria             Vibrio cholerae                                                                 hypothetical protein [Vibrio cholerae].
446416268       -                               282     bacteria>proteobacteria>gammaproteobacteria             Vibrio cholerae                                                                 hypothetical protein [Vibrio cholerae].
446913167       -                               210     bacteria>proteobacteria>gammaproteobacteria             Vibrio cholerae                                                                 hypothetical protein [Vibrio cholerae].
446416262       -                               282     bacteria>proteobacteria>gammaproteobacteria             Vibrio cholerae                                                                 hypothetical protein [Vibrio cholerae].
487811341       -                               260     bacteria>proteobacteria>gammaproteobacteria             Vibrio cholerae                                                                 hypothetical protein [Vibrio cholerae].
446913166       -                               198     bacteria>proteobacteria>gammaproteobacteria             Vibrio cholerae                                                                 hypothetical protein [Vibrio cholerae].
487850000       -                               143     bacteria>proteobacteria>gammaproteobacteria             Vibrio cholerae                                                                 hypothetical protein [Vibrio cholerae].
487921527       -                               110     bacteria>proteobacteria>gammaproteobacteria             Vibrio cholerae                                                                 hypothetical protein [Vibrio cholerae].
487956734       -                               198     bacteria>proteobacteria>gammaproteobacteria             Vibrio cholerae                                                                 hypothetical protein [Vibrio cholerae].
446014571       -                               200     bacteria>proteobacteria>gammaproteobacteria             Vibrio cholerae                                                                 hypothetical protein [Vibrio cholerae].
446913176       -                               198     bacteria>proteobacteria>gammaproteobacteria             Vibrio cholerae                                                                 hypothetical protein [Vibrio cholerae].
446014573       -                               200     bacteria>proteobacteria>gammaproteobacteria             Vibrio cholerae                                                                 hypothetical protein [Vibrio cholerae].
446913175       -                               198     bacteria>proteobacteria>gammaproteobacteria             Vibrio cholerae                                                                 hypothetical protein [Vibrio cholerae].
446014575       -                               200     bacteria>proteobacteria>gammaproteobacteria             Vibrio cholerae                                                                 hypothetical protein [Vibrio cholerae].
446014576       -                               200     bacteria>proteobacteria>gammaproteobacteria             Vibrio cholerae                                                                 hypothetical protein [Vibrio cholerae].
446014577       -                               200     bacteria>proteobacteria>gammaproteobacteria             Vibrio cholerae                                                                 hypothetical protein [Vibrio cholerae].
446913164       -                               198     bacteria>proteobacteria>gammaproteobacteria             Vibrio cholerae                                                                 hypothetical protein [Vibrio cholerae].
446014579       -                               200     bacteria>proteobacteria>gammaproteobacteria             Vibrio cholerae                                                                 hypothetical protein [Vibrio cholerae].
446014581       -                               200     bacteria>proteobacteria>gammaproteobacteria             Vibrio cholerae                                                                 hypothetical protein [Vibrio cholerae].
446913163       -                               198     bacteria>proteobacteria>gammaproteobacteria             Vibrio cholerae                                                                 hypothetical protein [Vibrio cholerae].
446913174       -                               198     bacteria>proteobacteria>gammaproteobacteria             Vibrio cholerae                                                                 hypothetical protein [Vibrio cholerae].
446014584       -                               200     bacteria>proteobacteria>gammaproteobacteria             Vibrio cholerae                                                                 hypothetical protein [Vibrio cholerae].
446913173       -                               198     bacteria>proteobacteria>gammaproteobacteria             Vibrio cholerae                                                                 hypothetical protein [Vibrio cholerae].
446913172       -                               198     bacteria>proteobacteria>gammaproteobacteria             Vibrio cholerae                                                                 hypothetical protein [Vibrio cholerae].
487840971       -                               200     bacteria>proteobacteria>gammaproteobacteria             Vibrio cholerae                                                                 hypothetical protein [Vibrio cholerae].
487888140       -                               200     bacteria>proteobacteria>gammaproteobacteria             Vibrio cholerae                                                                 hypothetical protein [Vibrio cholerae].
487958358       -                               200     bacteria>proteobacteria>gammaproteobacteria             Vibrio cholerae                                                                 hypothetical protein [Vibrio cholerae].
487854366       -                               224     bacteria>proteobacteria>gammaproteobacteria             Vibrio cholerae                                                                 hypothetical protein [Vibrio cholerae].
446416273       -                               282     bacteria>proteobacteria>gammaproteobacteria             Vibrio cholerae                                                                 hypothetical protein [Vibrio cholerae].
446913165       -                               198     bacteria>proteobacteria>gammaproteobacteria             Vibrio cholerae                                                                 hypothetical protein [Vibrio cholerae].
446432368       -                               273     bacteria>proteobacteria>gammaproteobacteria             Vibrio cholerae                                                                 hypothetical protein [Vibrio cholerae].
446913171       -                               198     bacteria>proteobacteria>gammaproteobacteria             Vibrio cholerae                                                                 hypothetical protein [Vibrio cholerae].
446432369       -                               273     bacteria>proteobacteria>gammaproteobacteria             Vibrio cholerae                                                                 hypothetical protein [Vibrio cholerae].
446416270       -                               282     bacteria>proteobacteria>gammaproteobacteria             Vibrio cholerae                                                                 hypothetical protein [Vibrio cholerae].
446432370       -                               273     bacteria>proteobacteria>gammaproteobacteria             Vibrio cholerae                                                                 hypothetical protein [Vibrio cholerae].
446913160       -                               198     bacteria>proteobacteria>gammaproteobacteria             Vibrio cholerae                                                                 hypothetical protein [Vibrio cholerae].
446416271       -                               282     bacteria>proteobacteria>gammaproteobacteria             Vibrio cholerae                                                                 hypothetical protein [Vibrio cholerae].
446913169       -                               198     bacteria>proteobacteria>gammaproteobacteria             Vibrio cholerae                                                                 hypothetical protein [Vibrio cholerae].
446416272       -                               282     bacteria>proteobacteria>gammaproteobacteria             Vibrio cholerae                                                                 hypothetical protein [Vibrio cholerae].
446432371       -                               273     bacteria>proteobacteria>gammaproteobacteria             Vibrio cholerae                                                                 hypothetical protein [Vibrio cholerae].
487840886       -                               273     bacteria>proteobacteria>gammaproteobacteria             Vibrio cholerae                                                                 hypothetical protein [Vibrio cholerae].
446021501       -                               198     bacteria>proteobacteria>gammaproteobacteria             Vibrio cholerae                                                                 hypothetical protein [Vibrio cholerae].
487885971       -                               273     bacteria>proteobacteria>gammaproteobacteria             Vibrio cholerae                                                                 hypothetical protein [Vibrio cholerae].
446416263       -                               282     bacteria>proteobacteria>gammaproteobacteria             Vibrio cholerae                                                                 hypothetical protein [Vibrio cholerae].
487931881       -                               110     bacteria>proteobacteria>gammaproteobacteria             Vibrio cholerae                                                                 hypothetical protein [Vibrio cholerae].
487814959       -                               245     bacteria>proteobacteria>gammaproteobacteria             Vibrio cholerae                                                                 hypothetical protein [Vibrio cholerae].
487847752       -                               185     bacteria>proteobacteria>gammaproteobacteria             Vibrio cholerae                                                                 hypothetical protein [Vibrio cholerae].
487820805       -                               260     bacteria>proteobacteria>gammaproteobacteria             Vibrio cholerae                                                                 hypothetical protein [Vibrio cholerae].
446416260       -                               282     bacteria>proteobacteria>gammaproteobacteria             Vibrio cholerae                                                                 hypothetical protein [Vibrio cholerae].
446913161       -                               206     bacteria>proteobacteria>gammaproteobacteria             Vibrio cholerae                                                                 hypothetical protein [Vibrio cholerae].
446850047       -                               283     bacteria>proteobacteria>gammaproteobacteria             Vibrio cholerae                                                                 hypothetical protein [Vibrio cholerae].
446014582       -                               200     bacteria>proteobacteria>gammaproteobacteria             Vibrio cholerae                                                                 hypothetical protein [Vibrio cholerae].
384424605       VCLMA_A1329                     273     bacteria>proteobacteria>gammaproteobacteria             Vibrio cholerae LMA3984-4                                                       hypothetical protein VCLMA_A1329 [Vibrio cholerae LMA3984-4].
384423479       VCLMA_A0121                     198     bacteria>proteobacteria>gammaproteobacteria             Vibrio cholerae LMA3984-4                                                       hypothetical protein VCLMA_A0121 [Vibrio cholerae LMA3984-4].
384424964       VCLMA_A1721                     200     bacteria>proteobacteria>gammaproteobacteria             Vibrio cholerae LMA3984-4                                                       hypothetical protein VCLMA_A1721 [Vibrio cholerae LMA3984-4].
15641982        -                               200     bacteria>proteobacteria>gammaproteobacteria             Vibrio cholerae O1 biovar El Tor str. N16961                                    hypothetical protein VC1980 [Vibrio cholerae O1 biovar El Tor str. N16961].
15640162        -                               198     bacteria>proteobacteria>gammaproteobacteria             Vibrio cholerae O1 biovar El Tor str. N16961                                    hypothetical protein VC0131 [Vibrio cholerae O1 biovar El Tor str. N16961].
494016907       -                               160     bacteria>proteobacteria>gammaproteobacteria             Vibrio coralliilyticus                                                          hypothetical protein [Vibrio coralliilyticus].
494020040       -                               262     bacteria>proteobacteria>gammaproteobacteria             Vibrio coralliilyticus                                                          hypothetical protein [Vibrio coralliilyticus].
494014964       -                               199     bacteria>proteobacteria>gammaproteobacteria             Vibrio coralliilyticus                                                          hypothetical protein [Vibrio coralliilyticus].
498125375       -                               199     bacteria>proteobacteria>gammaproteobacteria             Vibrio cyclitrophicus                                                           hypothetical protein [Vibrio cyclitrophicus].
498124613       -                               198     bacteria>proteobacteria>gammaproteobacteria             Vibrio cyclitrophicus                                                           hypothetical protein [Vibrio cyclitrophicus].
498126748       -                               246     bacteria>proteobacteria>gammaproteobacteria             Vibrio cyclitrophicus                                                           hypothetical protein [Vibrio cyclitrophicus].
172087706       VF_1409                         247     bacteria>proteobacteria>gammaproteobacteria             Vibrio fischeri ES114                                                           hypothetical protein VF_1409 [Vibrio fischeri ES114].
59710695        VF_0088                         199     bacteria>proteobacteria>gammaproteobacteria             Vibrio fischeri ES114                                                           cytoplasmic protein [Vibrio fischeri ES114].
59712290        VF_1683                         207     bacteria>proteobacteria>gammaproteobacteria             Vibrio fischeri ES114                                                           cytoplasmic protein [Vibrio fischeri ES114].
197334856       -                               247     bacteria>proteobacteria>gammaproteobacteria             Vibrio fischeri MJ11                                                            DTW domain containing protein [Vibrio fischeri MJ11].
197334777       -                               207     bacteria>proteobacteria>gammaproteobacteria             Vibrio fischeri MJ11                                                            hypothetical protein VFMJ11_1807 [Vibrio fischeri MJ11].
197333987       -                               195     bacteria>proteobacteria>gammaproteobacteria             Vibrio fischeri MJ11                                                            hypothetical protein VFMJ11_0086 [Vibrio fischeri MJ11].
490862335       -                               181     bacteria>proteobacteria>gammaproteobacteria             Vibrio furnissii                                                                hypothetical protein [Vibrio furnissii].
490863010       -                               162     bacteria>proteobacteria>gammaproteobacteria             Vibrio furnissii                                                                hypothetical protein [Vibrio furnissii].
490863849       -                               191     bacteria>proteobacteria>gammaproteobacteria             Vibrio furnissii                                                                hypothetical protein [Vibrio furnissii].
375130936       -                               245     bacteria>proteobacteria>gammaproteobacteria             Vibrio furnissii NCTC 11218                                                     hypothetical protein [Vibrio furnissii NCTC 11218].
375129580       -                               181     bacteria>proteobacteria>gammaproteobacteria             Vibrio furnissii NCTC 11218                                                     hypothetical protein [Vibrio furnissii NCTC 11218].
375130428       -                               203     bacteria>proteobacteria>gammaproteobacteria             Vibrio furnissii NCTC 11218                                                     hypothetical protein [Vibrio furnissii NCTC 11218].
491594459       -                               198     bacteria>proteobacteria>gammaproteobacteria             Vibrio harveyi                                                                  hypothetical protein [Vibrio harveyi].
491589018       -                               205     bacteria>proteobacteria>gammaproteobacteria             Vibrio harveyi                                                                  hypothetical protein [Vibrio harveyi].
[truncated: 1,089,481 more chars]
